# Comparative Analysis of Milk Triglycerides Profile between Jaffarabadi Buffalo and Holstein Friesian Cow

**DOI:** 10.3390/metabo10120507

**Published:** 2020-12-11

**Authors:** Aparna Verma, Ningombam Sanjib Meitei, Prakash U. Gajbhiye, Mark J. Raftery, Kiran Ambatipudi

**Affiliations:** 1 Department of Biotechnology, Indian Institute of Technology Roorkee, Roorkee 247667, India; averma@bt.iitr.ac.in; 2 Luhup Private Limited, Indore 452001, India; sanjibmeiteicha@gmail.com; 3 Ningombam Angouton Memorial Trust, Imphal East, Manipur 795008, India; 4 Cattle Breeding Farm, Junagadh Agricultural University, Junagadh 362001, India; pgajbhiye@jau.ac.in; 5 Bioanalytical Mass Spectrometry Facility, University of New South Wales, Sydney 2052, Australia; m.raftery@unsw.edu.au

**Keywords:** bovine milk, lipidomics, Jaffarabadi buffalo, Holstein Friesian cow, LIPIDMAPS

## Abstract

Milk lipids are known for a variety of biological functions, however; little is known about compositional variation across breeds, especially for Jaffarabadi buffalo, an indigenous Indian breed. Systematic profiling of extracted milk lipids was performed by mass spectrometry across summer and winter in Holstein Friesian cow and Jaffarabadi buffalo. Extensive MS/MS spectral analysis for the identification (ID) of probable lipid species using software followed by manual verification and grading of each assigned lipid species enabled ID based on (a) parent ion, (b) head group, and (c) partial/full acyl characteristic ions for comparative profiling of triacylglycerols between the breeds. Additionally, new triacylglycerol species with short-chain fatty acids were reported by manual interpretation of MS/MS spectra and comparison with curated repositories. Collectively, 1093 triacylglycerol species belonging to 141 unique sum compositions between the replicates of both the animal groups were identified. Relative quantitation at sum composition level followed by statistical analyses revealed changes in relative abundances of triacylglycerol species due to breed, season, and interaction effect of the two. Significant changes in triacylglycerols were observed between breeds (81%) and seasons (59%). When the interaction effect is statistically significant, a higher number of triacylglycerols species in Jaffarabadi has lesser seasonal variation than Holstein Friesian.

## 1. Introduction

Milk is a complete nutritious drink composed of proteins, carbohydrates, fats, vitamins, and a range of bioactivities influencing the human health of all age groups in a positive way [1,2]. Milk composition is influenced by several factors such as breed, environmental conditions, lactation stage, and animal’s nutritional status [3,4]. To date, the majority of lipidomics studies have focused on improving dairy nutritional management strategies to enhance milk quality and production by improving pasteurization and preservation techniques to extend milk shelf-life and to remove bacteria, spores, and somatic cells from milk [5]. However, the role of different exogenous (e.g., season) and endogenous (e.g., breed) factors influencing milk composition, particularly lipids, a potential source of functional food component of milk and dairy products, has been limited in both cows and buffaloes [6,7].

Milk lipids play a crucial role in influencing the physical, chemical and technological properties of milk, altering the fatty acid (FA)-health relationship [8,9]. For instance, linoleic acid (LA) and α-linolenic acid (ALA) are the most common FAs acting as precursors for the synthesis of inflammatory mediators (e.g., prostaglandins and leukotrienes) and constraining the development of the nervous system in infants before birth [10,11]. Intake of bovine milk has declined despite its beneficial effects due to the undesirable effect of a high proportion of saturated (e.g., palmitic acid, myristic acids) [12,13] and trans-FAs that induce cardiovascular diseases [14], atherosclerosis [15,16], Alzheimer’s and lung cancer [17]. In contrast, the demand for milk with polyunsaturated fatty acids (PUFA) has gone up due to their immune-enhancing properties and reducing the risk of cardiovascular diseases [17,18]. Similarly, milk with polar lipids has demonstrated significant biological effects such as anti-inflammatory and anticarcinogenic activity [19]. Consequently, milk from different mammalian species has been investigated to identify different lipid species, which could have a maximum beneficial effect on human health [20]. In fact, the ever-growing literature suggests tremendous beneficial effects of bovine lipids to humans; however, little is known about the natural variation of milk fat to manipulate its concentration in milk in a positive way.

Several studies have identified milk lipids using gas chromatography coupled with a mass spectrometer (GC-MS) [21,22,23,24], but only a few lipids could be identified without derivatization, resulting in low lipid coverage [22]. In contrast, Knittelfelder (2014) [25] reported the identification and quantification of previously unidentified FAs using liquid chromatography coupled tandem mass spectrometry (LC-MS/MS), demonstrating its potential as a robust discovery and validation tool. Recently, the application of the Q-Exactive Orbitrap mass spectrometer has been used to investigate the variation and metabolism of important polar lipids in bovine and human milk [20]. Similarly, lipidomics of goat, soya and bovine milk by ultra-performance liquid chromatography (UPLC)-Q-Exactive Orbitrap mass spectrometry identified lipids as biomarkers, thereby differentiating milk types for authentication and detection of milk fraud [26].

Although cow’s milk is probably the most frequently consumed and economically important milk type, sheep, goat and buffalo milk consumption continues to rise [26,27]. Jaffarabadi (J) buffalo and Holstein Friesian (HF) cow are recognized as two important milk breeds and are significant contributors of milk to the Indian dairy industry. Milk fat is the major factor that determines the organoleptic quality as well as the commercial price of the milk. Buffalo milk has a fat content (weight percentage of the total lipids) of approximately (7–8), that is higher than a cow (3.3–4.7), and goat (4.1–4.5) [28]. Interestingly, J buffalo milk has an average fat content of 7.53% which is far higher than the typical fat content of the other buffalo breeds (detailed fat contents measured in different lactation stages are provided in the following section, “Sample Collection”). The milk fat consists mainly of triglycerides (approximately 98%) while other milk lipids are diacylglycerol (approximately 2% of the lipid fraction), cholesterol (less than 0.5%), phospholipids (approximately 1%) and free fatty acids (FFA) (approximately 0.1) [29]. In addition, there are trace amounts of ether lipids, hydrocarbons, fat-soluble vitamins, flavor compounds, and compounds introduced by the feed [30]. Since triglycerides account for approximately 98% of the total fat, they have a significant and direct effect on the properties of milk fat, for example, hydrophobicity, density, and melting characteristics.

Thus, an objective of the present study was to maximize milk triglycerides identification/quantitation using liquid chromatography coupled with a high-resolution accurate mass (HRAM)—mass spectrometry based lipidomic workflow, automated assignment of MS/MS spectra with probable lipid species, and a stepwise manual interpretation of mass spectra to document variation between J buffalo and HF cow across summer and winter. Milk samples were collected from four different groups: Jaffarabadi winter (JW), Jaffarabadi summer (JS), HF winter (HFW), HF summer (HFS). Lipids isolated using the Folch method were subjected to label-free quantification using mass spectrometry (MS), resulted in the identification of 1145 lipids, of which 170 and 187 were exclusively identified in J and HF, respectively. Results of the present study demonstrate that lipid abundance is breed- and season-dependent, and consideration of this information is critical for estimating milk’s nutritional value as well as to identify potential markers of milk fraud impacting human health.

## 2. Results and Discussion

### 2.1. Cumulative Lipid Identifications

The UPLC/MS-based lipidomics relies on compressive data analysis for success. A variety of commercial packages and freeware approaches have been developed and implemented with vendor-neutral software allowing analysis of data acquired from any mass spectrometer software [31]. Here, we use SimLipid’s inbuilt functionality for automated assignment of MS/MS spectra with possible lipid species (detailed description on the database search parameters and filters provided in Appendix A.1) together with a manual interpretation of MS and MS/MS spectra for verification of the SimLipid reported lipid ion species as:

- Mass-identification (Mass-ID): only mass-resolved lipid molecular ion species.
- Group-ID: lipid IDs at sum composition level with only class-specific head group identification (e.g., PC 32:0).
- PA-ID (partial-acyl identification): For example, PC 14:0*_18:0 where the suffix * indicates that the MS/MS spectrum does not feature characteristic ion/s corresponding to the fatty acid chain 14:0.
- FA-ID (lipid IDs with full-acyl identification) [32]: For example, PC 14:0_18:0, indicating that the MS/MS spectrum features all the characteristic ion/s corresponding to the head group/neutral loss of head group along with the fatty acid chains.

A detailed description of the process of manual verification and grading of the lipid ion species reported by SimLipid Database search along with example spectrum lists annotated with fragment ion species is provided (Appendix A.2). Although our goal of the study is to achieve in-depth profiling of triacylglycerols (TG), we have reported phosphatidylcholine (PC), phosphoethanolamine (PE), sphingomyelins (SM), sterol esters, cholesterol and derivatives (Cho Der), and diacylglycerols (DG) species that ionized well in positive mode of MS experiment.

We present the summary of the SimLipid MS/MS database search results in Table 1. SimLipid database search using LIPIDMAPS (LM) databases reported 1132 lipids ion species that consist of 9 Mass-ID, 25 Group-ID, 251 PA-ID, and 848 FA-ID (Table 2 for detailed breakdown). Of note, only the TG and DG lipid ions at the FA-ID level make it to the final list as an effort to retain only the highly confident IDs for our further studies (we removed 243/7 TG/DG species identified at PA-ID level from the final list, respectively). Hence, we have 882 non-redundant unique lipid species that we consider for further comparative and relative quantitative analysis.

The likelihood of MS/MS spectra not assigned with any possible lipid species by SimLipid software is high, because SimLipid relies on a database containing lipid species migrated from LIPIDMAPS (LM) (https://lipidmaps.org/) that does not have many of the glycerolipids with short-chain fatty acids, e.g., 2–8 carbons, that have been reported in the published literature on fatty acid profiles of bovine milks. Hence, those unassigned MS/MS spectra were manually interpreted for the identification and characterization of novel glycerolipids (Appendix A.3).

### 2.2. Characterization of Novel DG and TG Lipid Species that Are Not Yet Cataloged in the LIPIDMAPS Database

**Figure 1:**
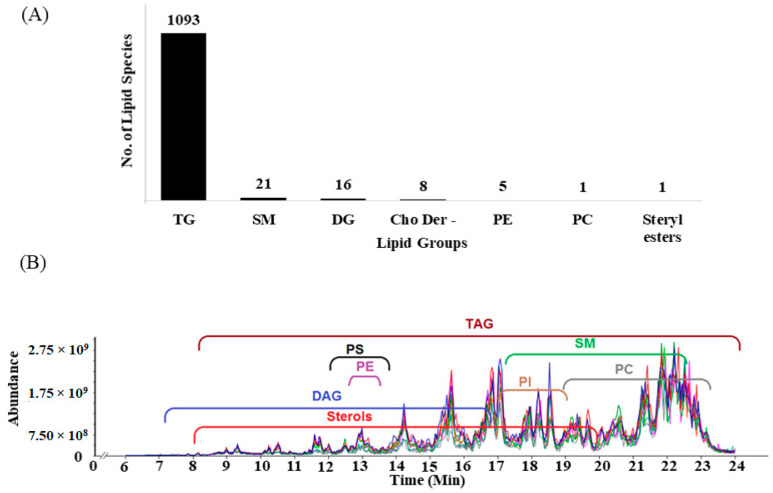
Different classes of lipids identified: (**A**) subclass-wise breakdown of the manually graded identifications (IDs); (**B**) Overlay of total ion chromatograms of all the runs annotated with the time periods in which lipid molecular ions from different classes were eluted in the LC domain.

The process of manually interpreting MS/MS spectra (Appendix A.3) resulted in the identification of 260 TG and one DG species that have not yet been cataloged by the LM database. Besides, we have reported nine phospholipids by manually investigating the presence of LC-compounds corresponding to ions of PC/PI/PS with their fatty acyl compositions made up of palmitic acid, stearic acid, and oleic acid between the replicates (detailed processed explained in the next section). Finally, we obtained a total of 1145 unique graded IDs comprising of 882 lipid species assigned by the initial SimLipid database search, 260 TG species and one DG species by manual interpretation of MS/MS spectra, and nine phospholipids detected using LC-compounds. Figure 1A shows the breakdown of the number of lipid molecular species belonging to each subclass. Figure 1B shows the overlay of total ion chromatograms (TIC) of all the runs annotated with the periods in which lipid ions from different classes eluted in the LC domain.

### 2.3. LC-MS Peak Detection for Assignment of Low Abundance PS, PI, and PC Lipid Molecular Ions

The MS/MS database search detected PE 34:1, PE 34:2, PE 36:1, PE 36:2, and PE 36:3 compositions. The PE compositions PE 36:1 (PE 18:0@_18:1) were detected at the PA-ID level while PE 36:2 was detected at the FA-ID level as PE 18:1_18:1, indicating the presence of oleic acid (18:1) at either Sn1 or Sn2 positions. For PC, only PC (34:1) as a group ID was detected. However, no PS and PI lipid molecular ions were detected from the MS/MS database search, possibly attributed to the fact that milk fat contains a very low concentration of phospholipids. Additionally, we employed MS (in +ve mode; note that TG lipids are not only present in high abundance in the sample, but also have high ionization efficiency) experiments with tandem MS acquired using a data-dependent acquisition (DDA) method that could not have acquired MS/MS data for very low abundant phospholipids. To facilitate quantitative analysis of these low abundant phospholipids, we investigated the presence of LC-compounds corresponding to ions of PC/PI/PS with their fatty acyl compositions made up of palmitic acid, stearic acid and oleic acid [31]. The following compositions: 32:0, 34:0, 34:1, 36:0, 36:1, and 36:2 of these phospholipid head groups were considered for LC-MS inspection (Appendix A.4).

### 2.4. Common Fatty Acid Chains of the Reported TG Species

The SimLipid MS/MS database search annotated 833 TG lipid species from the LIPIDMAPS database containing the following possible fatty acid chains—10:0, 12:0, 13:0, 14:0, 14:1, 15:0, 15:1, 16:0, 16:1, 17:0, 17:1, 17:2, 18:0, 18:1, 18:2, 19:1, 19:0, 20:0, 20:1, 18:3, 20:3, 21:0, 20:2, 22:0, 22:1, 20:4, 18:4, 22:4, 22:2, 22:5, 20:5, 22:3. Furthermore, 261 unique lipid species—1 DG and 260 TG lipid species —hat were not catalogued in the LM database, which we call as novel lipids, were identified by manually annotating *m/z* peaks (Supplementary Table S1). The lipid species identified included short chain fatty acyl TGs, for example, TG 2:0_4:0_18:1, TG 6:0_8:0_10:0, TG4:0_17:0_18:0, TG4:0_16:0_18:3, etc. The TG species that are not yet catalogued in the LM database contained the following possible acid chains—2:0, 3:0, 4:0, 5:0, 6:0, 7:0, 8:0, 9:0, 10:0, 10:1, 11:0, 12:0, 12:1, 13:0, 14:0, 14:1, 15:0, 15:1, 16:0, 16:1, 16:2, 17:0, 17:1, 18:0, 18:1, 18:2, 18:3, 19:0, 19:1, 20:0, 20:1, 20:2, 20:3, 20:4, 21:0, 22:0, 22:1, 22:5, 23:0, 23:1, 24:0, 24:1, 25:0, 26:0, 28:0, 30:0, which were manually annotated on the peaks of the MS/MS spectra. The MS/MS spectra were obtained of manually annotated 37 novel TG lipid species containing one or more above mentioned 46 possible fatty acid chains (Supplementary Figure S8, (1)–(37)). The compositions of TGs identified in our study were consistent with previous studies, of which the composition of 65 novel TGs were uniquely detected in our study, while two TGs—50:5 and 52:6—were not detected (Table 3). Furthermore, our study deciphers the most abundantly present TG lipids in accordance with our previous study [32] (Table 4).

### 2.5. Comparative Analysis of Lipids between Holstein Friesian Cow and Jaffarabadi Buffalo across Season

Bovine milk contains a diverse set of lipids that perform multiple functions and that have evolved due to sustained pressure on livestock to increase milk production by adopting and implementing appropriate animal husbandry practices. Nevertheless, there is limited information on the influence of endogenous (e.g., cow vs. buffalo) and exogenous (e.g., season) factors on change in the composition of lipid species.

**Figure 2:**
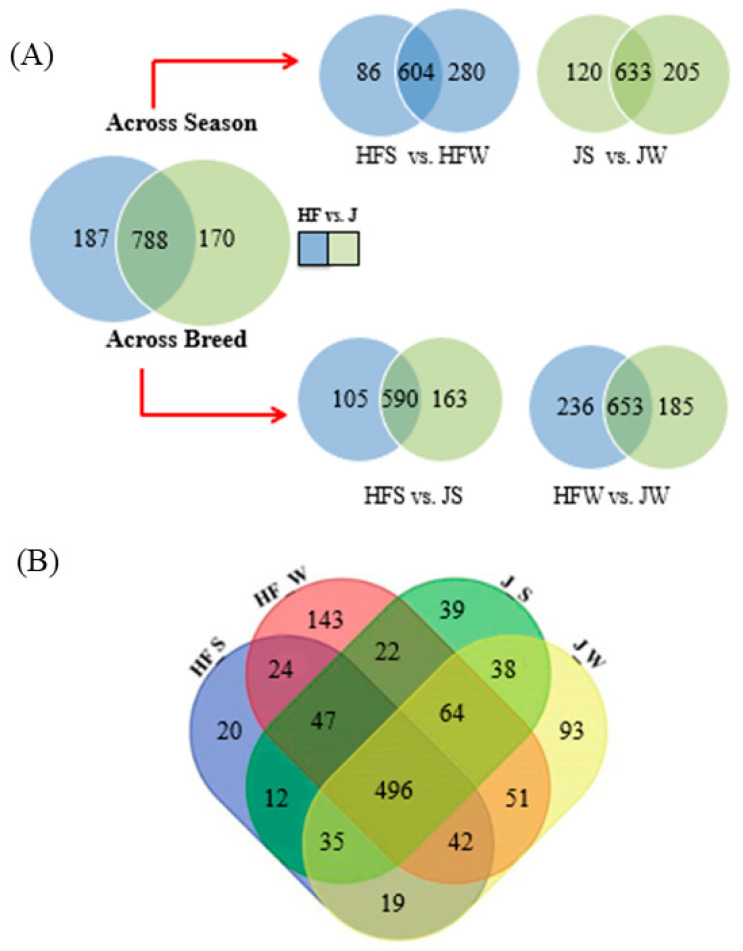
Venn diagram showing the overlap of identified lipids. (**A**) Overlap of the identified lipids between breeds across seasons (HFS vs. JS; HFW vs. JW), across breeds within seasons (HFS vs. HFW; JS vs. JW). (**B**) Overlap of the identified lipids in both animal groups across summer and winter season. HFS, Holstein Friesian summer; JS, Jaffarabadi summer; HFW, Holstein Friesian winter; JW, Jaffarabadi winter.

As a group, 1145 lipids graded IDs were identified in HF cow and J buffalo. Of these lipids, 187 and 170 were detected exclusively in each group, while the remaining 788 lipids were common to both animal groups (Figure 2A), although a lipid species may not be present in a particular season. A total of 496 lipid species were detected across breed as well as season. For instance, lipid species, such as Cho Der-[C33H58O6+Na]+, were detected only in the JW, but not detected in JS, HFS, and HFW; similarly, DG 12:0_16:0_0:0 and DG 16:0_20:1_0:0, DG 18:1_18:2_0:0 were identified in the winter season of HF and J, respectively. TG 2:0_6:0_20:1, TG 3:0_9:0_13:0 lipid species present in JW were found to be made up of short fatty acyl chain while long fatty acyl chains were present in JS, HFW. Extensive breed and season associated changes were observed in different lipid subclasses (e.g., triacylglycerols and phospholipids) in both animal groups. Additionally, new triacylglycerol lipid species have been reported for the first time by manually interpreting the MS/MS spectra and comparing the detected lipid species against a curated repository of published lipids and HFS. The variation and higher numbers of TG species were detected in the winter season compared to summer season, possibly due to availability and feeding of fresh seasonal grass and breed genetic factors [7]. Similarly, sphingomyelin (SM d43:1) was detected exclusively in the summer season. The lipid classes of TG, DG, PL, identified in HF cow were found to be consistent with the previous reports of [20], while lipid IDs from J buffalo have been reported for the first time.

It is interesting to note that 653 lipid species were common in both animal groups across the winter season, while 236 and 185 were exclusively detected in HF and J, respectively. Similarly, comparison across summer season in both groups resulted in the detection of 590 lipid species common in both groups with 105 and 163 species observed exclusively in HF and J, respectively. Similarly, multiple comparisons between animal groups across both seasons lead to the detection of 20 unique lipid species in HF in summer and 143 in winter, while 39 and 93 lipid species were observed in JS and JW, respectively (Figure 2B). In contrast, 496 lipid species were common to both groups across the summer and winter seasons. Interestingly, 38 unique lipids were found to be common between J winter and summer season while only 24 unique lipids (e.g., TG 12:0_14:1_14:1, TG 20:0_20:0_20:1, TG 15:1_15:1_19:0, SM d32:1, SM d38:2, SM d42:2) were common across seasons in HF. The identified lipids for HF and J are listed in (See Supplementary Table S2). Note that the cell value corresponding to a lipid species and a study group is the sum of the relative intensity of all the matched characteristic ions of the reported lipid species. For example, we have Cho Der-[C_33_H_54_O_6_+H]+ with 100 as the value in all the cells corresponding to HFS, HFW, JS, and JW because this lipid species, graded with Mass ID level, was identified with the base peaks in all the MS/MS spectra. Hence, cell value shall not be used as a means of relative quantitation of the lipid species between the experimental runs.

### 2.6. Variation in Unsaturation between Holstein Friesian Cow and Jaffarabadi Buffalo

The profile of unsaturated lipids obtained from this study followed the same trend that has been reported in HF. For example, C14:1, C16:1, C18:1, C18:2, C22:1 unsaturated lipids were observed in HF during the winter season. The pattern of increasing the unsaturation level in lipids during the winter season was consistent with previous studies of lipid profiling across the season in HF [35]. The DG and TG species were detected across both the seasons in HF, while phospholipids such as PE (e.g., 18:1_18:1) and PC (e.g., 34:1) were exclusively present in HF during the winter season. The observation of lipids exclusively in the winter season could be due to the nutritional availability of greener pasture and feed, which contribute to the availability of lipid precursors [33]. The results of our study showed a similar expression pattern to that observed for saturated and unsaturated fatty acids across both seasons, as reported previously in HF cow milk [36,37]. However, an important factor that has not been adequately controlled and is not clear is the influence of physiological and endocrinological factors on lactation stages and its correlation with the change in lipid composition. The majority of lipids identified in J during winter season were unsaturated lipid species (e.g., C14:1, C18:1, C18:2, C20:4) compared to the summer season. Additionally, phospholipid species like PC 34:1, PE 18:0_18:1 and PE 18:1_18:1 including cholesterol species such as Cho Der-[C_27_H_48_O_6_+Na]1+, Cho Der-20:0, Cho Der-22:0 were detected exclusively in winter, while GlcCer 34:2 was exclusively detected in the summer season. In the present study, higher numbers of saturated fatty acids (SFA) were detected during summer in both animal groups. However, trends in HF were found to be consistent with previous reports of the higher mean value of SFA during summer, and a higher level of unsaturation mean value was detected during the winter season [38], while it has been reported for the first time in J buffalo. The extent of unsaturation plays a vital role in the processing and physical properties of dairy products, for instance, the melting point and spread ability of fat are dependent on the acyl chain length and the degree of unsaturation [39,40].

One of the primary functions of milk lipids is to provide essential nutrients for the development of immune systems in newborns and protect against diseases. For example, a significant number of lipids of Omega family such as oleic acid (18:1), conjugated linoleic acid (18:2) and α-linolenic acid (18:3) previously reported with anti-diabetic and anticarcinogenic effects, arthritis, depression and Alzheimer’s disease [41] were confidently identified and constitutively expressed across summer and winter season in both animal groups.

Similarly, the milk fat C18:1 cis-9-to-C15:0 ratio previously known to plays a critical role as a discriminating factor in the diagnosis of hyperketonemia in dairy cows [42] was also constitutively expressed across summer and winter seasons in both groups. Additionally, inflammatory mediators such as oxylipids (e.g., 20-hydroxyeicosatetraenoic acid) derived from polyunsaturated fatty acids, including linoleic acid and arachidonic acid, a known marker of mastitis [43] were confidently identified in our study.

### 2.7. Relative Quantification of Lipids and Setting up of Hypothesis

#### 2.7.1. Collating Lipid Ion Species as Sum Compositions and Measuring Ion Abundances for Relative Quantitation

Many of the 1145 MS/MS identified lipid molecular species have the same sum composition or are structural isomers, i.e., lipid species that have same head-group as well as the same total number of carbons and double bonds on the fatty acyl chains. Since these structural isomers have the same observed parent ion mass, they can be grouped into 187 unique sum compositions or Group-IDs. For example, we identified seven structural isomers, namely TG 4:0_6:0_14:0, TG 4:0_8:0_12:0, TG 4:0_4:0_16:0, TG 4:0_10:0_10:0, TG 2:0_6:0_16:0, TG 2:0_4:0_18:0, and TG 6:0_8:0_10:0 from the MS/MS spectra for the lipid molecular composition TG 24:0, we call Group -ID (Supplementary Figure S9).

Of note, many of the lipid structural isomers belonging to a Group-ID or a sum composition comigrated in the LC domain [44,45], thereby posing a significant challenge in deconvoluting these peaks. Hence, we performed relative quantitation at the Group-ID level as done in the literature [32]. For each Group-ID, we also consider the sum of peak areas under-extracted ion chromatograms (XIC) belonging to all the molecular ion species of a Group-ID [46]. For example, the Group-ID TG 40:2 was quantified by summing the peak areas of prominent parent ion masses 656.5829 ([TG 40:2+NH4]^+^), and 661.5376 ([TG 40:2+Na]^+^) (see the XICs of these parent ion masses obtained using Thermo Fisher Scientific’s Xcalibur software shown in Supplementary Figure S10).

In addition to these 187 Group-IDs from MS/MS data analysis, we also have the nine phospholipids compositions, namely, PS(34:1), PS(36:2), PS(36:1), PI(34:1), PI(36:0), PI(36:2), PC(32:0), PC(34:2), and PC(36:2) that were manually detected using LC-MS peak detection method. All in all, we performed relative quantitation and statistical analysis for the 195 Group-IDs. Detailed software settings for generation of XICs are provided (Appendix A.5).

#### 2.7.2. Measurement of Ion Abundances of Analytes from Extracted Ion Chromatograms

The measured ion abundances of TG species showed high reproducibility across the replicates with average coefficient of variations (CV) 4.63%, 5.08%, 11.25%, and 10.27% respectively for the JW, JS, HFS, and KFW samples Supplementary Figure S11A. The other lipid species belonging to DG, PC, SM, PE, PI, PS, and Chol Der subclasses have average CVs 7.89%, 8.26%, 8.54%, and 9.41% for the JW, JS, HFS, and KFW samples (Supplementary Figure S11B). The two Group-IDs, namely, Steryl esters [C43H74O7+H] 1, Chol Der-[C27H44O6+H] 1+ have CVs > 30% in at least one of the samples and hence were removed from the relative quantitation analysis and further statistical analyses.

#### 2.7.3. Data Normalization and Data Transformation

The abundance of each lipid species was first normalized the measured against the total fat content of the milk. Subsequently, the relative ion abundance was calculated for each lipid molecular species as the ratio of the measured ion abundance of the molecular species to the cumulative sum of ion abundances of all the molecular species belonging to the same class. For example, the relative ion abundance of TG 25:0 species is the ratio of its measured peak area to the cumulative sum of the peak areas of all the 141 TG species. Finally, we use the log2 (log with base 2) of the ratio for analysis and visualization of fold changes describing the *n*-fold decrease of the ion abundance of an analyte to the total ion abundance of all the species belonging to the same class. Hence, all the fold change values are negative in our study.

#### 2.7.4. Two-Way Analysis of Variance Analysis (ANOVA)

A two-way ANOVA with replication for a “balanced design” was conducted—each subgroup has an equal number of observations—to investigate how the main effects (independent variables), namely breed and season and their interaction, affect the relative abundance of each analyte for the following hypotheses.

*Null Hypotheses:*

bH_0_: There is no difference in the average ion abundances of the analyte between the J and HF milk.

sH_0_: There is no difference in the average ion abundance of the analyte between the summer and winter.

iH_0_: There is no interaction effect.

*Alternative Hypotheses:*

bHa: There is a difference in the average ion abundances of the analyte between J and HF.

sHa: There is a difference in the average ion abundances of the analyte between summer and winter.

iHa: There is an interaction effect between season and breed type on average ion abundances of the analyte.

Detailed proofs for the verification of requisite assumptions of Two-Way ANOVA with Replicates are provided (Appendix A.6).

Finally, Tukey’s HSD (honestly significant difference) test was conducted as “post hoc analysis” for analytes that showed significant interaction effect to check if there is significant mean difference between the seasons of each breed (Appendix A.7).

### 2.8. Quantitative Comparison of TG Profiles between Holstein Friesian Cow and Jaffarabadi Buffalo

**Figure 3:**
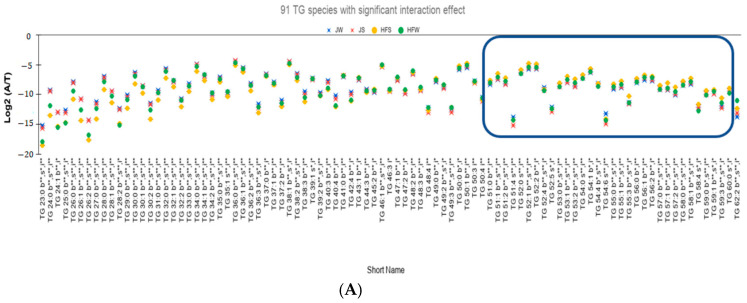

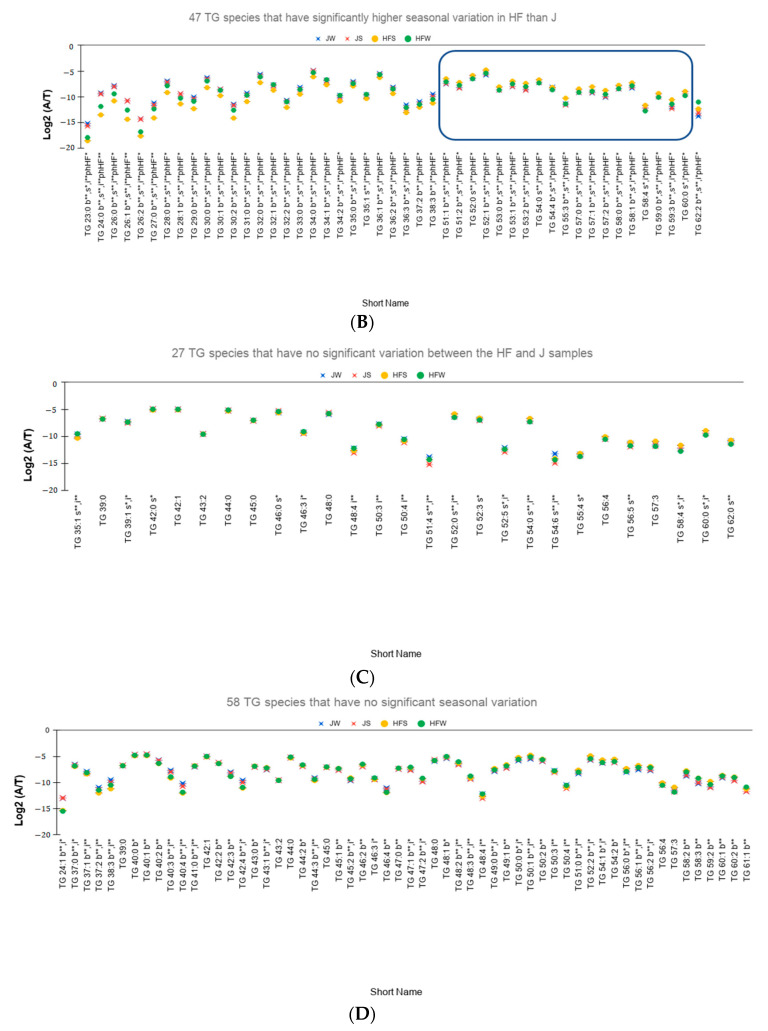

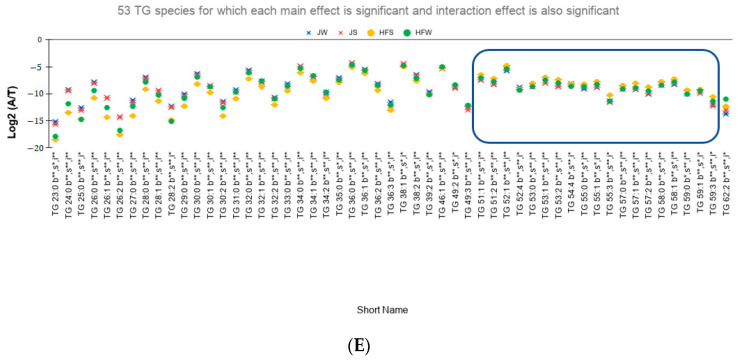
(**A**) Variation of the lipid profile of bovine milk: Each marker is the mean value of 15 J buffalos and 15 HF cows. Suffixes to the lipid composition: b, s, and i followed by ** (*p* < 0.01) and * (*p* < 0.05) as represent the statistical difference between the breeds, seasons, and interaction respectively. The reported *p*-values were calculated using Two-Way ANOVA with replication. A: normalized ion abundance of an analyte; T: total ion abundance of all the analytes belonging to the same subclass. 91 TG species showed statistically significant variations due to the interaction effect and at least one of the main effects. (**B**) 47 significant TG species with higher seasonal variation in HF samples than in J samples. The suffix phHF * and phHF ** represent Tukey’s HSD test *p*-value < 0.05 and *p*-value < 0.01 respectively for testing whether there is a significant variation between the seasons for the HF samples. (**C**) Only 27 TG species i.e., 19% of the reported TG species have statistically non-significant variation between the breeds. (**D**) 58 TG species do not vary significantly between the seasons. (**E**) 53 TG species have significant interaction effect as well as significant variations between the breeds and the seasons. TG species inside the rectangle (**A**,**B**,**E**) have higher relative abundances in summer season. Note that Standard Error for each group is used as the error bar.

We summarize the results of the Two-Way ANOVA with replication for balanced design for the 141 TG species as follows:

(i) Interaction effect is significant for 91 TG species (Figure 3A).
(ii) Out of the 91 species, 71 TG species have higher seasonal variation—defined as the absolute difference of relative abundances between the summer season and the winter season—for the HF samples than the J samples. On performing Tukey’s HSD test to check whether there is a significant variation between the seasons, 47 TG species have statistically significant seasonal variations (Figure 3B).
(iii) Twenty TG species have higher seasonal variation in J samples than in HF samples. However, only four TG species, namely TG 48:4, TG 50:4, TG 51:4, and TG 54:6, vary significantly based on Tukey’s HSD Test.

(a) The relative abundances of 81% of the total reported 114 TG species have significant variation between the breeds. Figure 3C showed the 27 TG species that do not have significant variation between the breeds.
(b) Relative abundances of 83 TG species, i.e., 59% vary significantly between the seasons. Figure 3D shows the relative abundances of the 58 TG species that do not vary significantly between the season.
(c) A total of 53 TG species have not only significant interaction effect but also significant main effects. Out of these 53 TG species, 48 TG species have higher seasonal variation in HF samples than the J samples of which 40 species are found to be statistically significant (Figure 3E). Only 5 TG species have higher seasonal variation in J samples than the HF samples. However, Tukey’s HSD test showed that none of the TG species is statistically significant.

Some observations from Figure 3E are as follows:

- Different effect of season on different breeds: J samples showed lesser seasonal variation than HF samples. On conducting Tukey’s HSD test for Post Hoc analysis of the 53 TG species with significant interaction effect, only one species, TG 23:0, has significant variation between the summer and winter seasons whereas for the HF samples, 40 TG species showed significant variation between the seasons.
- Potential seasonal effect based on the length of the fatty acids: For the HF milk samples, 34 TG species with TC—where TC is total number of carbons in Sn1, Sn2, and Sn3 chains—ranging from 23 to 49 have higher relative abundance in winter season than the summer season while 19 TG species with TC ranging between 51 to 59 have higher relative abundances in summer season than the winter season (rectangle in Figure 3B shows these 19 TG species). Of note, TG 62:2 has higher relative abundance in winter than in summer.

Similarly, a variation of the 11 DG (Figure S12A), 15 glycerophospholipids (Figure S12B), PC, PE, PS, and PI, 19 sphingomyelin (Figure S12C) and 7 sterols (Figure S12D) molecular groups were identified between the breeds.

**Figure 4:**
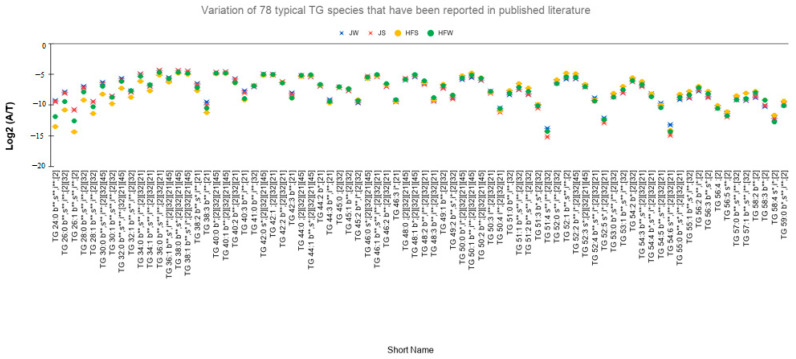
Comparison of TG lipids. Interbreed variation of TG compositions that have been reported in the published literature (*n* = 78, i*,** = 49, b*,** = 61, s*,** = 44, bsi*,** = 29, where *n* is the total number of TG species, b/s/i/bsi with suffix *,** denotes the number of TG species for which the effect of breed/season/interaction/each main effect as well as the interaction is significant).

Quantitative comparison of TG lipid profiles of bovine milk [21,32,47] was also performed between HF and J. Of the 78 lipid molecular species (Figure 4) reported in the recently published literature [21,32,34], 45 lipid molecular compositions showed significant variation in their relative ion abundances between the HF and J groups (On performing two-way ANOVA, **: *p*-value < 0.01, and **: *p*-value < 0.05, (*n* = 78, i*,** = 49, b*,** = 61, s*,** = 44, bsi*,** = 29 where *n* is the total number of TG species, b/s/i/bsi with suffix *,** denotes the number of TG species for which the effect of breed/season/interaction/each main effect as well as the interaction is significant). Each marker in Figure 4 is annotated with lipid composition with suffixes b/s/i followed by */** indicating statistical significance, then followed by reference number of the paper that reported the lipid species.

A previous study [6] reported the identification of 400 lipids from milk fat, while over 100 triacylglycerols (TG) groups were reported from HF cows [46], however, in our study 57 lipids in HF and 54 lipids in J did not overlap with the previously catalogued lipids, suggesting possible breed-specific differences. Taken together, our results significantly expand the number of identified lipids in the bovine milk and emphasize that it is essential to consider these normal changes before looking for useful lipids for incorporating into dairy foods to benefit human health and markers for disease diagnosis. For instance, odd fatty acids such as 15:0 and 17:0 have been reported as markers for dairy intake as plasma LDL-cholesterol has been related to the dietary intake of saturated fatty acids [48].

### 2.9. Multivariate Analysis of Lipid Profiles between Holstein Friesian Cow and Jaffarabadi Buffalo

Two datasets—(1) Quantitative TG profiles, only the 141 TG species, and (2) quantitative lipid profiles, all the 193 lipid species containing TG, DG, PC, PE, SM, PI, PS, and Chol Der—obtained above were subjected to unsupervised analysis with principal component analysis (PCA) and hierarchical cluster analysis (HCA) looking for natural clustering behavior due to the ion abundances of the lipid molecular species among the samples studied. The analysis was conducted using ClustVis software [49], an open source web tool developed by BIIT (a grouped between the Institute of Computer Science, University of Tartu, Tartu; competence center STACC, and Quretec https://biit.cs.ut.ee/), using the following settings—selected data transformation: none, and data scaling: Pareto scaling (mean-centered and divided by the square root of standard deviation of each variable). Note that, ClustVis software does not display loadings plot, and thus, MS excel chart functionality was used to generate the loadings plot.

**Figure 5:**
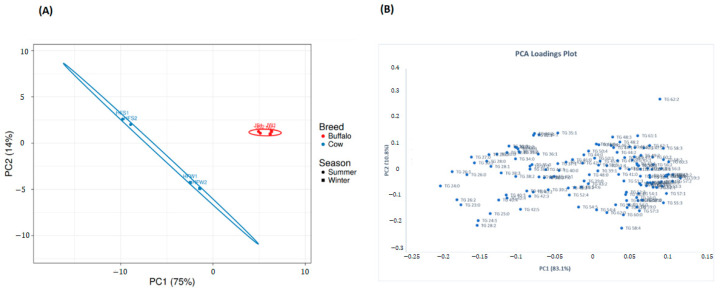

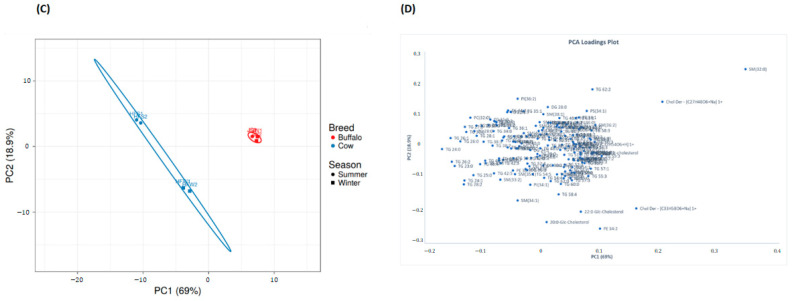
PCA scores and loadings plots. (A) Scores plot and (B) loadings plot from the PCA of the 141 TG species. (C) Scores plot and (D) loadings plot from the PCA of the 193 lipid species. The scores plot in (**A**) exhibits a wider separation between the JS and JW samples in the PC1-axis than in (**B**). Each dot/square is the average of 15 cows/buffalos. For each breed/season, samples were collected twice in the 1st lactation period between (**A**) 30–40 days for JS, (**B**) 50–60 days for JW, (**C**) 30–40 days for HFS, and (**D**) 50–60 days for HFW.

The first two principal components-PC1 and PC2–of the dataset 1 accounted for 89% of the explained variance. The scores plot (Figure 5A) and the corresponding loadings plot (Figure 5B) [50] show a clear separation between J samples and the HF samples. Furthermore, from the scores plot, we observed that HF samples between the summer and the winter seasons are separated farther apart in both the PC1 and PC2 axes than the J samples indicating higher seasonal variation in the quantitative TG profile of HF samples than the J samples. This observation is in line with our previous finding from Figure 3E that J samples showed lesser seasonal variation than HF samples.

PCA analysis of the dataset 2 also produced a similar result in terms of clear separation between the samples based on breed and season (scores plot and loadings plot shown in Figure 5C,D) respectively). However, PCA scores plot based on dataset 1, i.e., TG profiles (Figure 5A) provides a clearer separation between the J samples collected in summer and winter seasons than the separation between these samples in Figure 5D.

HCA was performed on both the datasets using the following settings: each row, i.e., quantitative profile of a lipid species is centered; Pareto scaling is applied to rows. Each column represents a sample of J/HF collected in a particular season. Both rows and columns are clustered using Euclidean distance and average linkage. Branches of the clustering trees are ordered with highest mean values first for easy visualization of the dendrograms.

**Figure 6:**
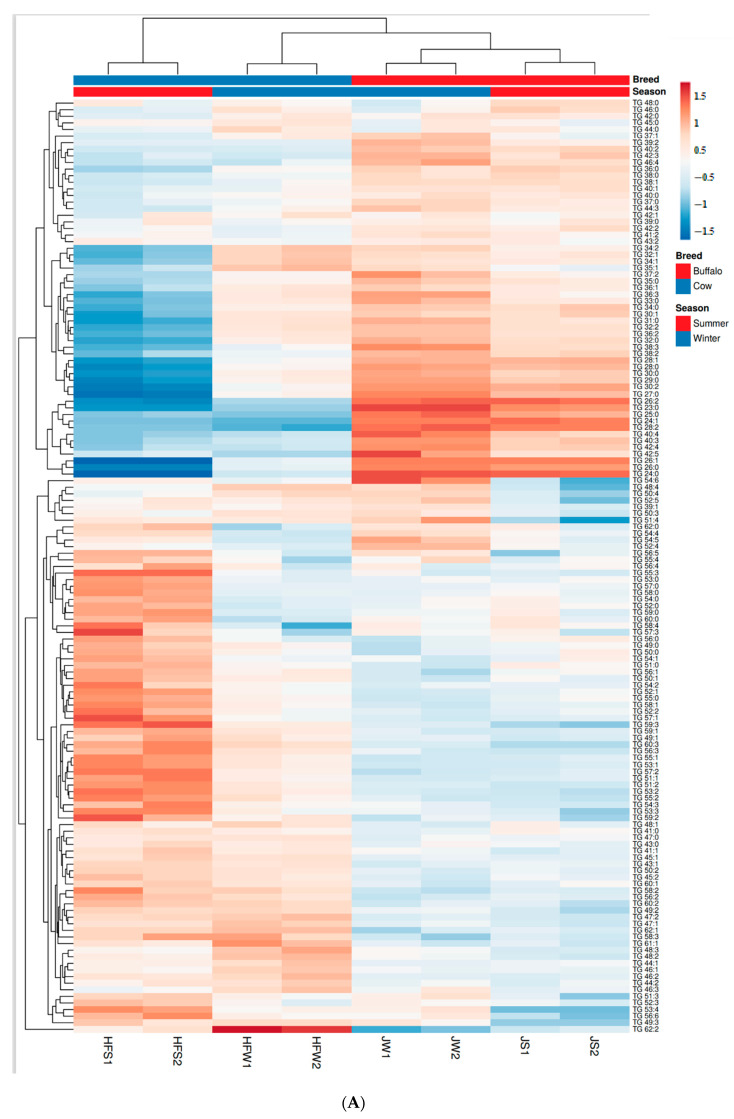

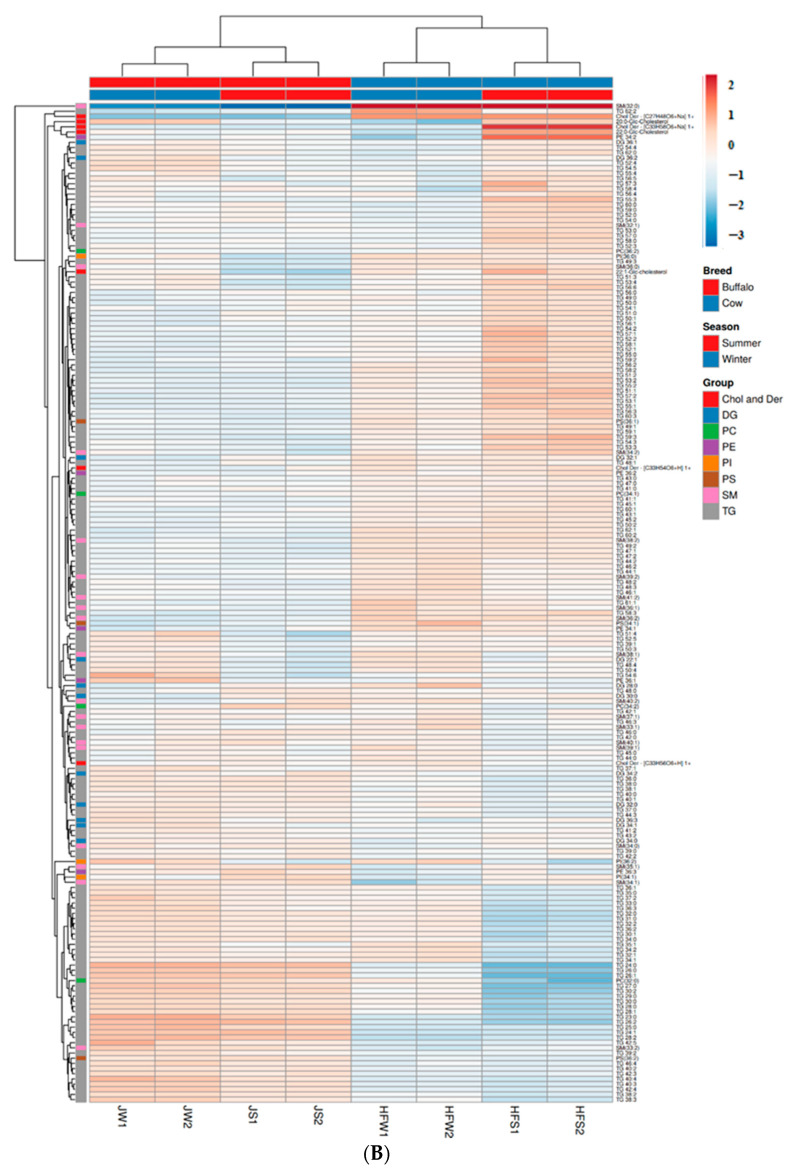
Clustering of samples. Dendrograms (columns) showing the similarity between the HF and J samples based on the (**A**) 141 TG species and (**B**) 193 lipid species. The row dendrograms on the Y-Axis show the clusters based on the lipid species. Corresponding heat maps showing the variation of molecular species between the samples are also displayed.

HCA of the columns on both the datasets (column dendrogram of Figure 6A,B) shows similar groupings were observed on PCA score plots—JS and JW samples are closer to each other than HFW and HFS samples—indicating lesser seasonal variation on the J samples. Figure 6A,B also shows the row clusters and the heat maps based on datasets 1 and 2 respectively.

## 3. Methods

### 3.1. Sample Collection

The Director of Research, Junagadh Agricultural University approved (protocol number is 18/1/2016/503) the collection of milk samples. A total of 60 animals, Jaffarabadi buffaloes (*n* = 30) and Holstein Friesian cows (*n* = 30) were used to collect milk samples for the study. In summer (April–May), milk samples were collected from 15 Jaffarabadi buffaloes and 15 Holstein Friesian cows post-calving, i.e., the first between 30–40 days while the second between 50–60 days. Similarly, in the winter season (December–January), milk samples were collected from 15 animals from each animal group between 30–40 days and 50–60-days post-calving. The average fat percentage (AFP) for J, and HF were 6.1%, and 3.02%, respectively. The milk fat content was measured by Gerber’s method using a butyrometer. Feeding of J buffalo comprised of green fodder, maize (*Zea mays*)*,* jawar (*Sorghum bicolor*), dry fodder and concentrate mixture like Amul-dan, Sabar-dan and Banas-dan, a balanced ration consisting of 20–22% protein and 6–7% oil in summer season, while fresh Jinjwa grass (*Dichanthium annulatum*) was fed in addition to the standard feed in winter season. Feed for Holstein Friesian consisted of doob grass (*Cynodon dactylon*), concentrate mixture (Amul-dan, Sabar-dan and Banas-dan), maize, jawar in summer season while Berseem (*Trifolium alexandrinum*) was added to the feed in winter season. The average milk yield of Jaffarabadi buffalo was 18–20 L/day while for HF cows yield is 10–15 L/day.

Raw milk samples were collected and stored at −20 °C and processed for lipid isolation. Thus, for each animal group, two study groups were formed: HFS and HFW for the HF cow, and JS and JW groups for the J buffalo. The suffixes S and W represent summer and winter seasons, respectively.

### 3.2. Isolation of Lipids from Milk Samples

Lipids were extracted from milk, as reported previously [24,51]. In brief, an equal volume of milk (20 µL) from each of the fifteen animals within first lactation stage was pooled (300 µL) and diluted by mixing with 800 µL of MS grade water. The diluted milk was mixed with 4 mL freshly prepared chloroform: methanol (CHCl_3_:MeOH) solution (2:1 ratio, *v*/*v*) in a 5 mL glass tube. The mixture was vortexed (Popular Traders, Ambala, India) for 10 min, followed by centrifugation at 2500× *g* for 10 min at 4 °C (Eppendorf, Hamburg, Germany) to facilitate phase separation. The lower organic phase was carefully transferred to a new glass tube while the upper aqueous phase was re-extracted for lipids by an additional 2 mL of the (CHCl_3_: MeOH) solution. The mixture was further vortexed and centrifuged at 2500× *g* for 10 min at 4 °C. The organic phase of chloroform/methanol was combined after recovery and dried in a liquid nitrogen stream. Schematic shows the workflow of the study (Supplementary Figure S1).

### 3.3. Ultra-Performance Liquid Chromatography Mass Spectrometry

The dried samples were resuspended in 500 µL of (CH_3_Cl: CH_3_OH 1:10) and further diluted at 1: 10 ratios for analysis. Lipids were separated by UPLC using an HPG-3400RS UPLC pump, autosampler, and column compartment system (Thermo Scientific, CA, USA). Samples (2.5 µL) in duplicates for each animal group were loaded onto a Hypersil Gold, a Q column (2.1 × 100 mm) containing 1.9 µ media (Thermo Scientific). Lipids were eluted using a complex gradient of H_2_O:CH_3_CN:IPA with A containing H_2_O:CH_3_CN (1:1, 0.1% formic acid, 5 mM NH_4_CHO_2_) and B containing CH_3_CN:IPA (1:9, 0.1% formic acid, 5 mM NH_4_CHO_2_). The gradient was: T = 0 min, 32% B, T = 1.5 min, 32% B T = 4 min, 45% B T = 5 min, 52% B T = 8 min, 58% B T = 11 min, 66% B T=14 min, 70% B T = 18 min, 70% B T = 21 min, 97% B T = 25 min, 97% B T = 25.1 min, 32% B T = 30 min, 32% at 260 µL/min over 30 min. The column oven was heated to 45 °C. Positive ions were generated by electrospray, and the Q Exactive mass spectrometer (Thermo Fisher Scientific, Bremen, Germany) operated in data-dependent acquisition mode (DDA). The heated electrospray source (HESI) was used with a high voltage of 3.8 kV applied; a vaporizer temp of 250 °C; sheath gas 20; aux gas 5 and the heated capillary set to T = 290 °C. A survey scan *m*/*z* 375–1800 was acquired (resolution = 70,000 at *m*/*z* 200, with an AGC target value of 3 × 10^6^ ions, max IT 250 ms) with lock mass enabled at *m*/*z* 445.12003. Up to the10 most abundant ions combining 2 micro scans (with a minimum AGC target of 5 × 10^4^, max IT 110 ms) were sequentially isolated (width *m*/*z* 1.8) and fragmented by stepped HCD (NCE = 20, 30, 45) with an AGC target of 2 × 10^5^ ions (resolution = 17,500 at *m*/*z* 200). The *m*/*z* ratios selected for MS/MS were dynamically excluded for 12 s, and charge state exclusion was not enabled.

### 3.4. Data Analysis Software Tools

#### 3.4.1. Tandem Mass Spectrometry Data Analysis for Lipid Identification

Automated annotation of lipid molecular ions using MS and MS/MS data was performed using SimLipid software (PREMIER Biosoft, Palo Alto, CA, USA). The annotated lipid ions were manually reviewed, verified, and graded based on the confidence level of identification achieved from the MS/MS data. We created a list of precursor *m/z* values of the MS/MS spectra that were not annotated with any lipid ion species and subjected it to “Bulk” Structure Searches—Search COMP_DB with a List of Precursor Ions [52] (for possible annotation of triacylglycerol (TG) ion species. The MS/MS spectra assigned with possible TG ion species were then subjected manual interpretation for possible identification of TG species that have not been catalogued in the LM database.

#### 3.4.2. Relative Quantification of Lipids and Statistical Analyses

Relative quantification of the identified lipid molecular ion species was performed using peak areas of extracted ion chromatograms. The extracted ion chromatograms were generated using SimLipid and Thermo Fisher Scientific’s Xcalibur software.

Data normalization, transformation, and statistical analyses pertaining to testing of hypotheses, and generation of charts were performed using Microsoft Excel in-built functions and user-defined functions. We conducted the test of normality of data including the Shapiro-Wilk test using online free tool [53]).

#### 3.4.3. Multivariate Analysis

ClustVis software [49] was used for performing unsupervised multivariate statistical analysis.

## 4. Conclusions

In summary, this is the first lipidomic analysis in a healthy cohort of HF cow and J buffalo across summer and winter seasons, resulting in the high confidence identification of 1145 unique lipids species, including 260 triacylglycerols and one diacylglycerol that have not been yet catalogued in LM database. The results of the comprehensive quantitative data analysis show breed and season associated changes not previously reported in bovine milk. Such changes reflect the dynamic nature of the complex milieu of milk and provide a foundation to improve our understanding to evaluate in future studies the effect of additional factors on lipid composition. The results of the present will help to facilitate the elucidation of the biological function of these lipids and their potential usage in infant formula and milk for the betterment of human health.

## Figures and Tables

**Table 1:** Summary of the SimLipid MS/MS database search identified using 15/10 ppm error tolerances for precursor and product ions and grading the lipid molecular ions based on observed diagnostic ions in the MS/MS spectra using MS excel.

**1**	**Total No. of MS/MS Spectra Subjected to SimLipid MS/MS Database Search**	**24,312**
2	No. of MS/MS spectra annotated with possible lipid molecular ions	6710
3	No. of unique graded IDs e.g., PC(16:0_18:1) or PC 34:1 From LIPIDMAPS	882
4	The number of unique DAG and TAG lipid species identified by manually annotating *m/z* peaks in the MS/MS spectra with neutral loss of all the possible fatty acyls for each lipid. LIPID MAPS has not yet catalogued these lipids species	261
5	No. of unique lipid compositions at Group-ID/Mass-ID levels for which relative quantitation was performed	196

**Table 2:** Breakdown of the 5/10 ppm LIPIDMAPS Search.

Group	Mass-ID	Group-ID	PA-ID	FA-ID	Class Wise Total Lipids
TG	N/C	N/C	243 *	833	1076
DG	N/C	N/C	7 *	14	21
PC	N/C	1	0	0	1
PE	N/C	3	1	0	4
Chol & Der	8 #	0	0	0	9
SM	0	21	0	0	21
Total					1132 − 243 * − 7 * = 882

*: PA-ID lipids that were not included in the final list after manually inspecting the MS/MS spectra; N/C: Not considered as a valid ID and removed from the result. #: The number of Chol and Der also includes 1 Steryl ester ID that was annotated at the Mass-ID level.

**Table 3:** List of triacylglycerol (TG) compositions previously reported in the published literature [2,23,33,34]. A total of 65 novel sum compositions have been reported. Only two lipid compositions, namely TG 50:5 and 52:6, were not detected in our method.

TG Lipid	Ours	[2]	[33]	[23]	[34]	TG Lipid	Ours	[2]	[33]	[23]	[34]	TG Lipid	Ours	[2]	[33]	[23]	[34]	TG Lipid	Ours	[2]	[33]	[23]	[34]
TG 23:0	1	0	0	0	0	TG 39:0	1	0	0	0	0	TG 48:1	1	1	1	1	1	TG 54:5	1	1	1	1	0
TG 24:0	1	1	0	0	0	TG 39:1	1	0	0	0	0	TG 48:2	1	1	1	1	0	TG 54:6	1	0	1	0	0
TG 24:1	1	0	0	0	0	TG 39:2	1	0	0	0	0	TG 48:3	1	1	1	1	0	TG 55:0	1	0	0	0	0
TG 25:0	1	0	0	0	0	TG 40:0	1	1	1	1	1	TG 48:4	1	0	0	0	0	TG 55:1	1	0	0	0	0
TG 26:0	1	1	1	0	0	TG 40:1	1	1	1	1	1	TG 49:0	1	0	0	0	0	TG 55:2	1	0	0	0	0
TG 26:1	1	1	0	0	0	TG 40:2	1	1	1	1	0	TG 49:1	1	1	1	0	0	TG 55:3	1	0	0	0	0
TG 26:2	1	0	0	0	0	TG 40:3	1	0	0	1	0	TG 49:2	1	1	1	0	0	TG 55:4	1	0	0	0	0
TG 27:0	1	0	0	0	0	TG 40:4	1	0	0	0	0	TG 49:3	1	0	0	0	0	TG 56:0	1	1	0	0	0
TG 28:0	1	1	1	0	0	TG 41:0	1	0	1	0	0	TG 50:0	1	1	1	1	1	TG 56:1	1	1	0	0	0
TG 28:1	1	1	1	0	0	TG 41:1	1	0	0	0	0	TG 50:1	1	1	1	1	1	TG 56:2	1	1	0	0	0
TG 28:2	1	0	0	0	0	TG 41:2	1	0	0	0	0	TG 50:2	1	1	1	1	1	TG 56:3	1	1	0	0	0
TG 29:0	1	0	0	0	0	TG 42:0	1	1	1	1	1	TG 50:3	1	1	1	1	0	TG 56:4	1	0	0	0	0
TG 30:0	1	1	1	0	1	TG 42:1	1	1	1	1	0	TG 50:4	1	1	1	1	0	TG 56:5	1	0	1	0	0
TG 30:1	1	1	1	0	0	TG 42:2	1	1	1	1	0	TG 50:5	0	0	1	0	0	TG 56:6	1	0	1	0	0
TG 30:2	1	0	0	0	0	TG 42:3	1	0	0	1	0	TG 51:0	1	1	1	0	0	TG 57:0	1	0	0	0	0
TG 31:0	1	0	0	0	0	TG 42:4	1	0	0	0	0	TG 51:1	1	1	1	0	0	TG 57:1	1	0	0	0	0
TG 32:0	1	0	1	1	1	TG 42:5	1	0	0	0	0	TG 51:2	1	1	1	0	0	TG 57:2	1	0	0	0	0
TG 32:1	1	1	1	0	0	TG 43:0	1	0	0	0	0	TG 51:3	1	1	1	0	0	TG 57:3	1	0	0	0	0
TG 32:2	1	0	0	0	0	TG 43:1	1	0	0	0	0	TG 51:4	1	1	1	0	0	TG 58:0	1	1	0	0	0
TG 33:0	1	0	0	0	0	TG 43:2	1	0	0	0	0	TG 52:0	1	1	0	0	0	TG 58:1	1	1	0	0	0
TG 34:0	1	1	1	1	1	TG 44:0	1	1	1	1	1	TG 52:1	1	1	1	0	1	TG 58:2	1	1	0	0	0
TG 34:1	1	1	1	1	0	TG 44:1	1	1	1	1	1	TG 52:2	1	1	1	1	1	TG 58:3	1	1	0	0	0
TG 34:2	1	0	0	0	0	TG 44:2	1	0	0	1	0	TG 52:3	1	1	1	1	0	TG 58:4	1	0	0	0	0
TG 35:0	1	0	0	0	0	TG 44:3	1	0	0	1	0	TG 52:4	1	1	1	1	0	TG 59:0	1	0	0	0	0
TG 35:1	1	0	0	0	0	TG 45:0	1	1	1	0	0	TG 52:5	1	1	1	0	0	TG 59:1	1	0	0	0	0
TG 36:0	1	1	1	1	1	TG 45:1	1	1	1	0	0	TG 52:6	0	0	1	0	0	TG 59:2	1	0	0	0	0
TG 36:1	1	1	1	1	1	TG 45:2	1	1	1	0	0	TG 53:0	1	0	0	0	0	TG 59:3	1	0	0	0	0
TG 36:2	1	0	0	0	0	TG 46:0	1	1	1	1	1	TG 53:1	1	0	0	0	0	TG 60:0	1	0	0	0	0
TG 36:3	1	0	0	0	0	TG 46:1	1	1	1	1	1	TG 53:2	1	0	0	0	0	TG 60:1	1	0	0	0	0
TG 37:0	1	0	0	0	0	TG 46:2	1	1	1	1	0	TG 53:3	1	0	0	0	0	TG 60:2	1	0	0	0	0
TG 37:1	1	0	0	0	0	TG 46:3	1	0	0	1	0	TG 53:4	1	0	0	0	0	TG 60:3	1	0	0	0	0
TG 37:2	1	0	0	0	0	TG 46:4	1	0	0	0	0	TG 54:0	1	1	1	0	0	TG 61:1	1	0	0	0	0
TG 38:0	1	1	1	1	1	TG 47:0	1	0	0	0	0	TG 54:1	1	1	1	1	0	TG 62:0	1	0	0	0	0
TG 38:1	1	1	1	1	1	TG 47:1	1	0	0	0	0	TG 54:2	1	1	1	1	0	TG 62:1	1	0	0	0	0
TG 38:2	1	0	0	1	0	TG 47:2	1	0	0	0	0	TG 54:3	1	1	1	1	0	TG 62:2	1	0	0	0	0
TG 38:3	1	0	0	1	0	TG 48:0	1	1	1	1	1	TG 54:4	1	1	1	1	0	**Total**	**141**	**64**	**60**	**42**	**21**

**Table 4:** The list of 21 lipid compositions and their content in the bovine milk adapted from that list of individual TG species [34]. The reported lipid compositions were detected in both the groups as the top 32 most abundant lipid compositions.

Rank (1 Being the Most Abundant)	TG Composition	Total Content (mol/100 mol)	Rank in J	Rank in HF
1	TG 36:0	7.2	1	4
2	TG 38:0	5	2	1
3	TG 34:0	4.8	6	17
4	TG 50:1	3.7	13	6
5	TG 48:1	2.9	10	8
6	TG 40:0	2.2	5	3
7	TG 38:1	2.2	3	5
8	TG 32:0	1.9	20	29
9	TG 52:2	1.8	17	13
10	TG 48:0	1.6	18	20
11	TG 40:1	1.4	4	2
12	TG 46:1	1.3	11	11
13	TG 46:0	1.3	14	15
14	TG 52:1	1.1	15	10
15	TG 44:0	1.1	9	14
16	TG 42:0	1.1	7	9
17	TG 36:1	1.1	16	22
18	TG 50:0	1	22	19
19	TG 30:0	0.9	27	32
20	TG 50:2	0.8	21	16
21	TG 44:1	0.7	12	12

## Supplementary Materials

The following are available online at https://www.mdpi.com/2218-1989/10/12/507/s1, Figure S1: Schematic of the workflow of the study. Figure S2: Typical SimLipid software graphical user interface (GUI) showing annotations of an MS/MS spectrum with a lipid ion that has been assigned Mass-ID using MS excel in-built functions. Figure S3: Typical SimLipid software GUI showing annotation of an MS/MS spectrum with a lipid ion that has been assigned Group-ID using MS excel in-built functions. Figure S4: Typical SimLipid software GUI showing annotation of an MS/MS spectrum with a lipid ion that has been assigned PA-ID using MS excel in-built functions. Figure S5: Typical SimLipid software GUI showing annotation of an MS/MS spectrum with a lipid ion that has been assigned FA-ID using MS excel in-built functions. Figure S6: Manual annotation of possible fatty acid chains of the [TG 23:0+NH4]+ ion using MS/MS data. Figure S7: XIC of [PS 36:1+H]+ ion and isotopic cluster with a monoisotopic peak at *m*/*z* 791.5597. Figure S8 (1): An example MS/MS spectrum of TG 2:0_4:0_18:0 that contains 2:0 as one of the fatty acids. Figure S8 (2): An example MS/MS spectrum of TG 3:0_16:0_18:1 that contains 3:0 as one of the fatty acids. Figure S8 (3): An example MS/MS spectrum of TG 4:0_6:0_14:0 that contains the fatty acids 4:0 and 14:0. Figure S8 (4): An example MS/MS spectrum of TG 4:0_5:0_16:0 that contains 5:0 as one of the fatty acids. Figure S8 (5): An example MS/MS spectrum of TG(4:0/6:0/14:0) that contains 6:0 as one of the fatty acids. Figure S8 (6): An example MS/MS spectrum of TG 5:0_7:0_15:0 that contains the fatty acids 7:0 and 15:0. Figure S8 (7): An example MS/MS spectrum of TG(4:0_8:0_12:0) that contains the fatty acids 8:0 and 12:0. Figure S8 (8): An example MS/MS spectrum of TG 9:0_14:0_18:0 that contains 9:0 as one of the fatty acids. Figure S8 (9): An example MS/MS spectrum of TG 10:0_12:0_14:0 that contains 10:0 as one of the fatty acids. Figure S8 (10): An example MS/MS spectrum of TG 4:0_10:1_14:0 that contains 10:1 as one of the fatty acids. Figure S8 (11): An example MS/MS spectrum of TG 11:0_14:0_16:0 that contains 11:0 as one of the fatty acids. Figure S8 (12): An example MS/MS spectrum of TG 6:0_12:1_14:1 that contains the fatty acids 12:1 and 14:1. Figure S8 (13): An example MS/MS spectrum of TG 8:0_10:0_13:0 that contains 13:0 as one of the fatty acids. Figure S8 (14): An example MS/MS spectrum of TG 4:0_15:1_16:0 that contains 15:1 as one of the fatty acids. Figure S8 (15): An example MS/MS spectrum of TG 6:0_10:0_16:2 that contains 16:2 as one of the fatty acids. Figure S8 (16): An example MS/MS spectrum of TG 4:0_16:0_17:0 that contains 17:0 as one of the fatty acids. Figure S8 (17): An example MS/MS spectrum of TG 4:0_14:0_17:1 that contains 17:1 as one of the fatty acids. Figure S8 (18): An example MS/MS spectrum of TG 4:0_15:0_18:2 that contains 18:2 as one of the fatty acids. Figure S8 (19): An example MS/MS spectrum of TG 4:0_16:0_18:3 that contains 18:3 as one of the fatty acids. Figure S8 (20): An example MS/MS spectrum of TG 4:0_16:0_19:0 that contains 19:0 as one of the fatty acids. Figure S8 (21): An example MS/MS spectrum of TG 4:0_18:0_19:1 that contains 19:1 as one of the fatty acids. Figure S8 (22): An example MS/MS spectrum of TG 4:0_16:0_20:0 that contains 20:0 as one of the fatty acids. Figure S8 (23): An example MS/MS spectrum of TG 16:0_20:1_26:0 that contains the fatty acids 20:1 and 26:0. Figure S8 (24): An example MS/MS spectrum of TG 8:0_14:0_20:2 that contains 20:2 as one of the fatty acids. Figure S8 (25): An example MS/MS spectrum of TG 4:0_18:0_20:3 that contains 20:3 as one of the fatty acids. Figure S8 (26): An example MS/MS spectrum of TG 4:0_16:0_20:4 that contains 20:4 as one of the fatty acids. Figure S8 (27): An example MS/MS spectrum of TG 4:0_16:0_21:0 that contains 21:0 as one of the fatty acids. Figure S8 (28): An example MS/MS spectrum of TG 4:0_14:0_22:0 that contains 22:0 as one of the fatty acids. Figure S8 (29): An example MS/MS spectrum of TG 16:0_22:1_24:0 that contains 24:0 as one of the fatty acids. Figure S8 (30): An example MS/MS spectrum of TG 14:0_16:0_22:5 that contains 22:5 as one of the fatty acids. Figure S8 (31): An example MS/MS spectrum of TG 19:0_19:1_23:0 that contains 23:0 as one of the fatty acids. Figure S8 (32): An example MS/MS spectrum of TG 18:0_18:1_23:1 that contains 23:1 as one of the fatty acids. Figure S8 (33): An example MS/MS spectrum of TG 16:0_22:1_24:0 that contains 24:0 as one of the fatty acids. Figure S8 (34): An example MS/MS spectrum of TG 18:1_18:1_24:1 that contains 24:1 as one of the fatty acids. Figure S8 (35): An example MS/MS spectrum of TG 18:1_18:1_25:0 that contains 25:0 as one of the fatty acids. Figure S8 (36): An example MS/MS spectrum of TG 16:0_18:1_28:0 that contains 28:0 as one of the fatty acids. Figure S8 (37): An example MS/MS spectrum of TG 16:0_16:0_30:0 that contains 30:0 as one of the fatty acids. Figure S9: MS2 spectra annotated with neutral loss of fatty acids from the parent ion of the lipid molecular species with Group-ID TG 20:4. Figure S10: A typical screenshot of Thermo Fisher Scientific’s Xcalibur software graphical user interface showing XICs of two molecular ions of the Group-ID TG 40:2 with parent ion masses 656.5829 ([TG 40:2+NH4]^+^), and 661.5376 ([TG 40:2+Na]^+^). The spectra displayed in the lower panel of the graphical user interface were manually stacked for easy data visualization. Figure S11 Coefficients of variation for (A): 141 TG species (B): 54 other lipid species, namely DG, PC, PE, PI, PS, and Chol Der at sum composition level in JW, JS, HFS, and HFW. Figure S12 Variation of the (A) 11 DG molecular groups, (B) 15 Glycerophospholipids–PC, PE, PS, and PI, (C) 19 sphingomyelin lipid compositions, and (D) 7 Sterols molecular groups between the breeds. Figure S13. (A) Data visualization of the JS group. (B) Data visualization of the JW group. (C) Data visualization of the HFS group. (D) Data visualization of the HFW group. Table S1: Novel lipid species that are not yet reported by the LIPIDMAPS database have been manually created based on the peaks observed in the MS/MS spectra. Table S2: List of Lipid IDs across Breeds and Seasons.

## Appendix A

### Appendix A.1. Automated Assignment of MS/MS Spectra with of Probable Lipid Species Using SimLipid Software Supplementary Material

Thermo Fisher Scientific’s Xcalibur ‘.raw’ files were directly read by SimLipid v.6.05 software for extraction of raw data from the LC-MS experimental runs and contained 41,576 spectra (17,264 MS1 spectra, and 24,312 MS/MS spectra). The MS/MS spectra were subjected to SimLipid MS/MS database searching using 5/10 ppm tolerances for the precursor/product ions. The database search was performed in non-targeted mode i.e., no filter based on category, class, and subclass of the lipid species was applied. However, for identification of lipid molecular ion species belonging to glycerolipids, glycerophospholipids, and sphingolipids categories using MS/MS data, the program was set to report only those lipid ions that have at least one diagnostic ion other than the parent ion species e.g., a phosphatidylcholine (PC) lipid molecular ion species will be included in the final result if the diagnostic ion of the head group at *m*/*z* 184.073 is observed in the MS/MS spectrum with its peak relative intensity higher than 50%. To avoid isotope overlapping of PC and Sphingomyelin(SM) lipid species, MS/MS spectra with dominant peak at *m*/*z* 184.073 with their precursor *m*/*z* corresponding to the M+0 peak of the isotopic cluster in the MS1 spectra are only considered for the MS/MS analysis. Similarly, PE lipids were considered only if the MS/MS spectra feature peak at *m*/*z* corresponding to neutral loss of 141.0191 from the parent ion. SimLipid database searches annotated 6710 MS/MS spectra with possible lipids ions belonging to subclasses of triacylglycerol (TG), diacylglycerol (DG), phosphatidylethanolamine (PE), cholesterol derivatives (Cho Der), sterols esters, phosphatidylcholine (PC), and Sphingomyelin (SM) from out of the total 24,312 spectra between experimental runs. It must be noted that these annotated lipid ions contain redundant lipid species and need to be verified based on observed characteristic ions in the MS/MS spectra for quality assurance as described in the following sections.

### Appendix A.2. Verification and Grading of the Lipid Ion Species Reported by SimLipid Database Search

The SimLipid database contains lipid structures and associated information migrated from LIPID MAPS (LM) database (https://www.lipidmaps.org/). The program follows the same naming convention e.g., TG(12:0/12:0/18:4(6Z,9Z,12Z,15Z))[iso3] bearing the LM ID LMGL03012642, that contains detailed information on the Sn1/Sn2/Sn3 positions, double bond positions in the FA chains and cis-trans isomerism. However, peaks observed in MS/MS spectra may not provide enough data to distinguish these isomers leading to difficulty in reporting the identified lipid ions with confidence. Consequently, as a solution, we have implemented Microsoft (MS) Excel in-built formulas within the SimLipid report-an MS Excel format file containing a list of identified lipid molecular ions along with their corresponding MS/MS spectra list annotated with matched diagnostic ions—to classify the identified lipid molecular ion into four different grades based on:

- Mass-ID (only mass-resolved lipid molecular ion species);
- Group-ID (lipid IDs at sum composition level with only class-specific head group identification);
- PA-ID (lipid IDs with Partial-Acyl Identification);
- FA-ID (lipid IDs with Full-Acyl Identification) [32].

Detailed explanation on the process of grading lipid IDs based on characteristic ions observed in the MS/MS spectra is described as follows:

- Mass-ID (only mass-resolved lipid molecular ion species): This is the case when a lipid molecular ion species is assigned to an MS/MS spectrum because its precursor *m/z* value is within a specified mass tolerance e.g., 5 ppm and MS/MS spectrum features ions corresponding to the parent ion mass e.g., [M+NH_4_]^+^, [M+H]^+^. Since no peak corresponding to any diagnostic ion corresponding to class-specific head groups or FA chains was observed, we use a naming convention that includes the subclass name with the formula of the lipid molecular ion. The MS/MS spectrum (See Supplementary Figure S2) with precursor *m/z* 549.4128 featuring peaks at *m/z* 549.4123 (relative intensity (RI): 100%) was annotated with the lipid species bearing LM ID LMST01010173 belonging to lipid class: Sterols, subclass: Cholesterol and derivatives (Cho Der). We assign a Mass-ID Cho Der-[C33H56O6+H] 1+ (See Supplementary Figure S2). Of note, this assignment was purely on the exact mass database search. There was no diagnostic ion corresponding to the Cholesterol e.g., peak at *m/z* 369.352. Nevertheless, we still use the Cho Der- because LMST01010173 is the lipid molecular ion species that has the minimum mass deviation (3.5326 ppm) from the observed precursor *m/z* value in the SimLipid database.
- Group-ID (lipid IDs with only class-specific head group identification): We assign Group-ID for an MS/MS spectrum that features peaks corresponding to diagnostic ions of class-specific head groups e.g., peak at *m/z* 184.073 for lipids with phosphocholine head group. However, the MS/MS spectrum does not have peaks that facilitate the identification of fatty acyl composition of the lipid species. For example, for an MS/MS spectrum with precursor *m/z* 760.5854 and product ions at *m/z* 86.0969(RI: 20%), *m/z* 104.1075(RI: 2%), and *m/z* 184.0736(RI: 100%), SimLipid annotates the lipid species bearing LM ID LMGP01010005, Common Name: PC (16:0/18:1(9Z)). Additionally, SimLipid also reports “Name: PC (16:0_18:1)” and “Short Name: PC (34:1)”. Since there was no observed peak that corresponds to the diagnostic ion of either of the probable fatty acid chains reported by SimLipid, we assign PC (34:1) (See Supplementary Figure S3). Furthermore, label-free quantitative analysis of low abundant phospholipids was performed.
- PA-ID (lipid IDs with Partial-Acyl Identification): In this case, not all the FA chains in a lipid species were identified by peaks in the MS/MS spectrum. For example, SimLipid annotated an MS/MS spectrum (See Supplementary Figure S4) with precursor *m/z* 782.723 at 21.9502 min with a ammonium adducted TG lipid molecular ion that has the Common Name: TG(13:0/14:0/18:0), LM ID: LMGL03013680, Name: TG(13:0_14:0_18:0), Short name: TG(45:0). Peaks observed in the MS/MS spectrum were annotated by SimLipid as follows (Fragment name Observed *m/z*(RI)): *m/z* M-18:0-NH_3__481.4257(RI: 95.3%), M-14:0-NH3_537.4888(RI: 8.3%). The diagnostic ions corresponding to the FA chains 14:0 and 18:0 were observed in the spectrum. However, there was no ion corresponding to 13:0. Hence, we assign a graded ID TG 13:0@_14:0_18:0; the suffix @ indicating that the diagnostic ion of this FA chain was not observed in the spectrum.
- If the MS/MS spectrum features only the peaks corresponding to diagnostic ions of one of the three FA chains of the TG lipid molecular species e.g., only the peak at *m/z* 481.4257, then we use the Group-ID, TG 45:0, as the graded ID instead of using the notation TG 13:0@_14:0@_18:0 (See Supplementary Figure S4).
- FA-ID (lipid IDs with Full-Acyl Identification) [54]: In this case, an MS/MS spectrum features *m/z* peaks corresponding to all the FA chains. For example, SimLipid assigns lipid species with the Common Name: TG (16:0/18:0/18:1(9Z)), Name: TG (16:0_18:0_18:1), Short Name: TG (52:1), LM ID: LMGL03010085 for the MS/MS spectrum with precursor *m/z* 878.819 at 22.5484 min and product ions annotated as follows (Fragment name Observed *m/z* (RI)): 15:0 C=O+_239.2374(4.4786), 17:1 C=O+_265.2541(8.0049), 17:0 C=O+_267.2693(1.3489), M-18:0_577.5211(75.1463), M-18:1_579.5364(84.6283), M-16:0_605.5515(59.7802). We use the graded ID TG 16:0_18:0_18:1 for analysis (See Supplementary Figure S5).

The SimLipid MS/MS database search result containing 6710 MS/MS spectra annotated with lipid molecular ions were graded using the MS Excel in-built formulas. It must be noted that as an effort to reduce false positives of lipid IDs, we considered only the DG and TG lipid ions that were identified at the FA-ID level. Similarly, only phospholipids that were identified at the Group-ID level were considered. However, for Cho Der lipid species, we considered lipid species annotated at Mass-ID level too because many sterols lipid species generate only parent ion (and neutral loss of H_2_O from it) as fragment ions in their MS/MS spectra (e.g., https://www.lipidmaps.org/data/standards/fetch_gif_mult.php?&MASS=385&LM_ID=LMST01010066&TRACK_ID=156).

### Appendix A.3. Characterization of Novel DG and TG Lipid Species that Are Not Yet Cataloged in the LIPIDMAPS Database

The initial MS/MS database search of the raw data using SimLipid software that contains lipid species migrated from the LM database does not annotate many TG lipid species that have been commonly reported in the published literature or some of the unsaturated forms [2,23,34,46,47,55]. Furthermore, the database search results also missed out on the majority of the TG lipid species containing the majority of the reported fatty acyls that have been successfully detected and measured using different separation techniques in the published literature [28,56,57]. Consequently, a stepwise procedure was adopted to manually identify additional TG species. Step wise procedure for characterization of novel DG and TG lipid species that are not yet cataloged in the LIPIDMAPS database is described as follows.

Step 1. Assignment of TG Composition on Each MS/MS spectrum. A mass list containing all the observed precursor *m/z* values of all the MS/MS spectra from the raw data files was subjected to “Bulk” Structure Searches—Search COMP_DB with a List of Precursor Ions (http://www.lipidmaps.org/resources/tools/bulk_structure_searches.php?database=COMP_DB) using the following parameters—Ion adduct: [M+NH4]+; Specify Mass Tolerance (+/− *m/z*): 0.005 *m/z*, Specify Chains: All chains, Optionally restrict search by lipid category/class: Tri(acyl|alkyl)glycerols (TG). All the trialkylglycerols are removed from the results.

Step 2. Annotation of Possible Fatty Acid Chains using Product Ions Data. For the annotated TG composition on an MS/MS spectrum, all the *m/z* values of the observed peaks were examined for matched against neutral loss of FA chains. For example, an MS/MS spectrum at retention time 8.57 min (raw data file of the pooled sample of 15 J buffaloes). The precursor *m/z* value 474.3788 is assigned [TG 23:0 + NH4]+ ion with a mass deviation of 0.33 ppm. On examining the observed *m/z* values corresponding to major peaks of the MS/MS spectrum, fatty acid chains were detected.

(i) The observed peak (Supplementary Figure S6) at *m/z* 215.1280 indicating the neutral loss of 15:0 from the [TG 23:0 + NH4]+ ion, i.e., 474.3788 − 215.1280 − 18.033823 = 241.217 which matches the theoretical mass of 15:0 with 0.9 (approx.) ppm mass deviation.
(ii) Similarly, the observed peak at *m/z* 369.3001 indicates the neutral loss of the fatty acid chain 4:0 from the [TG 23:0 + NH4]+ ion (Supplementary Figure S6).
(iii) Finally, TG 23:0 could have the possible lipid species TG 4:0_4:0_15:0 (we use the underscore, “_”, between the fatty acid chains to indicate non-specificity of the Sn1/Sn2/Sn3 positions of these reported fatty acid chains).
(iv) Once we establish the probable lipid species for an MS/MS spectrum, we create structures using LM and create a glycerolipid structure from LM Abbreviation”.
(v) (http://www.lipidmaps.org/tools/structuredrawing/StrDraw.pl?Mode=SetupGLStrDraw) wherein we enter the lipid species abbreviation and selecting the option of “Allow arbitrary chain abbreviation” as YES.
(vi) The generated SDF files are imported into the SimLipid server database for automated assignment of lipid species on the MS/MS spectra across all the raw data files.
(vii) The process (i)–(v) is repeated for all the MS/MS spectra with annotated TG compositions.

### Appendix A.4. LC-MS Peak Detection for Assignment of Low Abundance PS, PI, and PC Lipid Molecular Ions

We accept an ion as a valid LC compound provided:

(i) If it has an LC-peak with a minimum peak width of 0.1 min when plotted the XIC with 0.005 Da mass tolerance.
(ii) The averaged spectrum of all the MS1 data across the start and end time of the XIC features isotopic cluster with the ion as the monoisotopic peak.

We finally obtained valid LC-compounds of the following 9 lipid compositions PS(34:1), PS(36:2), PS(36:1) (See Supplementary Figure S7), PI(34:1), PI(36:0), PI(36:2), PC(32:0), PC(34:2), and PC(36:2) and included them in the relative quantitation as well as multivariate analysis.

### Appendix A.5. Measurement of Ion Abundances of Analytes from Extracted Ion Chromatograms

We used peak areas of the 195 unique Group IDs from the raw data files corresponding to the experimental LC-MS runs extracted as follows. We created a preferred mass list containing the theoretical *m*/*z* values (we simply call it “mass”) of the 195 unique graded IDs and XIC of each mass was generated using SimLipid with the following parameter values–mass tolerance for generating XIC: (+/−) 0.005 *m*/*z*; minimum time in the LC-domain for a valid LC-compound: 0.1 min; Minimum Peak Height: 200,000; Smooth the XIC with Savitzky-Golay 5 points. The peak list containing all the masses with peak areas was exported and aligned across the experimental runs into MS Excel file and sum the peak areas belonging to lipid molecular species with the same Group-ID. In case of missing peaks—this happens when software peak detection algorithm did not report a peak area corresponding to a mass of an analyte due to non-conformation of the parametric conditions for a valid peak—ion abundances of the mass from raw data files were again extracted using the mass tolerance, peak width, retention times as applicable and then finally smoothed the data Savitzky-Golay 5 points algorithm.

### Appendix A.6. Verification of the Assumptions of the Two-Way ANOVA

We verified the following requisite assumptions by subjecting the data into various statistical analyses.

- Measurement of the dependent variable at the continuous level: This condition is satisfied.
- Two or more than two categorical independent groups in two factors: This condition is satisfied with our goal to test the variation in average relative ion abundances of analytes between different breeds and seasons.
- Categorical independent groups should have the same size: Our data satisfy this condition.
- Independence of observations: Our data were collected independently.
- Normal distribution of the population from which each sample is drawn.
- Homoscedasticity or Homogeneity of the variance.

#### Appendix A.6.1. Test of Normality of the Population from Which the Data Is Drawn

We proved the normality of the data using the Shapiro-Wilk test, using a right-tailed normal distribution for each combination of the groups of the two independent variables, namely JW, JS, HFW, and HFS.

The Shapiro-Wilk test tests the null hypothesis

H_0_: a sample *x*_1_, …, *x_n_* came from a normally distributed population.

Against the alternative hypothesis

Ha: a sample *x*_1_, …, *x_n_* came from some distribution other than Normal.

The test statistic is:

(A1) $$W=\frac{\sum_{i=1}^{n}{\left(a_{i}x_{\left(i\right)}\right)}^{2}}{\sum_{i=1}^{n}{\left(x_{i}-\bar{x}\right)}^{2}}$$

where *x_(i)_* is the *i*th order statistics and $\bar{x}$ is the sample mean. The coefficients *a_i_* are defined in Shapiro and Wilk (1965) [58].

On subjecting the data from the JW group to the Shapiro-Wilk test, using a right-tailed normal distribution, we obtained the *p*-values = 0.461567, and hence accept the null hypothesis. Similarly, we obtained the following *p*-values 0.645503, 0.282420, and 0.508126 for the study groups JS, HFS, and HFW, respectively, and hence accept the null hypotheses.

We also have provided the Quantile-Quantile (QQ) plots, and the histograms of each group showing normally distributed data (See Supplementary Figure S13).

The *p*-values, and charts were generated using the online tool (Ref. [53] that can be directly generated using R codes too).

#### Appendix A.6.2. Homoscedasticity or Homogeneity of the Variance

We verified the requisite assumption for homogeneity of variances of the four groups by conducting the Levene’s Test [59,60] a detailed explanation of the test with work out example is available at https://www.itl.nist.gov/div898/handbook/eda/section3/eda35a.htm. We chose Levene’s Test for verification of the homogeneity of the sample variances because we have already verified that our data follows a normal distribution.

The Levene’s test is defined as follows. Let *s_i_*^2^, *i* = 1, 2, 3, …, *k* denotes the sample variance of the *i*th sample, then we test the following null hypothesis

H_0_: *s*_1_^2^= *s*_2_^2^= … = *s_i_*^2^ = … = *s_k_*^2^.

Against the alternative

H_a_: *s_i_*^2^ ≠ *s_j_*^2^ for at least a pair (*i*, *j*).

Given a variable Xij, jth observation of *i*th sample with sample of size *N* divided into *k* subgroups, where *Ni* is the sample size of the *i*th subgroup, the Levene’s test statistic is defined as:

(A2) $$W=\frac{\left(N-K\right)\sum_{i=1}^{k}Ni{\left(\bar{Zi}.-\bar{Z..}\right)}^{2}}{\left(k-1\right)\left(\sum_{i=1}^{k}\sum_{j=1}^{Ni}{\left(\mathrm{Zij}-\bar{Zi}.\right)}^{2}\right)}$$

where Zij can be one of the following definitions:

1. Zij = |Xij-$\bar{Xi}.$| where $\bar{Xi}.$ is the mean of the *i*th sample. This choice provides the best power for symmetric, moderate-tailed, distributions.
2. Zij = |Xij-Median(i.)| where Median(i.) is the median of the *i*th sample. The choice of median performed best when the underlying data followed a skewed distribution.
3. Zij = |Xij-trimMean(i.)| where trimMean(i.) is the 10% trimmed mean of the *i*th sample. The choice of using the trimmed mean performed best when the underlying data followed a Cauchy distribution (i.e., heavy-tailed).

$\bar{Zi}.$ are the group means of the Zij and $\bar{Z..}$ is the overall mean of the Zij. The test statistics W follows F distribution with k-1, and N-k degrees of freedom.

Since we have already proved that the data follows Normal distribution, the test boils down to One-Way ANOVA of the definition 1 of the Zij. We use MS excel’s Data Analysis module to calculate the ANOVA.

**Table A1:** ANOVA: Single Factor for Mean with α= 0.05.

**SUMMARY**						
**Groups**	**Count**	**Sum**	**Average**	**Variance**		
JW difference	195	503.1354	2.580182	3.279944		
JS Difference	195	519.703	2.665144	3.756559		
HFS Difference	195	554.1781	2.841939	4.800262		
HFW Difference	195	540.3826	2.771193	4.352645		
**ANOVA**						
**Source of Variation**	**SS**	**df**	**MS**	***F***	***p*-Value**	**F Crit**
Between Groups	7.78679	3	2.595597	0.641307	0.588588	2.616378
Within Groups	3140.746	776	4.047352			
Total	3148.532	779				

The *p*-value of the mean-based *F* = 0.6413 with degrees of freedoms 3, and 776 is 0.5886. This accepts the null hypothesis that the all the variances of all the four groups are equal.

### Appendix A.7. Tukey’s HSD (Honestly Significant Difference)

The Tukey’s Honest Significant Difference test [61] is a post-hoc test based on the studentized range distribution. This is typically performed after we have run an ANOVA and found significant results, however, an ANOVA test doesn’t exactly reveal which sample means are different. Tukey’s HSD to find out which specific group’s means (compared with each other) are different. The test compares all possible pairs of means with the following null hypotheses:

Ho: All means being compared are from the same population (i.e., μ1 = μ2 = μ3 = … = μk),

Assumptions for the test:

- Observations are independent within and among groups.
- The groups for each mean in the test are normally distributed.
- There is equal within-group variance across the groups associated with each mean in the test (homogeneity of variance).

We have already proved that our data satisfies all the basic assumptions.

To test all pairwise comparisons among means using the Tukey HSD, calculate HSD for each pair of means using the following formula:

(A3) $$\mathrm{qHSD}=\frac{Mi-Mj}{S\sqrt{2/n}}$$

where:

- *Mi* − *Mj* is the difference between the pair of means with *Mi* > *Mj*.
- *S*2 is the pooled sample variance is the Mean Square Within, and *n* is the number in the group or treatment.
