# Supplementary material for: Comparative Analysis of Milk Triglycerides Profile between Jaffarabadi Buffalo and Holstein Friesian Cow
Source: Metabolites. 2020 Dec 11;10(12):507. doi: 10.3390/metabo10120507 (PMC7764747; doi:10.3390/metabo10120507)
Supplement: Supplementary file 1 [file metabolites-10-00507-s001.pdf]

Figure S1: Schematic of the workflow of the study.

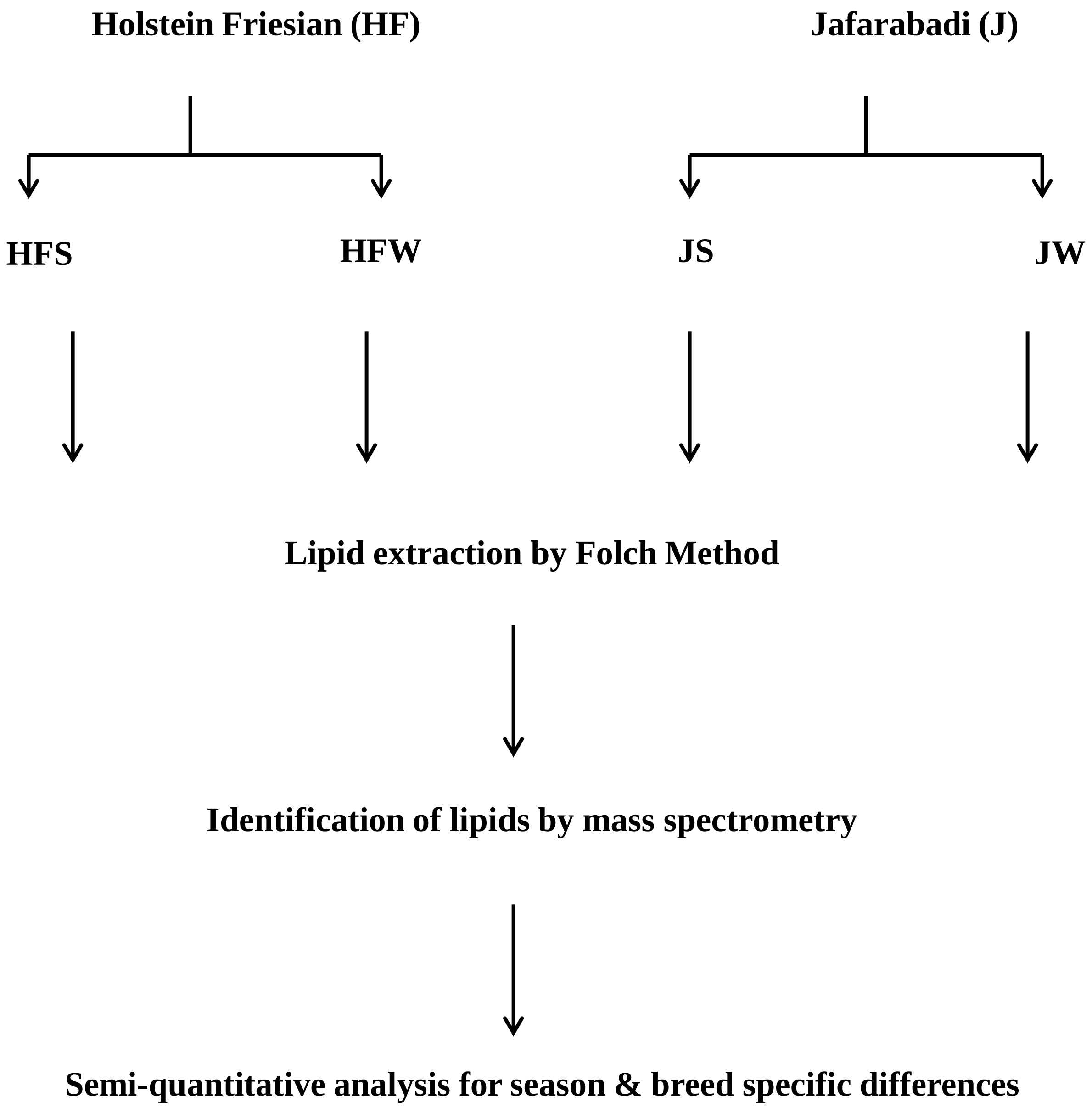

Figure S2: Typical SimLipid software graphical user interface (GUI) showing annotations of an MS/MS spectrum with a lipid ion that has been assigned Mass-ID using MS excel in-built functions.

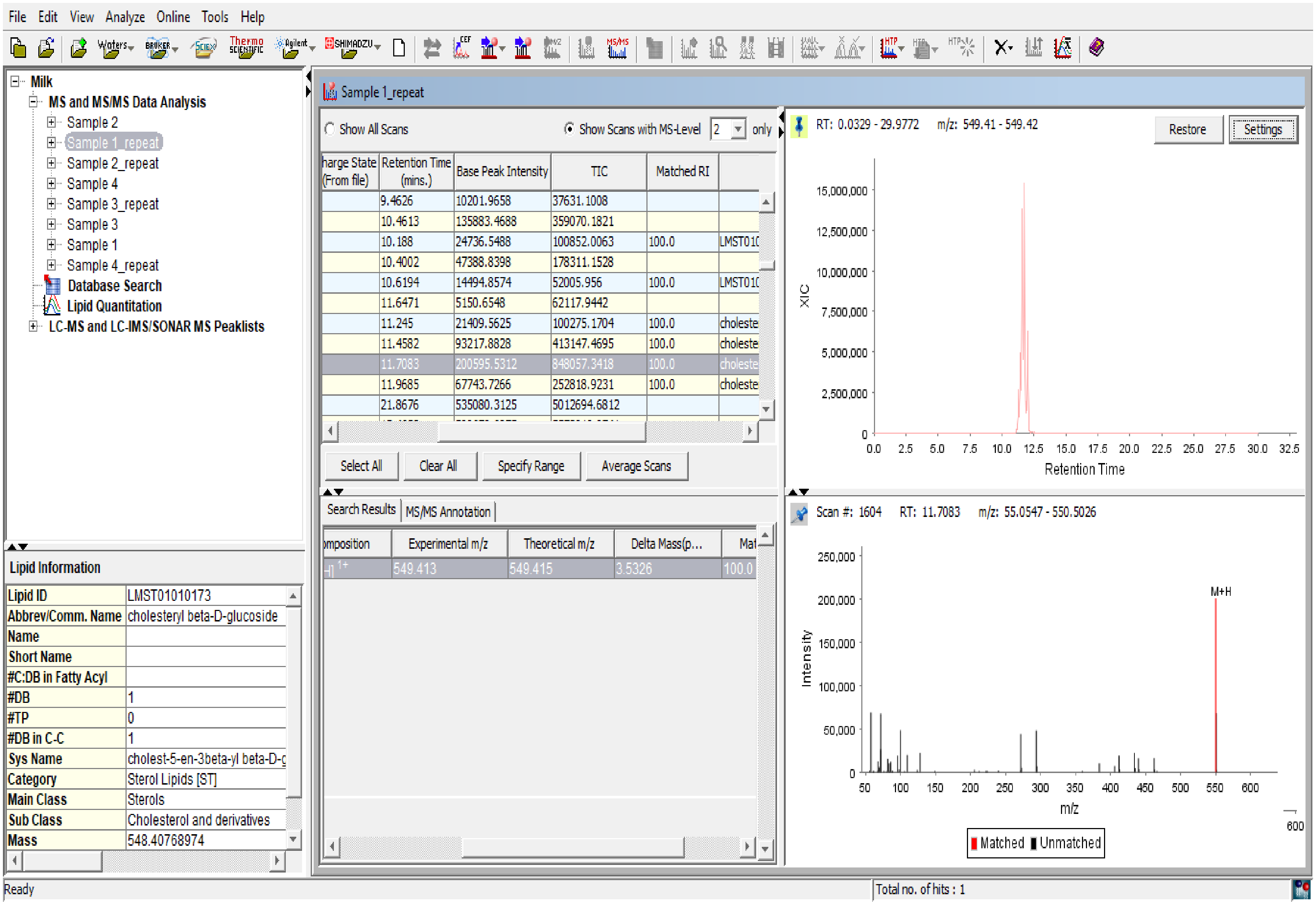

Figure S3: Typical SimLipid software GUI showing annotation of an MS/MS spectrum with a lipid ion that has been assigned Group-ID using MS excel in-built functions.

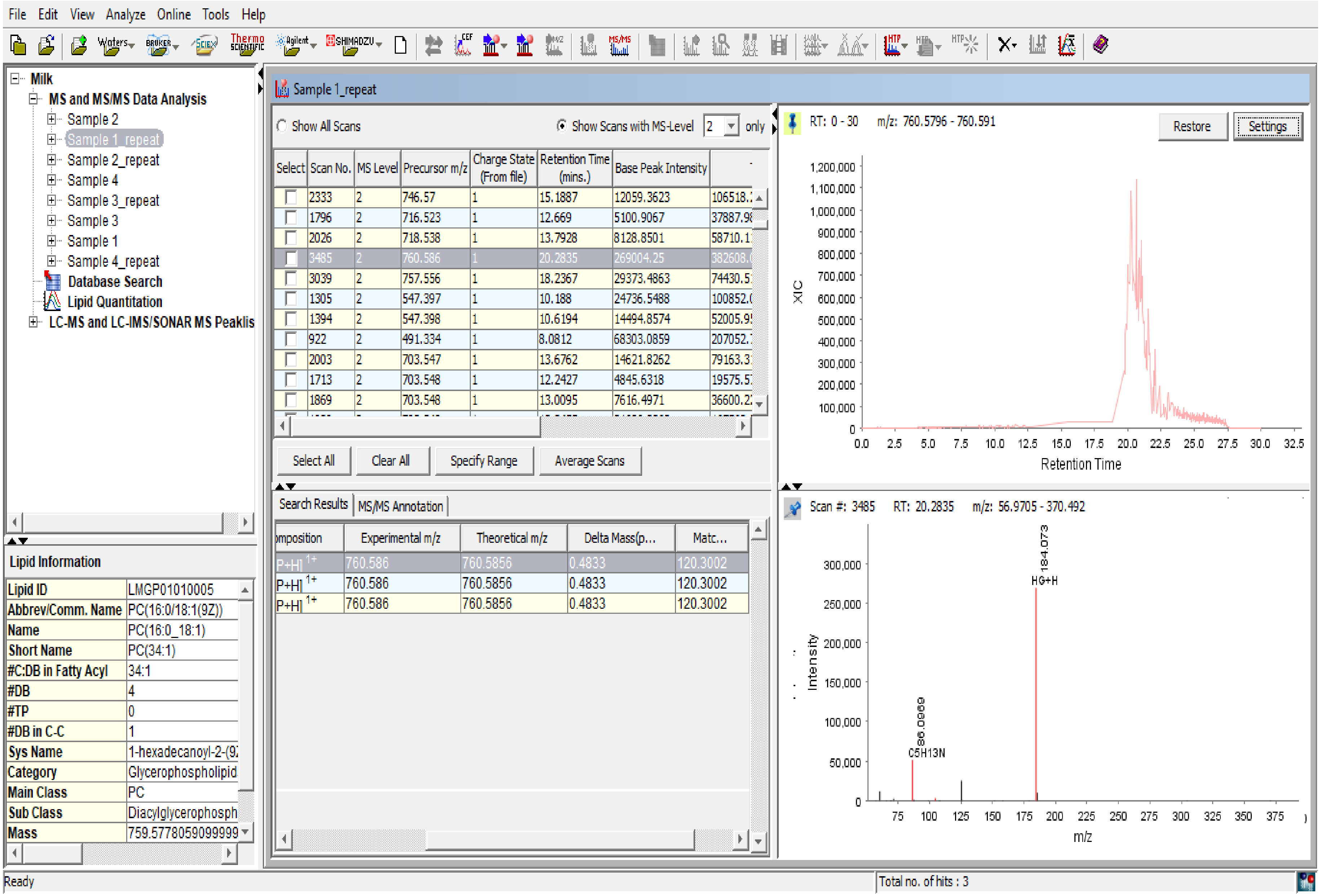

Select	Scan No.	MS Level	Precursor m/z	Charge State (From file)	Retention Time (mins.)	Base Peak Intensity	
	2333	2	746.57	1	15.1887	12059.3623	106518.[illegible]
	1796	2	716.523	1	12.669	5100.9067	37887.9[illegible]
	2026	2	718.538	1	13.7928	8128.8501	58710.1[illegible]
	3485	2	760.586	1	20.2835	269004.25	382608.[illegible]
	3039	2	757.556	1	18.2367	29373.4863	74430.5[illegible]
	1305	2	547.397	1	10.188	24736.5488	100852.[illegible]
	1394	2	547.398	1	10.6194	14494.8574	52005.9[illegible]
	922	2	491.334	1	8.0812	68303.0859	207052.[illegible]
	2003	2	703.547	1	13.6762	14621.8262	79163.3[illegible]
	1713	2	703.548	1	12.2427	4845.6318	19575.5[illegible]
	1869	2	703.548	1	13.0095	7616.4971	36600.2[illegible]

[illegible]mposition	Experimental m/z	Theoretical m/z	Delta Mass(p...	Matc...
P+H] 1+	760.586	760.5856	0.4833	120.3002
P+H] 1+	760.586	760.5856	0.4833	120.3002
P+H] 1+	760.586	760.5856	0.4833	120.3002

Figure S4: Typical SimLipid software GUI showing annotation of an MS/MS spectrum with a lipid ion that has been assigned PA-ID using MS excel in-built functions.

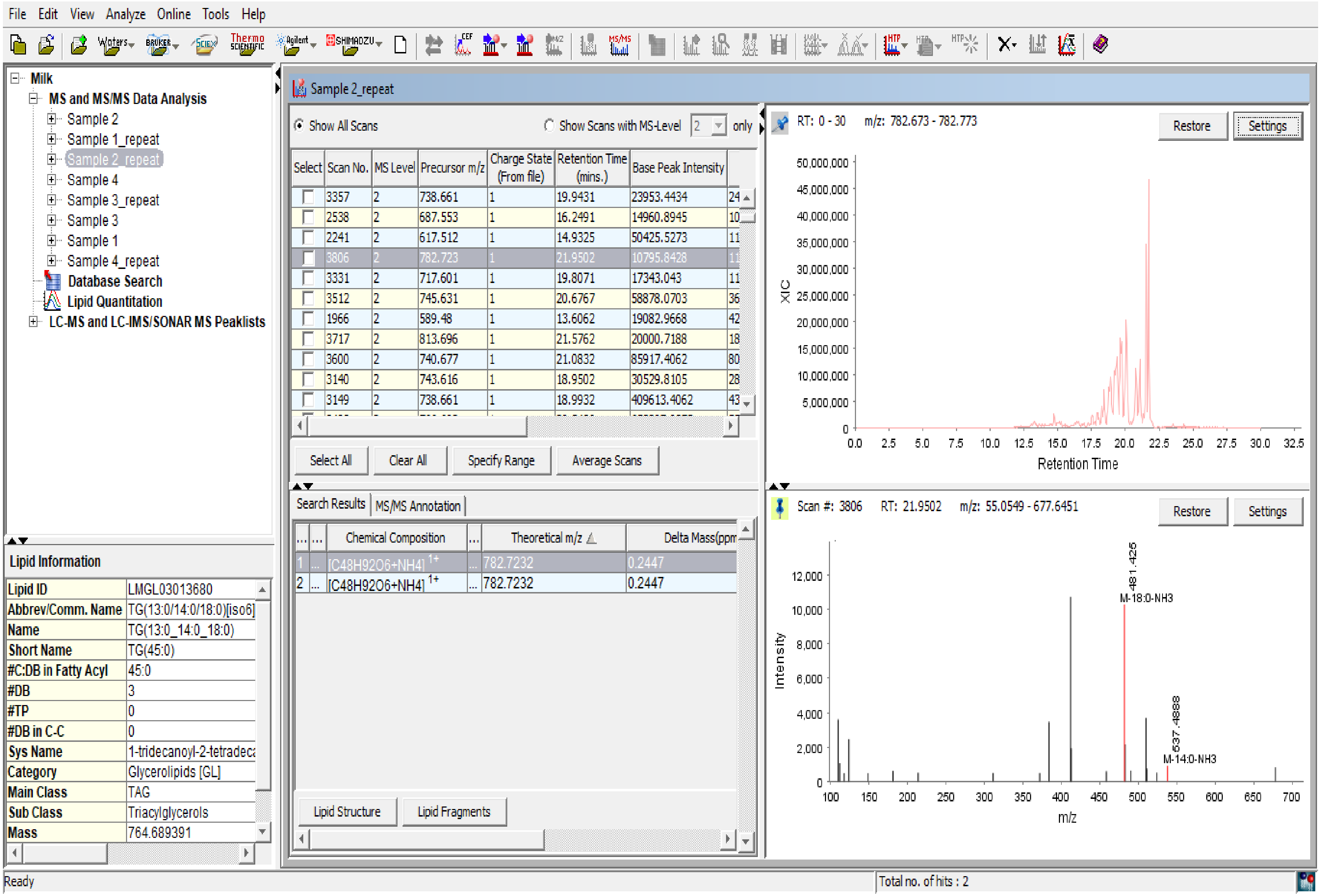

Figure S5: Typical SimLipid software GUI showing annotation of an MS/MS spectrum with a lipid ion that has been assigned FA-ID using MS excel in-built functions.

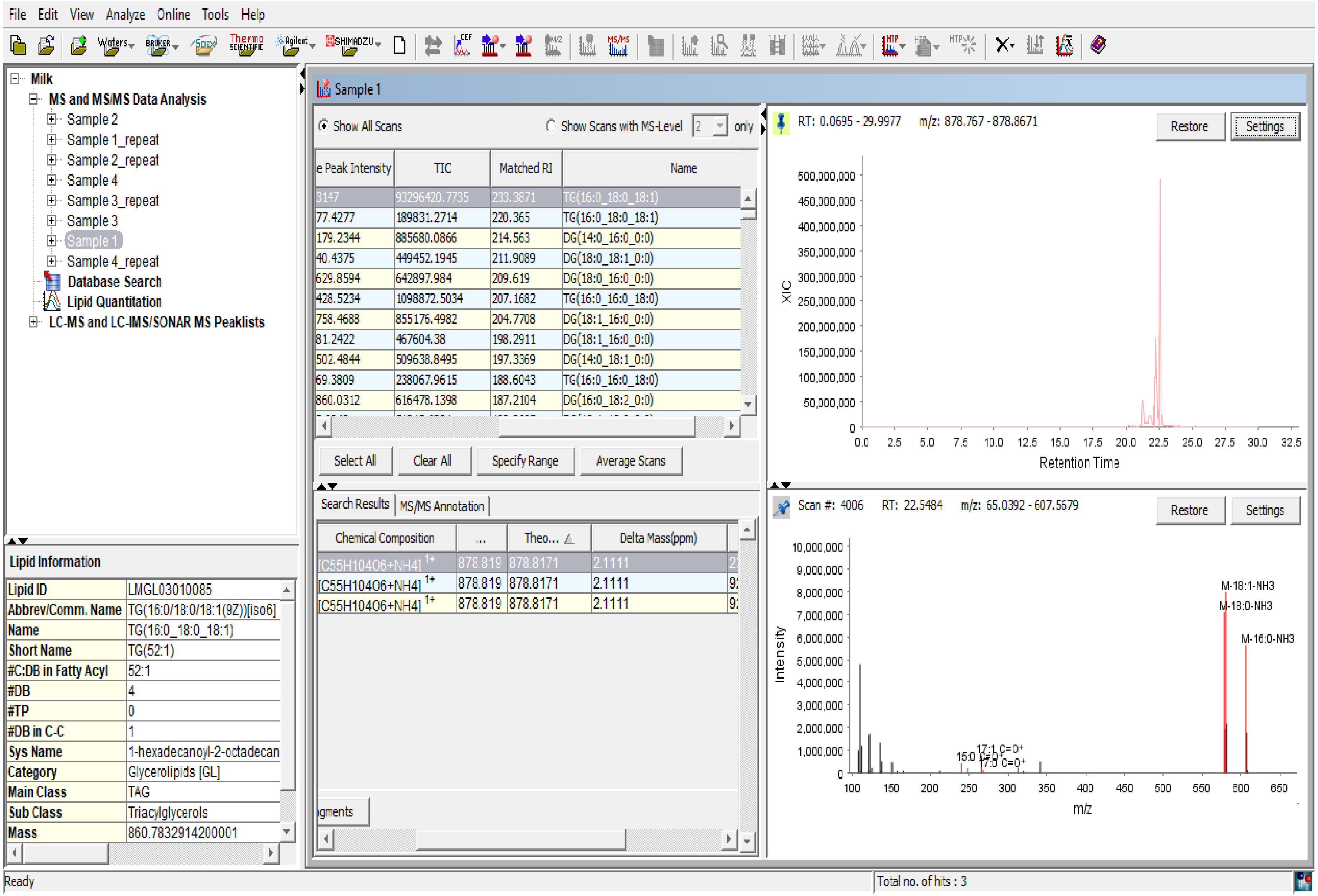

Figure S6: Manual annotation of possible fatty acid chains of the [TG 23:0 + NH4]+ ion using MS/MS data.

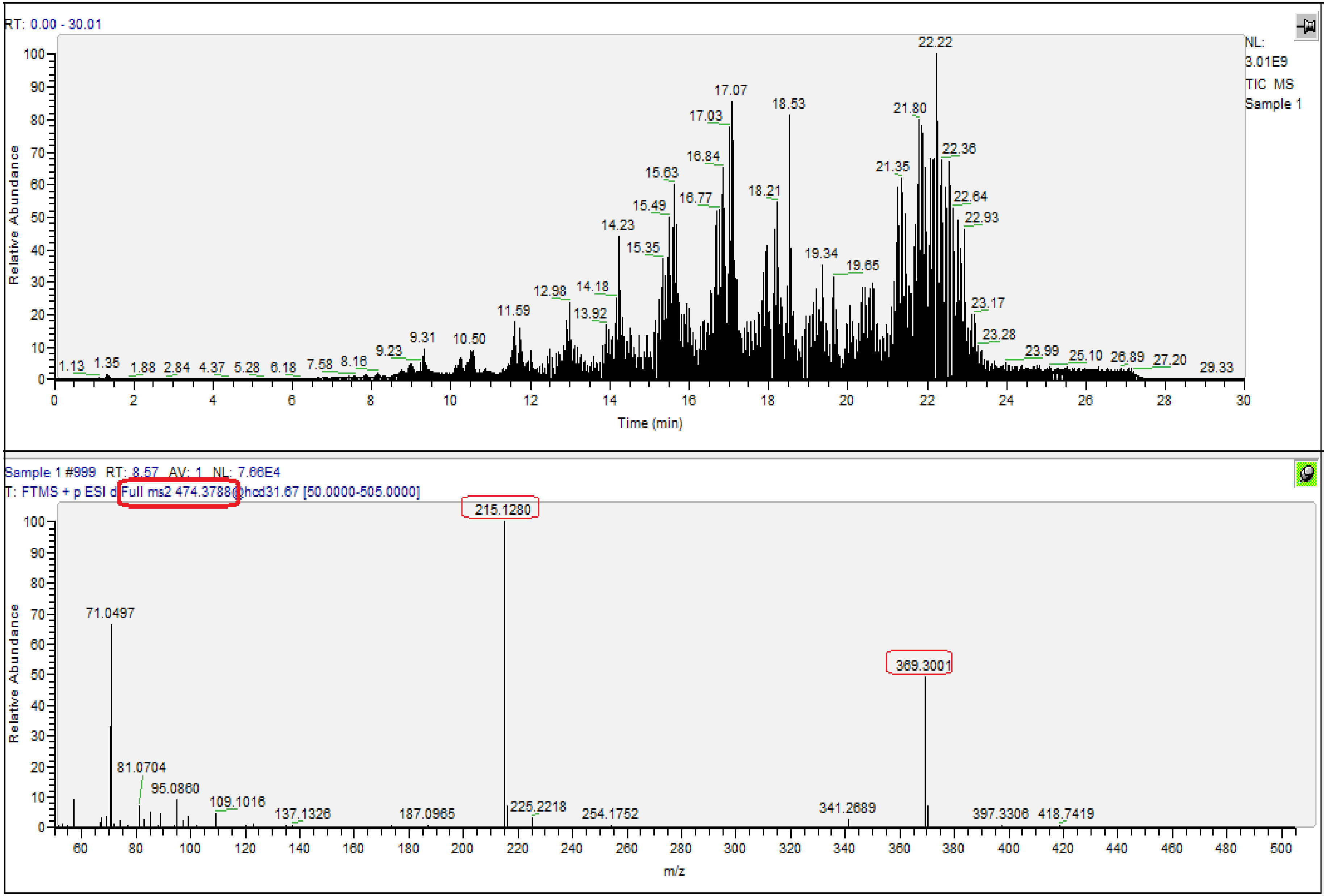

Figure S7: XIC of [PS 36:1+H]+ ion and isotopic cluster with a monoisotopic peak at m/z 791.5597.

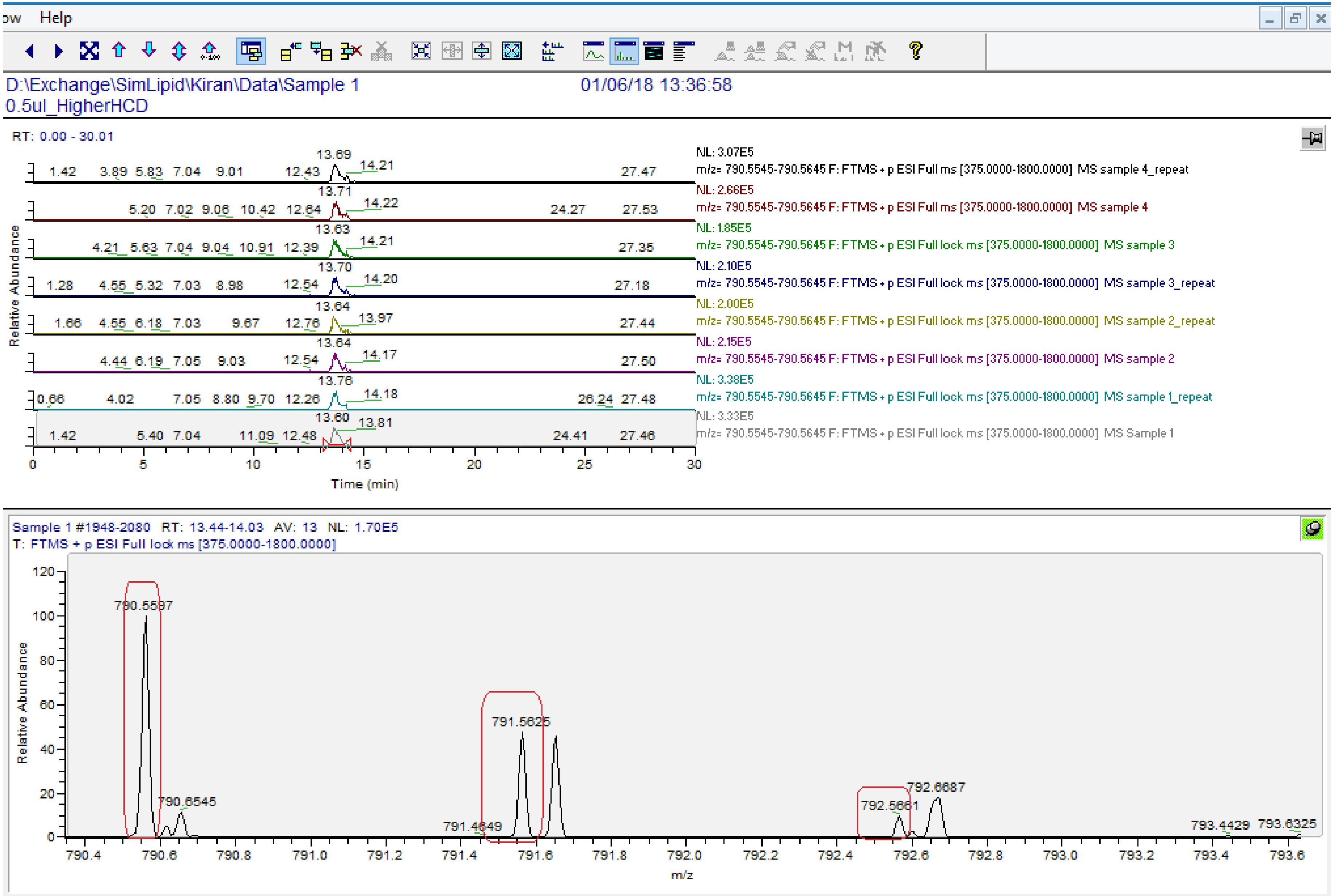

Figure S8 (1): An example MS/MS spectrum of TG 2:0_4:0_18:0 that contains 2:0 as one of the fatty acids.

File Name	Sample 1_repeat
Scan #	1199
Retention Time	9.617
m/z	488.3946
Chemical Composition	[C27H50O6+NH4] 1+
LIPIDMAPS Abbreviation/Common Name	TG(2:0/4:0/18:0)
Main Class	TAG
Sub Class	Triacylglycerols
Lipid ID	TG(2:0/4:0/18:0)
Theoretical m/z	488.3946
Delta Mass	0.0016 ppm
Group	TG
LSI ID	TG 2:0_4:0_18:0
Short Name	TG 24:0
Graded ID	TG 2:0_4:0_18:0
Matched Ions	3:0 C=O+_71.0497(67173.1719), M-18:0_187.0967(131318.3906), 17:0 C=O+_267.2687(2674.231), M-4:0_383.3159(35071.7305), M-2:0_411.3473(13118.2256)

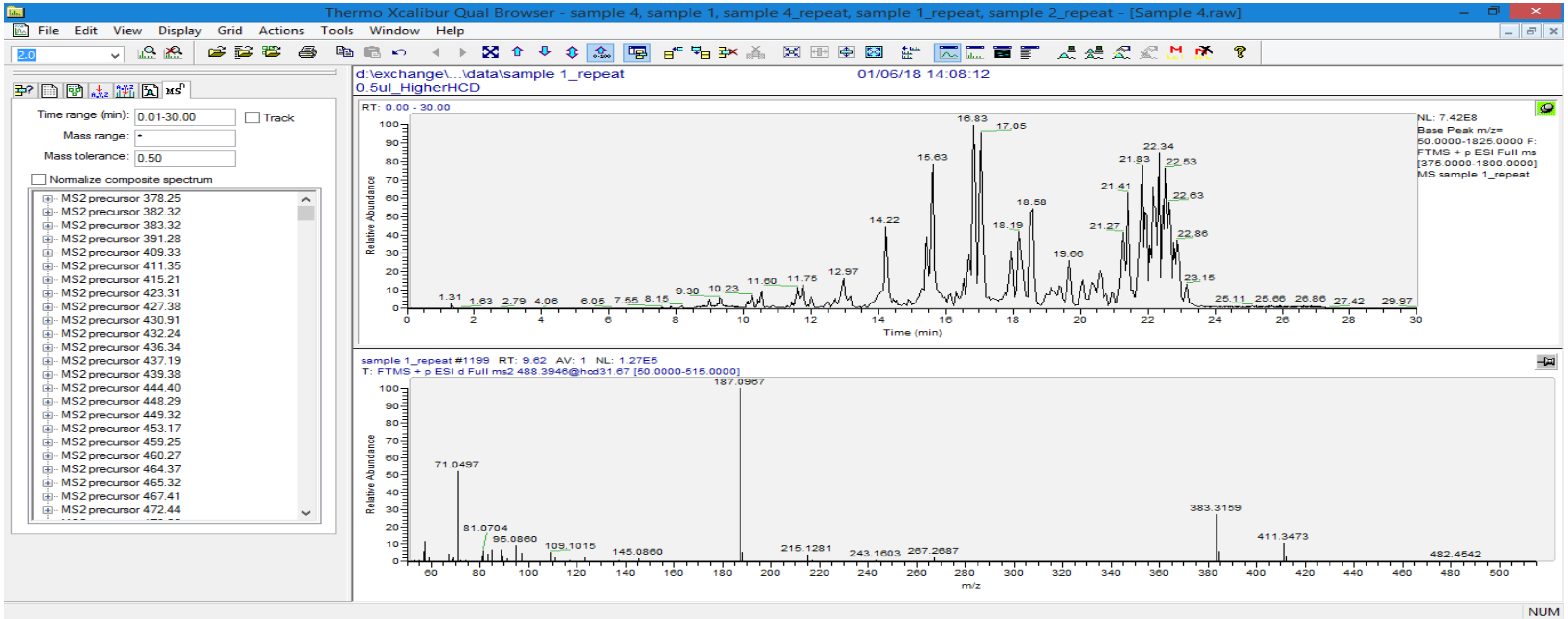

Figure S8 (2): An example MS/MS spectrum of TG 3:0_16:0_18:1 that contains 3:0 as one of the fatty acids.

File Name	Sample 4_repeat
Scan #	2602
Retention Time	16.3139
m/z	668.5829
Chemical Composition	[C40H74O6+NH4] 1+
LIPIDMAPS Abbreviation/Common Name	TG(3:0/16:0/18:1(11Z))
Main Class	TAG
Sub Class	Triacylglycerols
Lipid ID	TG(3:0/16:0/18:1(11Z))
Theoretical m/z	668.5824
Delta Mass	0.7813 ppm
Group	TG
LSI ID	TG 3:0_16:0_18:1
Short Name	TG 37:1
Graded ID	TG 3:0_16:0_18:1
Matched Ions	2:0 C=O+_57.0342(13167.042), 15:0 C=O+_239.2364(2009.1318), 17:1 C=O+_265.2527(13355.249), M-18:1_369.3007(230710.6875), M-16:0_395.3157(52155.8242), M-3:0_577.5251(3330.9688)

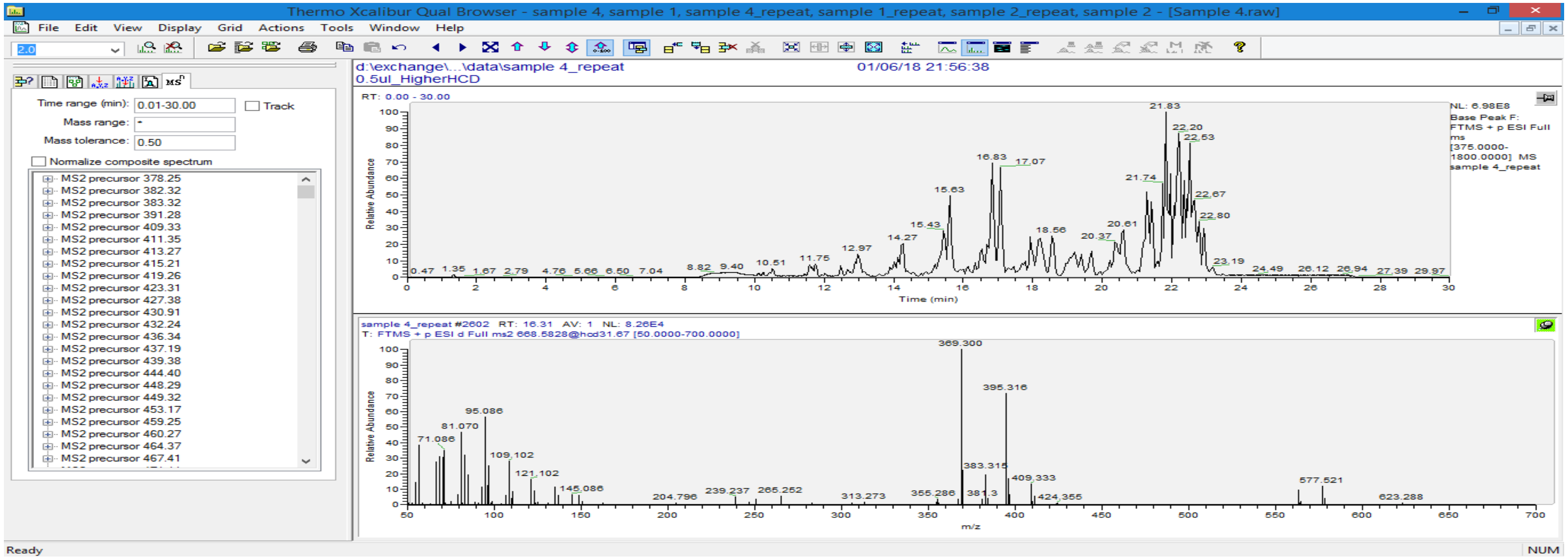

Figure S8 (3): An example MS/MS spectrum of TG 4:0_6:0_14:0 that contains the fatty acids 4:0 and 14:0.

File Name	Sample 1
Scan #	1071
Retention Time	8.9564
m/z	488.3948
Chemical Composition	[C27H50O6+NH4] 1+
LIPIDMAPS Abbreviation/Common Name	TG(4:0/6:0/14:0)
Main Class	TAG
Sub Class	Triacylglycerols
Lipid ID	TG(4:0/6:0/14:0)
Theoretical m/z	488.3946
Delta Mass	0.4983 ppm
Group	TG
LSI ID	TG 4:0_6:0_14:0
Short Name	TG 24:0
Graded ID	TG 4:0_6:0_14:0
Matched Ions	3:0 C=O+_71.0498(419481.9688), 5:0 C=O+_99.081(494175.8438), 13:0 C=O+_211.206(51658.6953), M-14:0_243.1596(1127776.875), M-6:0_355.2848(335377.0938), M-4:0_383.3162(361150.9375)

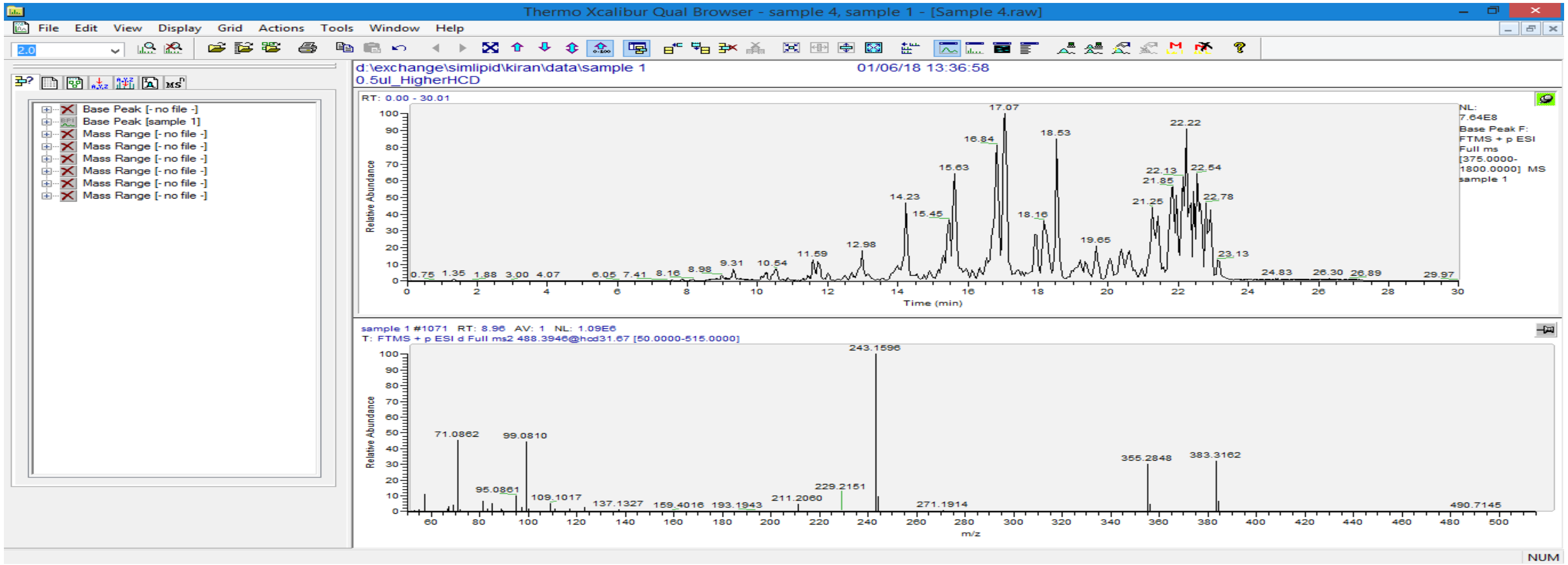

Figure S8 (4): An example MS/MS spectrum of TG 4:0_5:0_16:0 that contains 5:0 as one of the fatty acids.

File Name	Sample 1
Scan #	1254
Retention Time	9.9184
m/z	502.4103
Chemical Composition	[C28H52O6+NH4] 1+
LIPIDMAPS Abbreviation/Common Name	TG(4:0/5:0/16:0)
Main Class	TAG
Sub Class	Triacylglycerols
Lipid ID	TG(4:0/5:0/16:0)
Theoretical m/z	502.4102
Delta Mass	0.2523 ppm
Group	TG
LSI ID	TG 4:0_5:0_16:0
Short Name	TG 25:0
Graded ID	TG 4:0_5:0_16:0
Matched Ions	3:0 C=O+_71.0497(12248.4922), 4:0 C=O+_85.0653(18084.1992), M-16:0_229.1437(49173.2695), 15:0 C=O+_239.2364(496.1783), M-5:0_383.3163(9394.6094), M-4:0_397.3313(11000.4111)

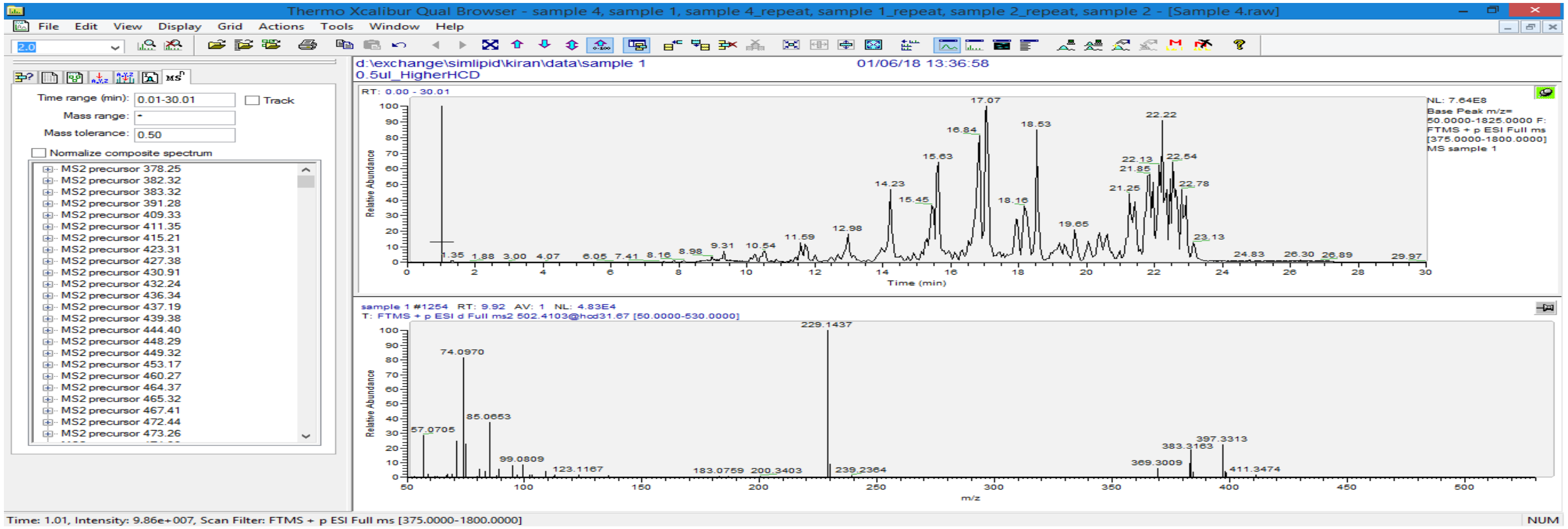

Figure S8 (5): An example MS/MS spectrum of TG(4:0/6:0/14:0) that contains 6:0 as one of the fatty acids.

File Name	Sample 1
Scan #	1071
Retention Time	8.9564
m/z	488.3948
Chemical Composition	[C27H50O6+NH4] 1+
LIPIDMAPS Abbreviation/Common Name	TG(4:0/6:0/14:0)
Main Class	TAG
Sub Class	Triacylglycerols
Lipid ID	TG(4:0/6:0/14:0)
Theoretical m/z	488.3946
Delta Mass	0.4983 ppm
Group	TG
LSI ID	TG 4:0_6:0_14:0
Short Name	TG 24:0
Graded ID	TG 4:0_6:0_14:0
Matched Ions	3:0 C=O+_71.0498(419481.9688), 5:0 C=O+_99.081(494175.8438), 13:0 C=O+_211.206(51658.6953), M-14:0_243.1596(1127776.875), M-6:0_355.2848(335377.0938), M-4:0_383.3162(361150.9375)

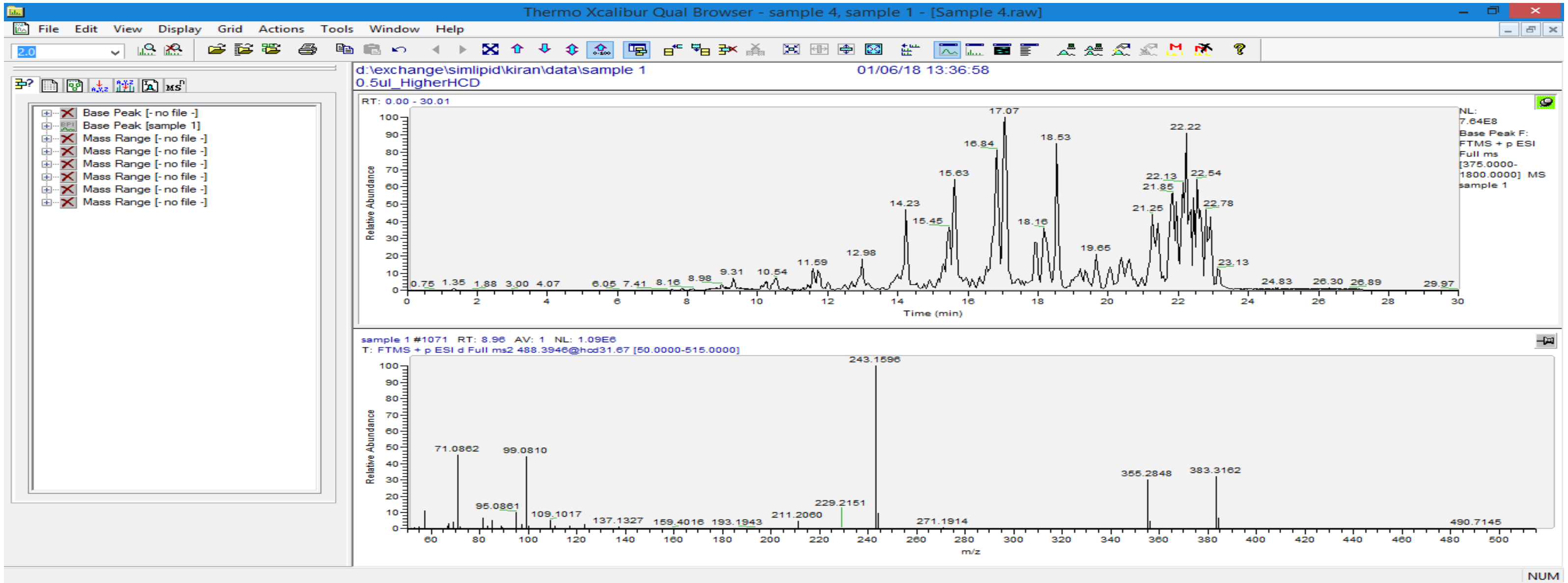

Figure S8 (6): An example MS/MS spectrum of TG 5:0_7:0_15:0 that contains the fatty acids 7:0 and 15:0.

File Name	Sample 1_repeat
Scan #	1480
Retention Time	11.0791
m/z	530.4417
Chemical Composition	[C30H56O6+NH4] 1+
LIPIDMAPS Abbreviation/Common Name	TG(5:0/7:0/15:0)
Main Class	TAG
Sub Class	Triacylglycerols
Lipid ID	TG(5:0/7:0/15:0)
Theoretical m/z	530.4415
Delta Mass	0.3746 ppm
Group	TG
LSI ID	TG 5:0_7:0_15:0
Short Name	TG 27:0
Graded ID	TG 5:0_7:0_15:0
Matched Ions	4:0 C=O+_85.0653(8174.5039), 6:0 C=O+_113.0961(3476.3364), 14:0 C=O+_225.2221(985.0714), M-15:0_271.1904(39955.7969), M-7:0_383.3148(2001.8545), M-5:0_411.347(8337.6562)

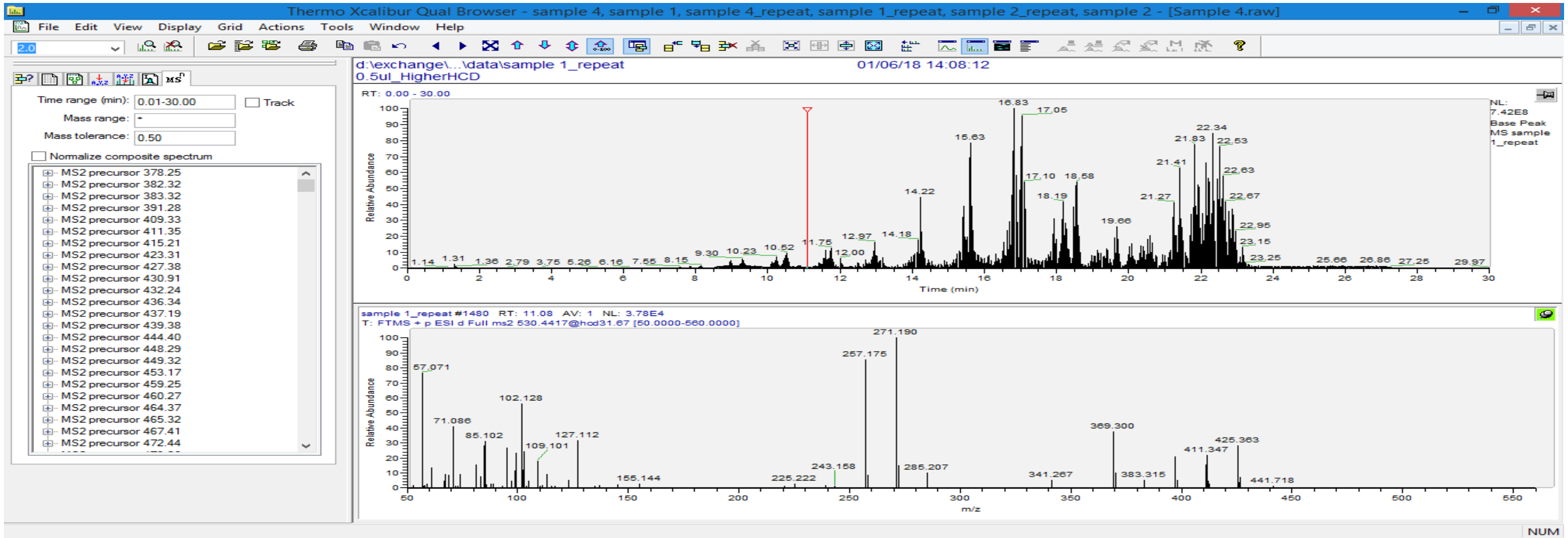

Figure S8 (7): An example MS/MS spectrum of TG(4:0_8:0_12:0) that contains the fatty acids 8:0 and 12:0.

File Name	Sample 1
Scan #	1032
Retention Time	8.7537
m/z	488.3946
Chemical Composition	[C27H50O6+NH4] 1+
LIPIDMAPS Abbreviation/Common Name	TG(4:0/8:0/12:0)
Main Class	TAG
Sub Class	Triacylglycerols
Lipid ID	TG(4:0/8:0/12:0)
Theoretical m/z	488.3946
Delta Mass	0.1234 ppm
Group	TG
LSI ID	TG 4:0_8:0_12:0
Short Name	TG 24:0
Graded ID	TG 4:0_8:0_12:0
Matched Ions	3:0 C=O+_71.0498(128767.0156), 7:0 C=O+_127.1121(118972.9922), 11:0 C=O+_183.1746(27360.0098), M-12:0_271.1907(333331.375), M-8:0_327.2534(131130.4375), M-4:0_383.316(119953.125)

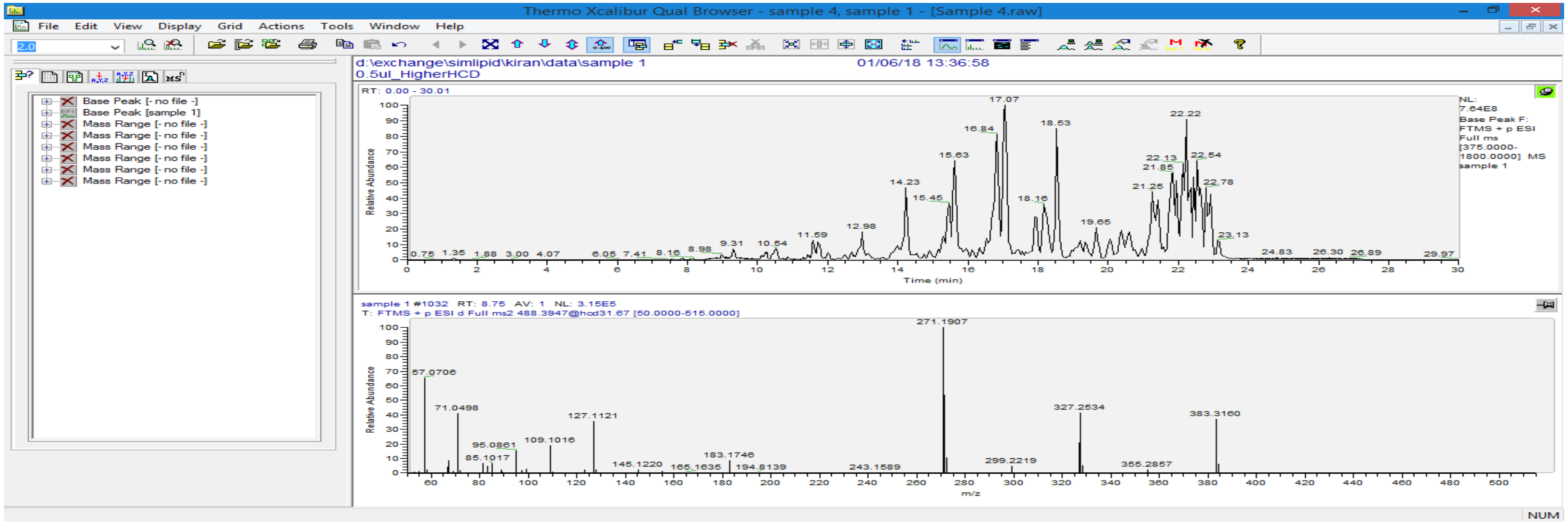

Figure S8 (8): An example MS/MS spectrum of TG 9:0_14:0_18:0 that contains 9:0 as one of the fatty acids.

File Name	Sample 1
Scan #	3436
Retention Time	20.0729
m/z	726.661
Chemical Composition	[C44H84O6+NH4] 1+
LIPIDMAPS Abbreviation/Common Name	TG(9:0/14:0/18:0)
Main Class	TAG
Sub Class	Triacylglycerols
Lipid ID	TG(9:0/14:0/18:0)
Theoretical m/z	726.6606
Delta Mass	0.5464 ppm
Group	TG
LSI ID	TG 9:0_14:0_18:0
Short Name	TG 41:0
Graded ID	TG 9:0_14:0_18:0
Matched Ions	8:0 C=O+_141.1293(930.9376), M-18:0_425.3634(30112.082), M-14:0_481.4262(3743.8767), M-9:0_551.5023(2015.0214)

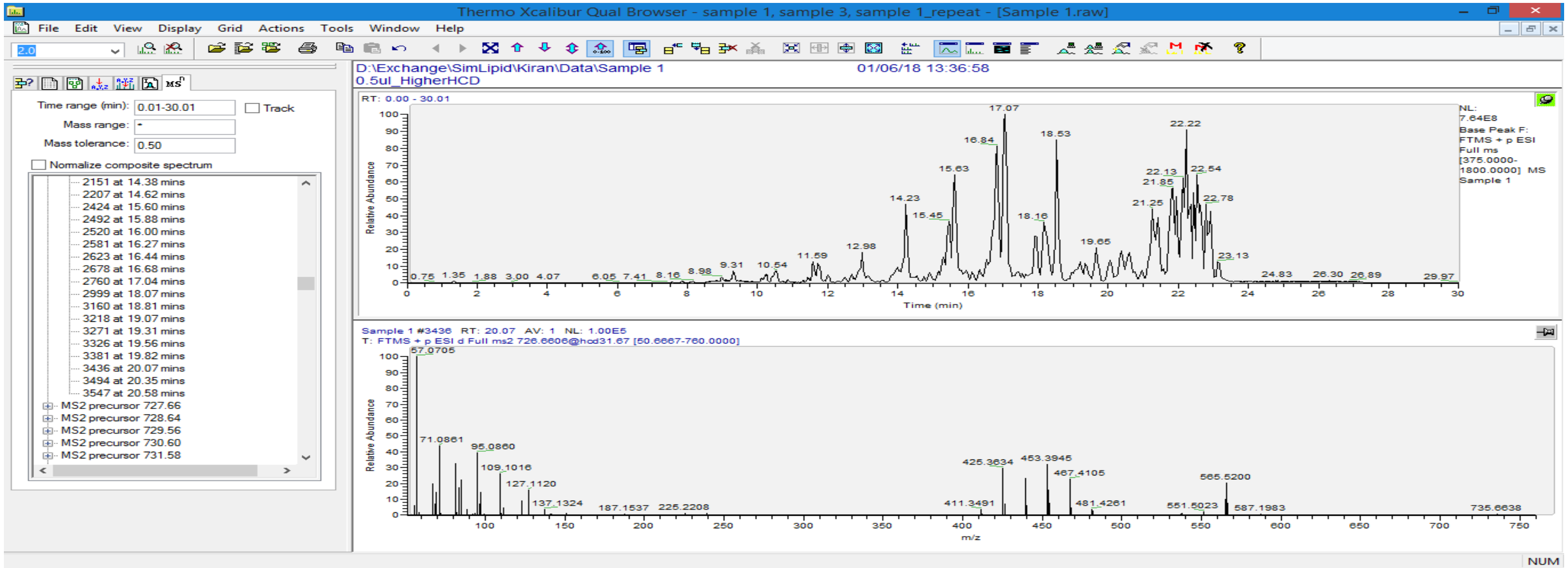

Figure S8 (9): An example MS/MS spectrum of TG 10:0_12:0_14:0 that contains 10:0 as one of the fatty acids.

File Name	Sample 1
Scan #	2609
Retention Time	16.3923
m/z	656.5826
Chemical Composition	[C39H74O6+NH4] 1+
LIPIDMAPS Abbreviation/Common Name	TG(10:0/12:0/14:0)
Main Class	TAG
Sub Class	Triacylglycerols
Lipid ID	TG(10:0/12:0/14:0)
Theoretical m/z	656.5824
Delta Mass	0.4238 ppm
Group	TG
LSI ID	TG 10:0_12:0_14:0
Short Name	TG 36:0
Graded ID	TG 10:0_12:0_14:0
Matched Ions	9:0 C=O+_155.1431(168913.8906), 11:0 C=O+_183.1742(21730.877), 13:0 C=O+_211.206(25832.4473), M-14:0_411.3474(312534.125), M-12:0_439.3783(111215.4609), M-10:0_467.4102(427428.625)

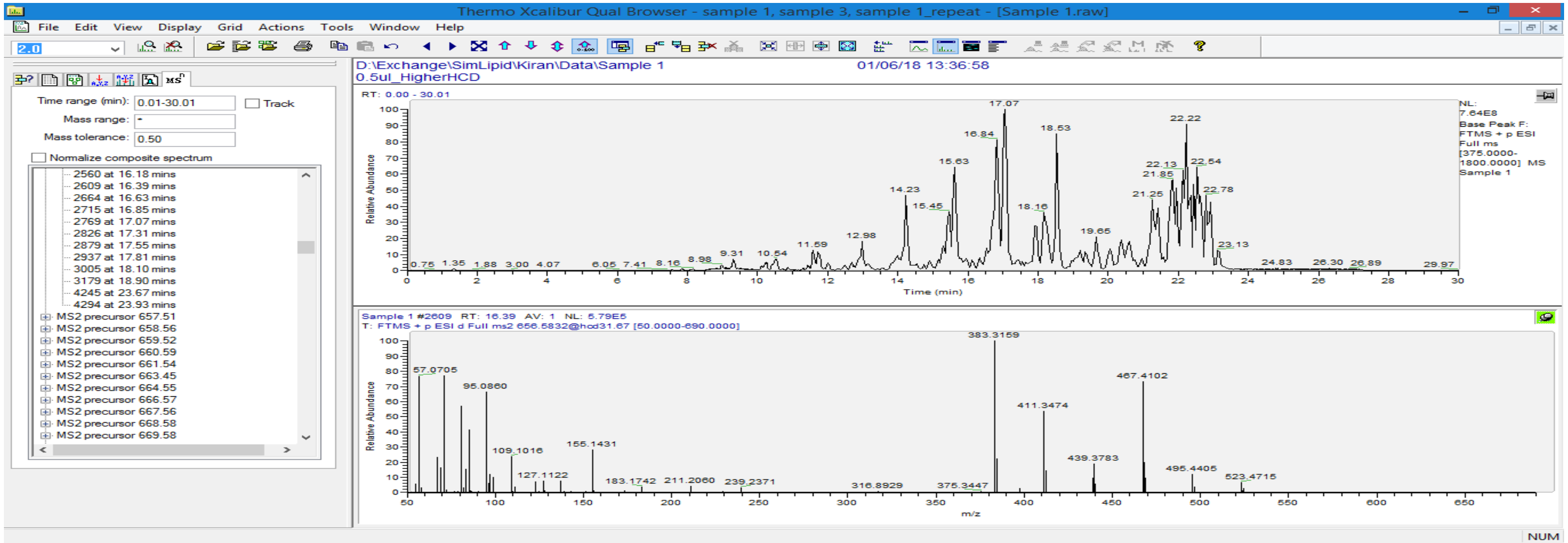

Figure S8 (10): An example MS/MS spectrum of TG 4:0_10:1_14:0 that contains 10:1 as one of the fatty acids.

File Name	Sample 4
Scan #	1445
Retention Time	10.5883
m/z	542.442
Chemical Composition	[C31H56O6+NH4] 1+
LIPIDMAPS Abbreviation/Common Name	TG(4:0/10:1(6Z)/14:0)
Main Class	TAG
Sub Class	Triacylglycerols
Lipid ID	TG(4:0/10:1(6Z)/14:0)
Theoretical m/z	542.4415
Delta Mass	0.9289 ppm
Group	TG
LSI ID	TG 4:0_10:1_14:0
Short Name	TG 28:1
Graded ID	TG 4:0_10:1_14:0
Matched Ions	3:0 C=O+_71.0497(67722.3516), 9:1 C=O+_153.1273(2776.2185), 13:0 C=O+_211.2059(8476.6777), M-14:0_297.2062(150994.6094), M-10:1_355.2845(63909.4688), M-4:0_437.3628(52527.8789)

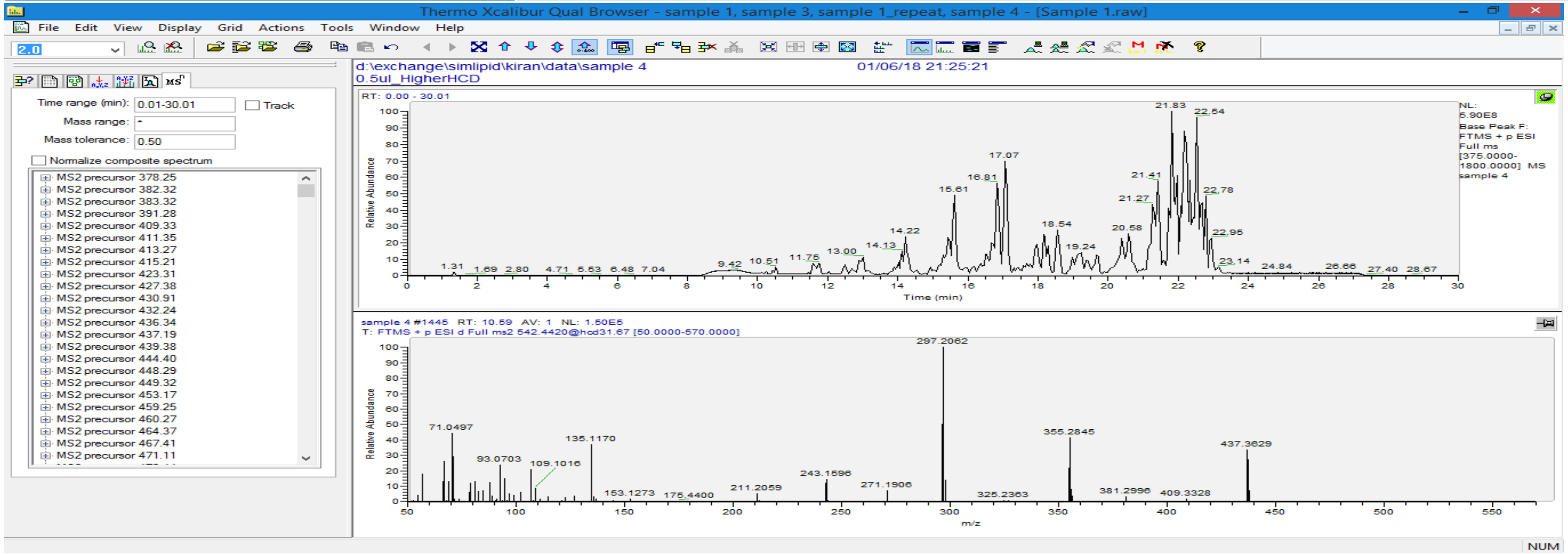

Figure S8 (11): An example MS/MS spectrum of TG 11:0_14:0_16:0 that contains 11:0 as one of the fatty acids.

File Name	Sample 4_repeat
Scan #	3402
Retention Time	19.9151
m/z	726.6612
Chemical Composition	[C44H84O6+NH4] 1+
LIPIDMAPS Abbreviation/Common Name	TG(11:0/14:0/16:0)
Main Class	TAG
Sub Class	Triacylglycerols
Lipid ID	TG(11:0/14:0/16:0)
Theoretical m/z	726.6606
Delta Mass	0.7983 ppm
Group	TG
LSI ID	TG 11:0_14:0_16:0
Short Name	TG 41:0
Graded ID	TG 11:0_14:0_16:0
Matched Ions	13:0 C=O+_211.206(3603.4819), 15:0 C=O+_239.2368(3294.7551), M-16:0_453.3942(86159.9375), M-14:0_481.4254(33482.5508), M-11:0_523.4719(10786.8057)

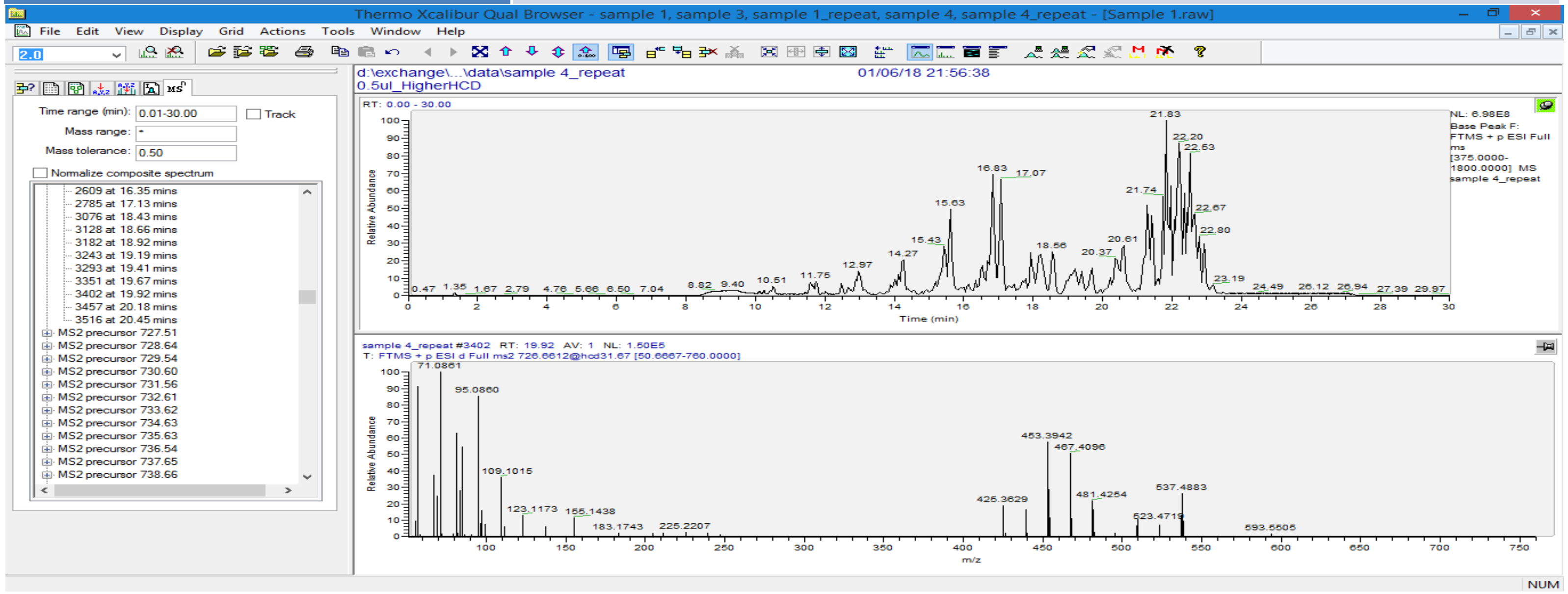

Figure S8 (12): An example MS/MS spectrum of TG 6:0_12:1_14:1 that contains the fatty acids 12:1 and 14:1.

File Name	Sample 4_repeat
Scan #	1626
Retention Time	11.6683
m/z	596.4892
Chemical Composition	[C35H62O6+NH4] 1+
LIPIDMAPS Abbreviation/Common Name	TG(6:0/12:1(6Z)/14:1(9Z))
Main Class	TAG
Sub Class	Triacylglycerols
Lipid ID	TG(6:0/12:1(6Z)/14:1(9Z))
Theoretical m/z	596.4885
Delta Mass	1.2302 ppm
Group	TG
LSI ID	TG 6:0_12:1_14:1
Short Name	TG 32:2
Graded ID	TG 6:0_12:1_14:1
Matched Ions	5:0 C=O+_99.081(17267.3457), 13:1 C=O+_209.1903(6066.4502), M-14:1_353.2692(57611.8242), M-12:1_381.3004(2242.2351), M-6:0_463.3791(12493.7334)

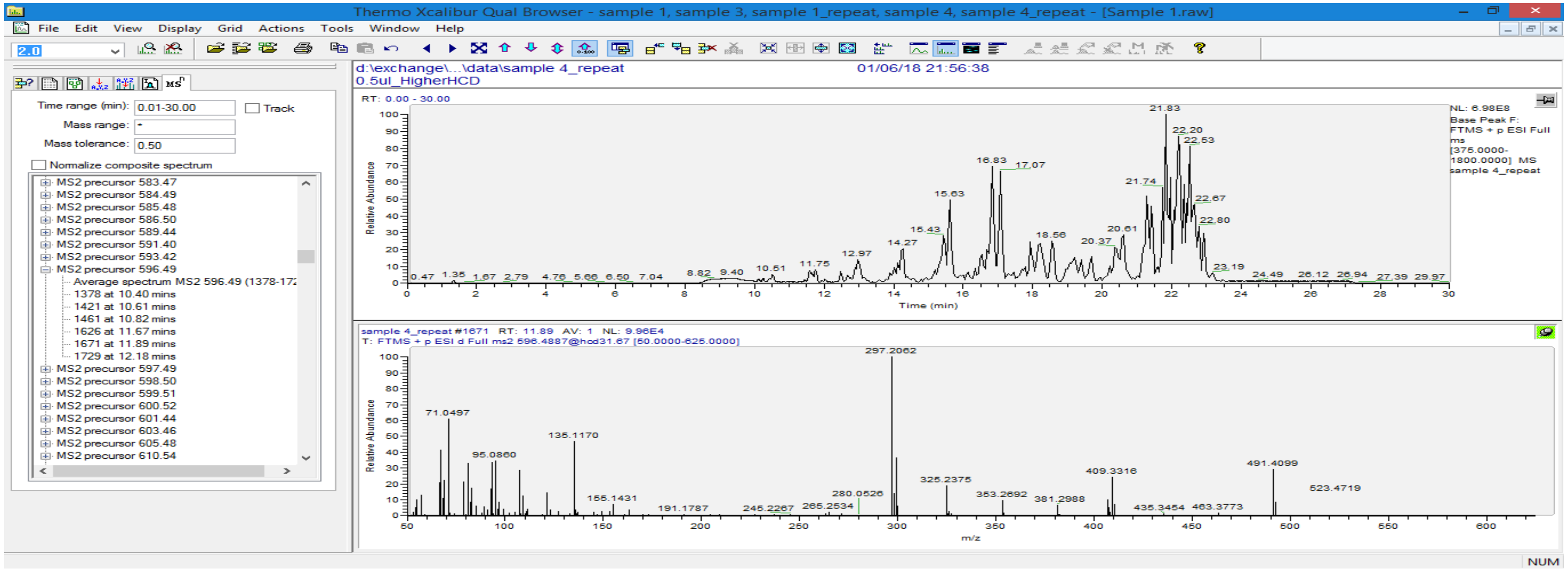

Figure S8 (13): An example MS/MS spectrum of TG 8:0_10:0_13:0 that contains 13:0 as one of the fatty acids.

File Name	Sample 1
Scan #	1937
Retention Time	13.3493
m/z	586.5044
Chemical Composition	[C34H64O6+NH4] 1+
LIPIDMAPS Abbreviation/Common Name	TG(8:0/10:0/13:0)
Main Class	TAG
Sub Class	Triacylglycerols
Lipid ID	TG(8:0/10:0/13:0)
Theoretical m/z	586.5041
Delta Mass	0.48 ppm
Group	TG
LSI ID	TG 8:0_10:0_13:0
Short Name	TG 31:0
Graded ID	TG 8:0_10:0_13:0
Matched Ions	7:0 C=O+_127.1121(3522.7087), 9:0 C=O+_155.1433(23203.0977), M-13:0_355.2853(8719.9727), M-10:0_397.3314(50397.6055), M-8:0_425.3625(4267.0239)

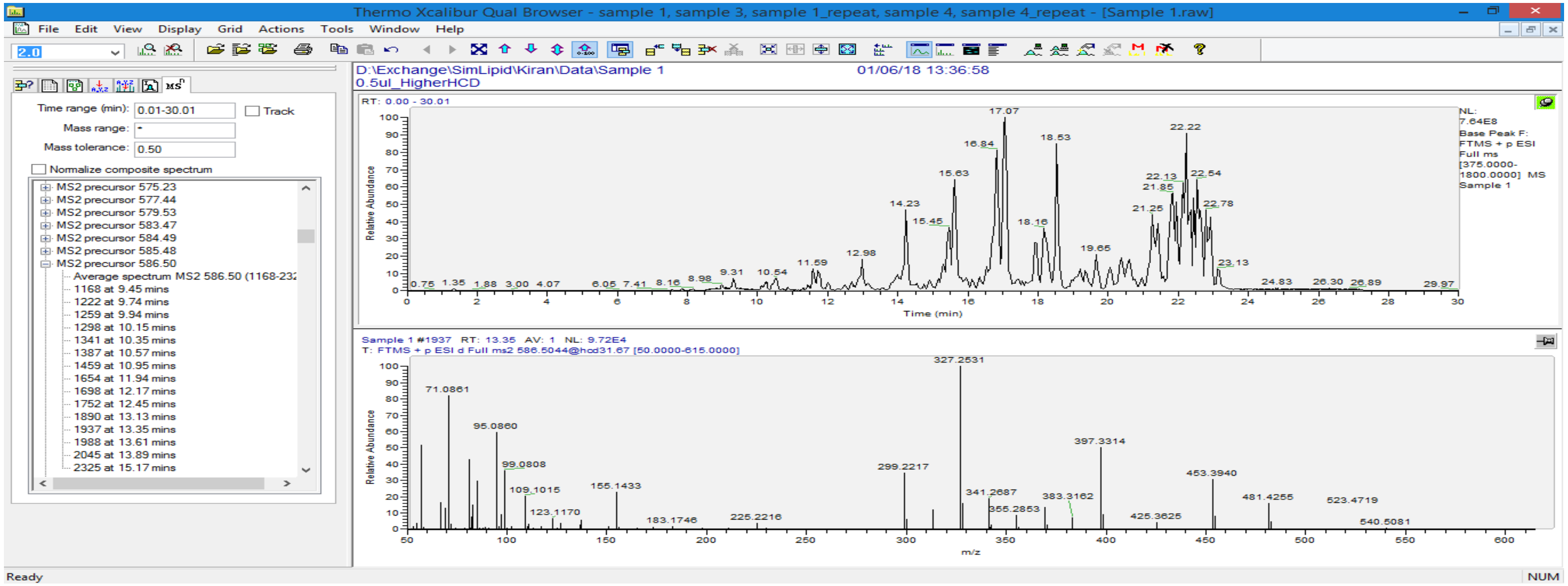

Figure S8 (14): An example MS/MS spectrum of TG 4:0_15:1_16:0 that contains 15:1 as one of the fatty acids.

File Name	Sample 1_repeat
Scan #	2386
Retention Time	15.4225
m/z	640.5518
Chemical Composition	[C38H70O6+NH4] 1+
LIPIDMAPS Abbreviation/Common Name	TG(4:0/15:1(9Z)/16:0)
Main Class	TAG
Sub Class	Triacylglycerols
Lipid ID	TG(4:0/15:1(9Z)/16:0)
Theoretical m/z	640.5511
Delta Mass	1.0844 ppm
Group	TG
LSI ID	TG 4:0_15:1_16:0
Short Name	TG 35:1
Graded ID	TG 4:0_15:1_16:0
Matched Ions	4:0 C=O+_85.0653(1316.6154), 14:1 C=O+_223.2063(2310.807617), 15:0 C=O+_239.2373(1806.424683), M-15:1_383.318345(37593.15625),M-4:0_535.470424(2333.004883),M-16:0_367.282197(9641.953125)

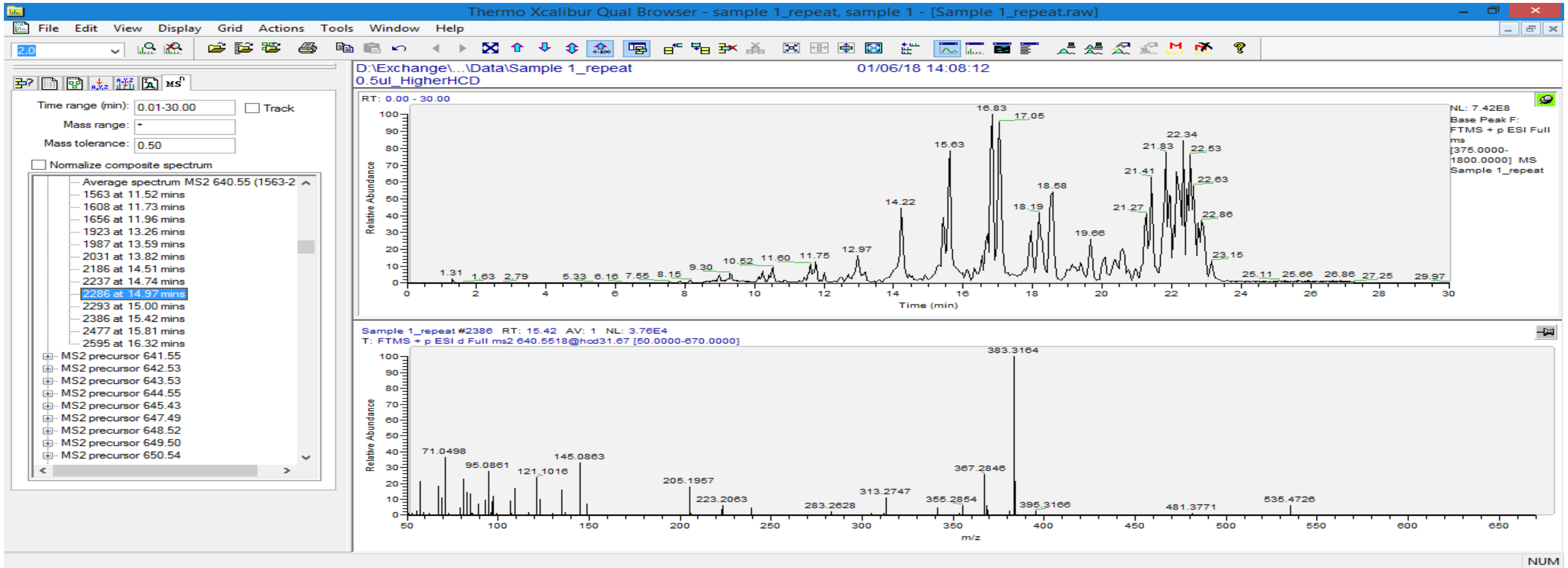

Figure S8 (15): An example MS/MS spectrum of TG 6:0_10:0_16:2 that contains 16:2 as one of the fatty acids.

File Name	Sample 1_repeat
Scan #	1634
Retention Time	11.8414
m/z	596.4887
Chemical Composition	[C35H62O6+NH4] 1+
LIPIDMAPS Abbreviation/Common Name	TG(6:0/10:0/16:2(9Z,12Z))
Main Class	TAG
Sub Class	Triacylglycerols
Lipid ID	TG(6:0/10:0/16:2(9Z,12Z))
Theoretical m/z	596.4885
Delta Mass	0.4117 ppm
Group	TG
LSI ID	TG 6:0_10:0_16:2
Short Name	TG 32:2
Graded ID	TG 6:0_10:0_16:2
Matched Ions	5:0 C=O+_99.0808(12754.6553), 9:0 C=O+_155.1433(29190.7754), M-16:2_327.2532(2940.1582), M-10:0_407.3159(37164.3672), M-6:0_463.3799(7078.8677)

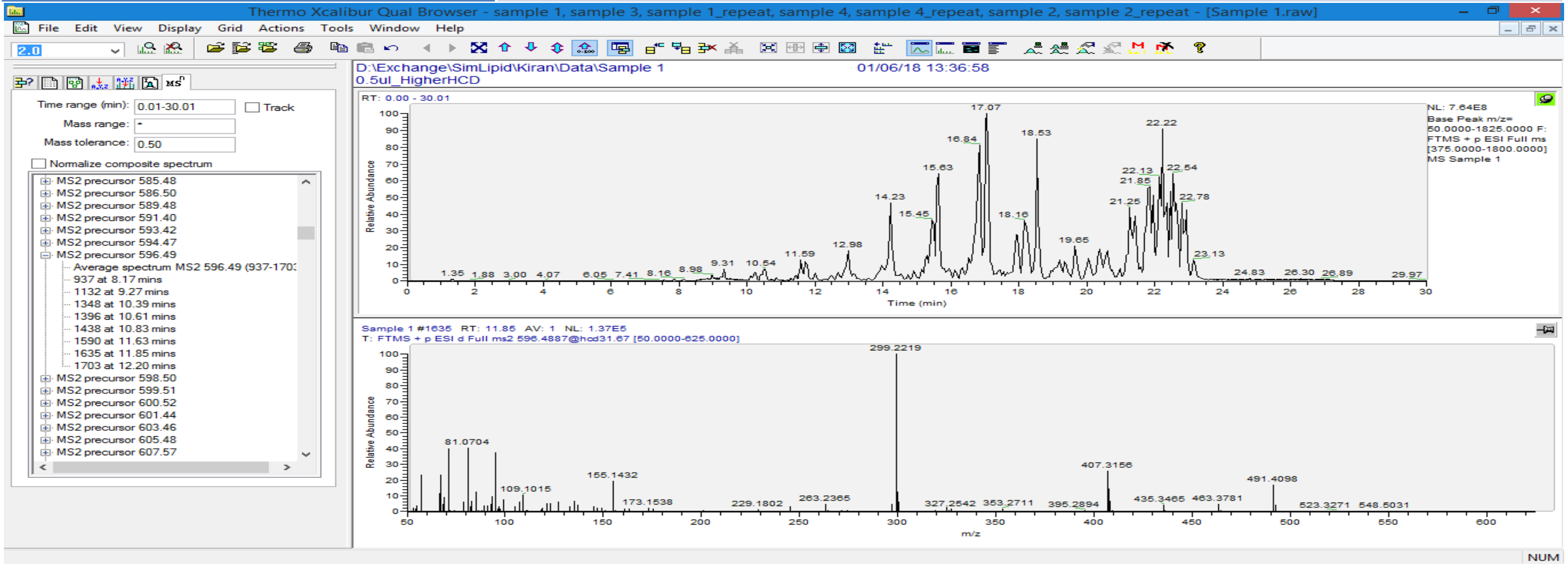

Figure S8 (16): An example MS/MS spectrum of TG 4:0_16:0_17:0 that contains 17:0 as one of the fatty acids.

File Name	Sample 1_repeat
Scan #	2942
Retention Time	17.812
m/z	670.5983
Chemical Composition	[C40H76O6+NH4] 1+
LIPIDMAPS Abbreviation/Common Name	TG(4:0/16:0/17:0)
Main Class	TAG
Sub Class	Triacylglycerols
Lipid ID	TG(4:0/16:0/17:0)
Theoretical m/z	670.598
Delta Mass	0.4685 ppm
Group	TG
LSI ID	TG 4:0_16:0_17:0
Short Name	TG 37:0
Graded ID	TG 4:0_16:0_17:0
Matched Ions	3:0 C=O+_71.0498(826046.3125), 15:0 C=O+_239.2373(69039.6484), 16:0 C=O+_253.2529(43307.3164), M-17:0_383.3158(893575.8125), M-16:0_397.3315(834546.625), M-4:0_565.5202(387019.2812)

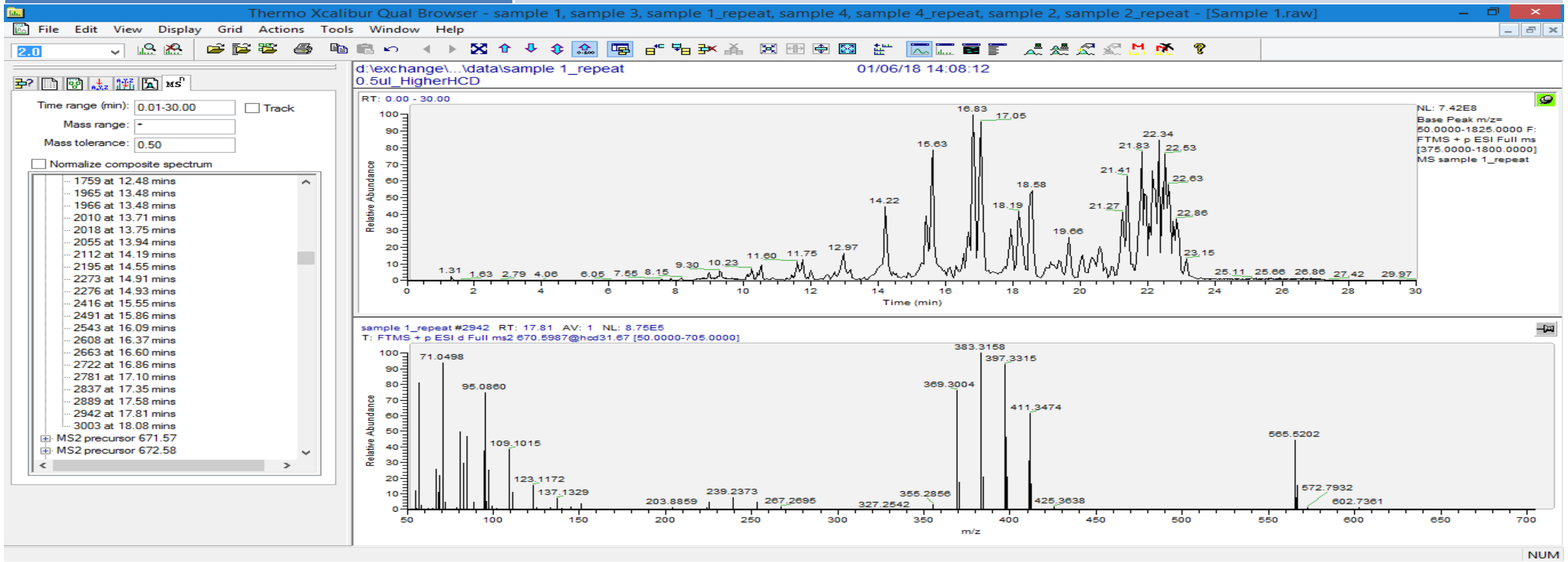

Figure S8 (17): An example MS/MS spectrum of TG 4:0_14:0_17:1 that contains 17:1 as one of the fatty acids.

File Name	Sample 1_repeat
Scan #	2237
Retention Time	14.7445
m/z	640.5515
Chemical Composition	[C38H70O6+NH4] 1+
LIPIDMAPS Abbreviation/Common Name	TG(4:0/14:0/17:1(9Z))
Main Class	TAG
Sub Class	Triacylglycerols
Lipid ID	TG(4:0/14:0/17:1(9Z))
Theoretical m/z	640.5511
Delta Mass	0.6079 ppm
Group	TG
LSI ID	TG 4:0_14:0_17:1
Short Name	TG 35:1
Graded ID	TG 4:0_14:0_17:1
Matched Ions	3:0 C=O+_71.0497(102612.7031), 13:0 C=O+_211.2061(6576.2056), 16:1 C=O+_251.2368(7618.7022), M-17:1_355.2845(86598.2891), M-14:0_395.3158(63127.3945), M-4:0_535.4722(60143.0078)

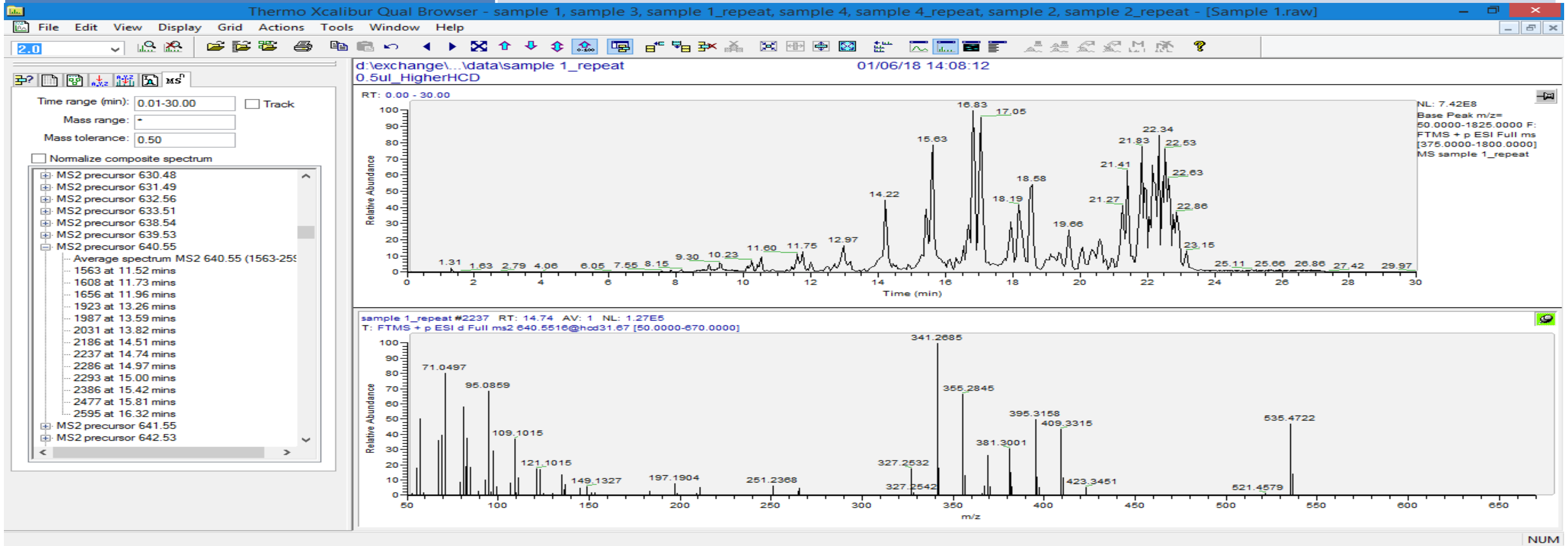

Figure S8 (18): An example MS/MS spectrum of TG 4:0_15:0_18:2 that contains 18:2 as one of the fatty acids.

File Name	Sample 1_repeat
Scan #	2318
Retention Time	15.1265
m/z	666.5671
Chemical Composition	[C40H72O6+NH4] 1+
LIPIDMAPS Abbreviation/Common Name	TG(4:0/15:0/18:2(9Z,11Z))
Main Class	TAG
Sub Class	Triacylglycerols
Lipid ID	TG(4:0/15:0/18:2(9Z,11Z))
Theoretical m/z	666.5667
Delta Mass	0.5466 ppm
Group	TG
LSI ID	TG 4:0_15:0_18:2
Short Name	TG 37:2
Graded ID	TG 4:0_15:0_18:2
Matched Ions	3:0 C=O+_71.0497(33464.0977), 14:0 C=O+_225.2209(3060.1704), 17:2 C=O+_263.2379(7690.2871), M-18:2_369.3002(59638.3008), M-15:0_407.3155(37364.0117), M-4:0_561.4876(11652.0967)

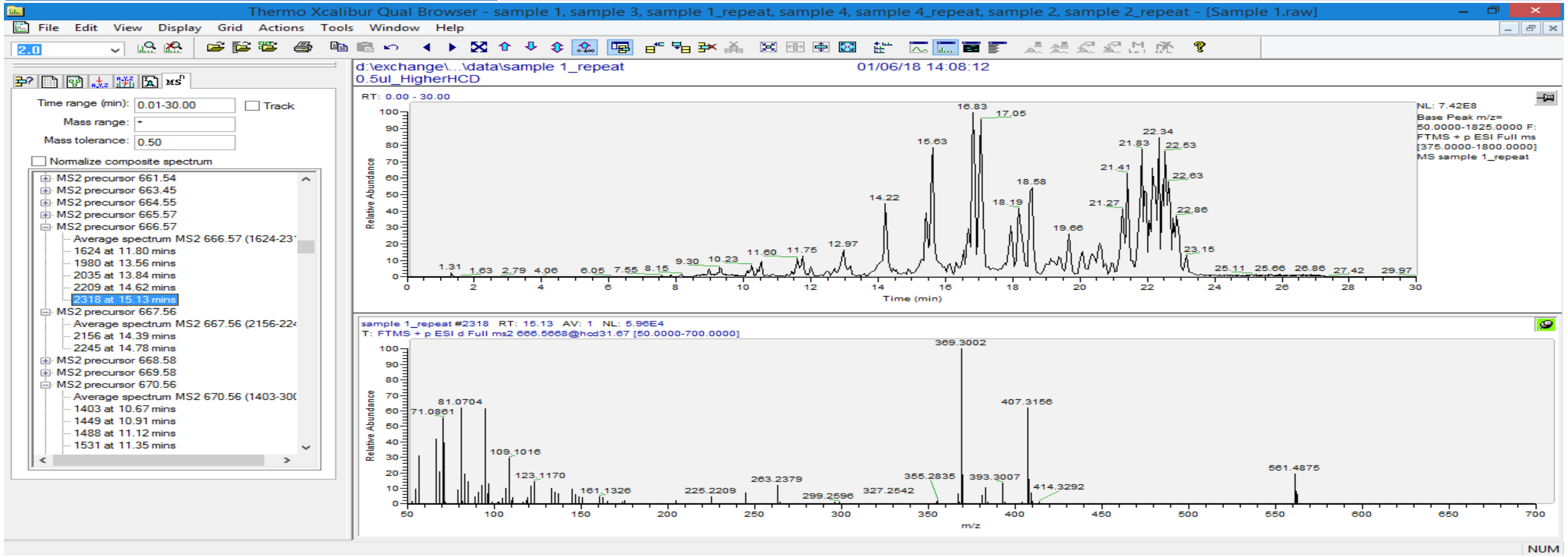

Figure S8 (19): An example MS/MS spectrum of TG 4:0_16:0_18:3 that contains 18:3 as one of the fatty acids.

File Name	Sample 1
Scan #	2226
Retention Time	14.7052
m/z	678.567
Chemical Composition	[C41H72O6+NH4] 1+
LIPIDMAPS Abbreviation/Common Name	TG(4:0/16:0/18:3(6Z,9Z,12Z))
Main Class	TAG
Sub Class	Triacylglycerols
Lipid ID	TG(4:0/16:0/18:3(6Z,9Z,12Z))
Theoretical m/z	678.5667
Delta Mass	0.447 ppm
Group	TG
LSI ID	TG 4:0_16:0_18:3
Short Name	TG 38:3
Graded ID	TG 4:0_16:0_18:3
Matched Ions	3:0 C=O+_71.0497(91287.6016), 15:0 C=O+_239.2369(8421.4502), 17:3 C=O+_261.2213(41331.0664), M-18:3_383.316(102249.8906), M-16:0_405.3(66590.4922), M-4:0_573.4893(12655.7861)

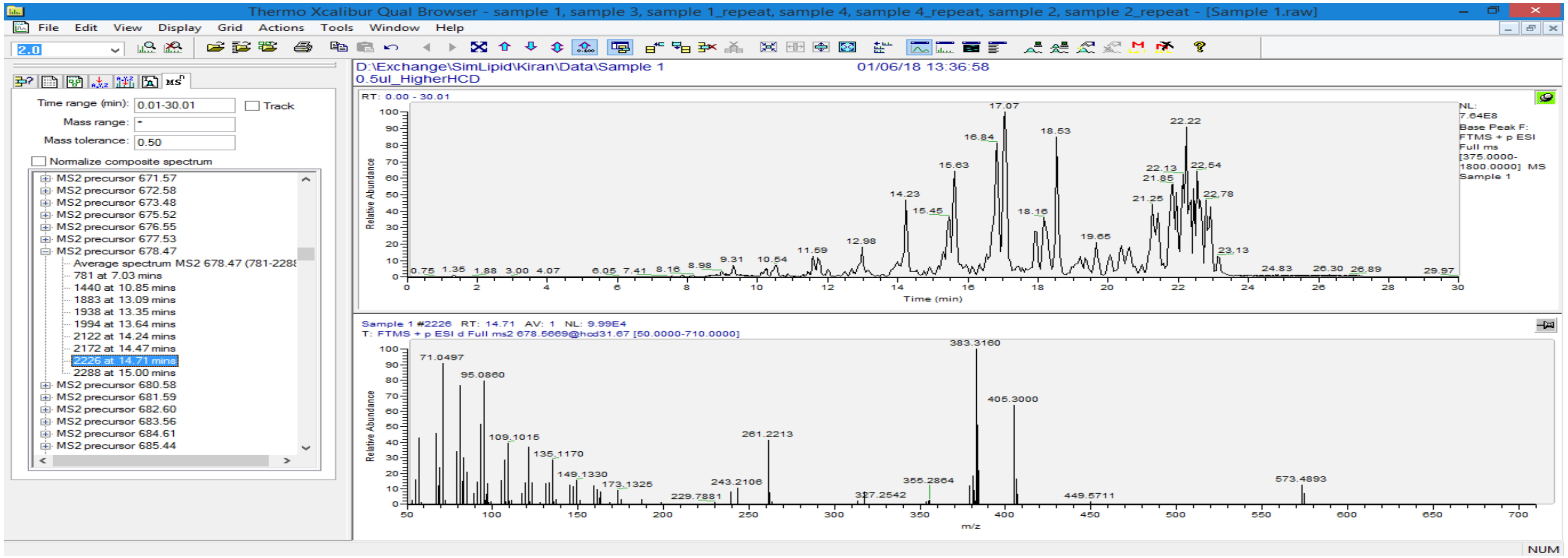

Figure S8 (20): An example MS/MS spectrum of TG 4:0_16:0_19:0 that contains 19:0 as one of the fatty acids.

File Name	Sample 1
Scan #	3272
Retention Time	19.3131
m/z	698.6301
Chemical Composition	[C42H80O6+NH4] 1+
LIPIDMAPS Abbreviation/Common Name	TG(4:0/16:0/19:0)
Main Class	TAG
Sub Class	Triacylglycerols
Lipid ID	TG(4:0/16:0/19:0)
Theoretical m/z	698.6293
Delta Mass	1.0769 ppm
Group	TG
LSI ID	TG 4:0_16:0_19:0
Short Name	TG 39:0
Graded ID	TG 4:0_16:0_19:0
Matched Ions	3:0 C=O+_71.0498(190325.5938), M-19:0_383.3156(108369.9609), M-16:0_425.3632(63992.7344), M-4:0_593.5515(71912.3594)

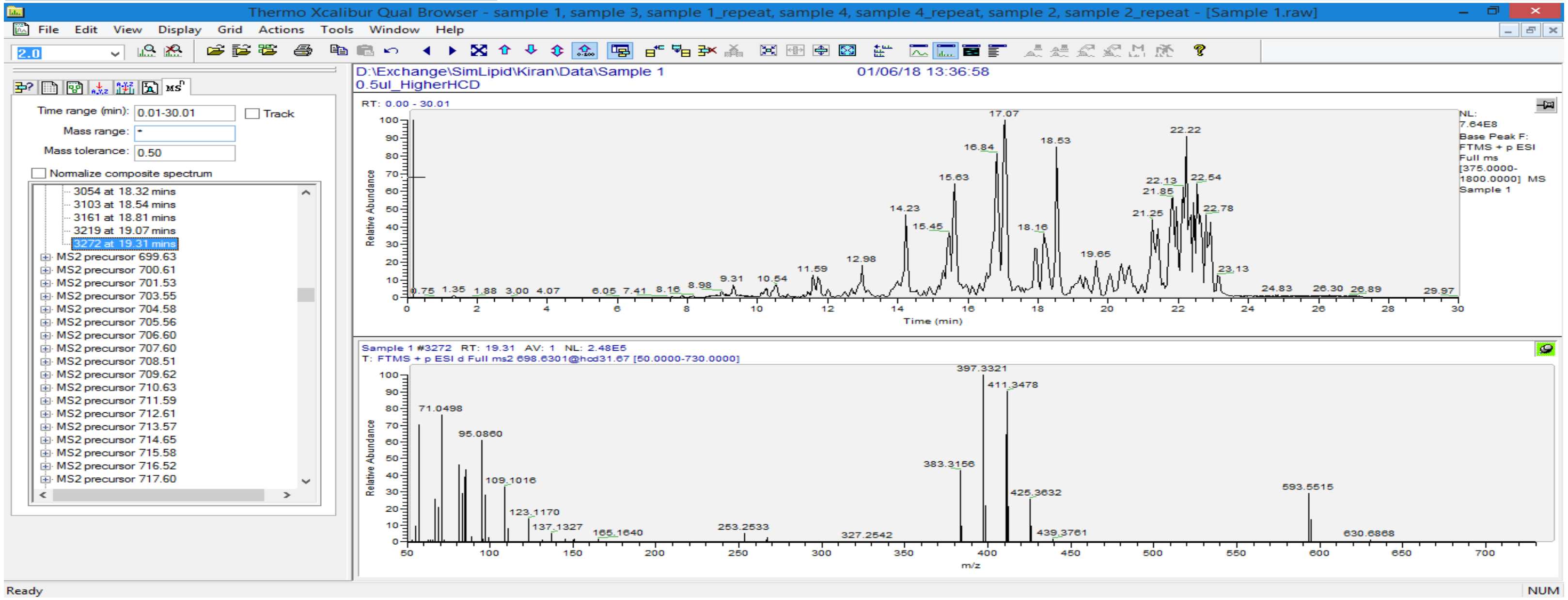

Figure S8 (21): An example MS/MS spectrum of TG 4:0_18:0_19:1 that contains 19:1 as one of the fatty acids.

File Name	Sample 1_repeat
Scan #	3193
Retention Time	18.9415
m/z	724.6453
Chemical Composition	[C44H82O6+NH4] 1+
LIPIDMAPS Abbreviation/Common Name	TG(4:0/18:0/19:1(9Z))
Main Class	TAG
Sub Class	Triacylglycerols
Lipid ID	TG(4:0/18:0/19:1(9Z))
Theoretical m/z	724.645
Delta Mass	0.4983 ppm
Group	TG
LSI ID	TG 4:0_18:0_19:1
Short Name	TG 41:1
Graded ID	TG 4:0_18:0_19:1
Matched Ions	3:0 C=O+_71.0497(44013.2617), M-19:1_411.3472(40900.5547), M-18:0_423.3464(18980.3867), M-4:0_619.5675(12176.5889)

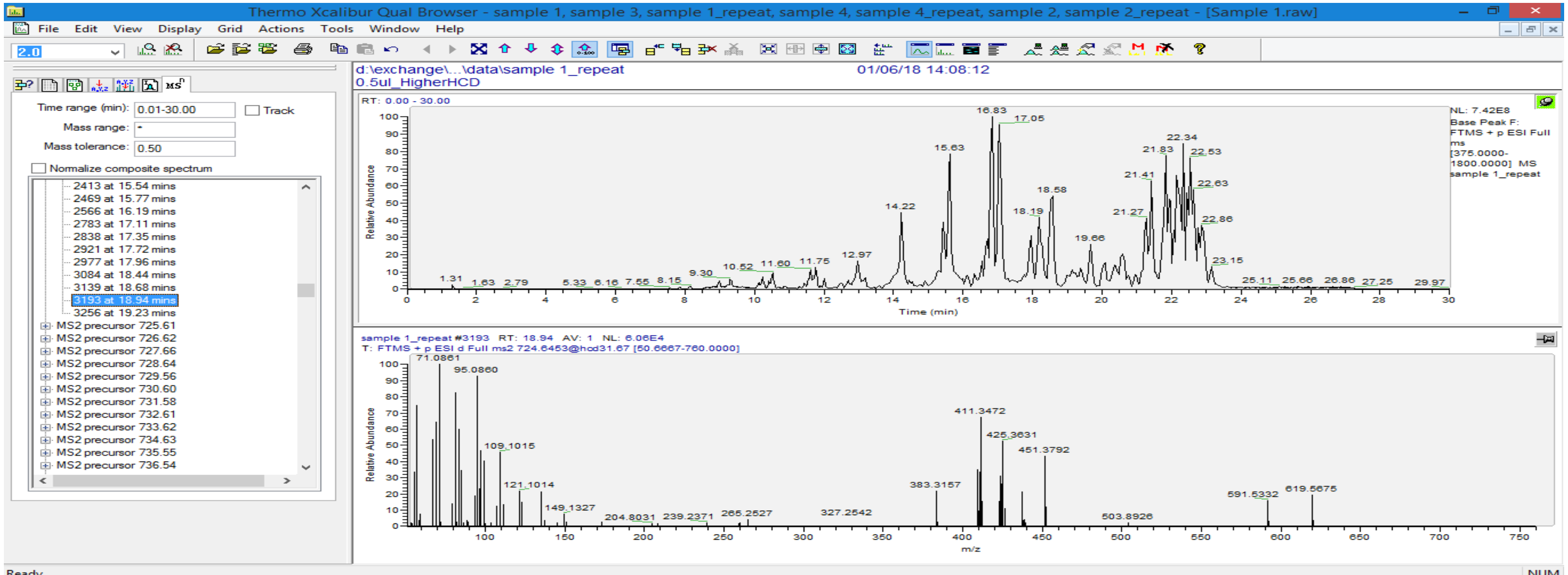

Figure S8 (22): An example MS/MS spectrum of TG 4:0_16:0_20:0 that contains 20:0 as one of the fatty acids.

File Name	Sample 2_repeat
Scan #	3368
Retention Time	19.9946
m/z	712.6451
Chemical Composition	[C43H82O6+NH4] 1+
LIPIDMAPS Abbreviation/Common Name	TG(4:0/16:0/20:0)
Main Class	TAG
Sub Class	Triacylglycerols
Lipid ID	TG(4:0/16:0/20:0)
Theoretical m/z	712.645
Delta Mass	0.1641 ppm
Group	TG
LSI ID	TG 4:0_16:0_20:0
Short Name	TG 40:0
Graded ID	TG 4:0_16:0_20:0
Matched Ions	3:0 C=O+_71.0497(260969.75), 15:0 C=O+_239.2363(6131.1621), M-20:0_383.3155(131044.0078), M-16:0_439.3784(143515.5781), M-4:0_607.5667(92499.6875)

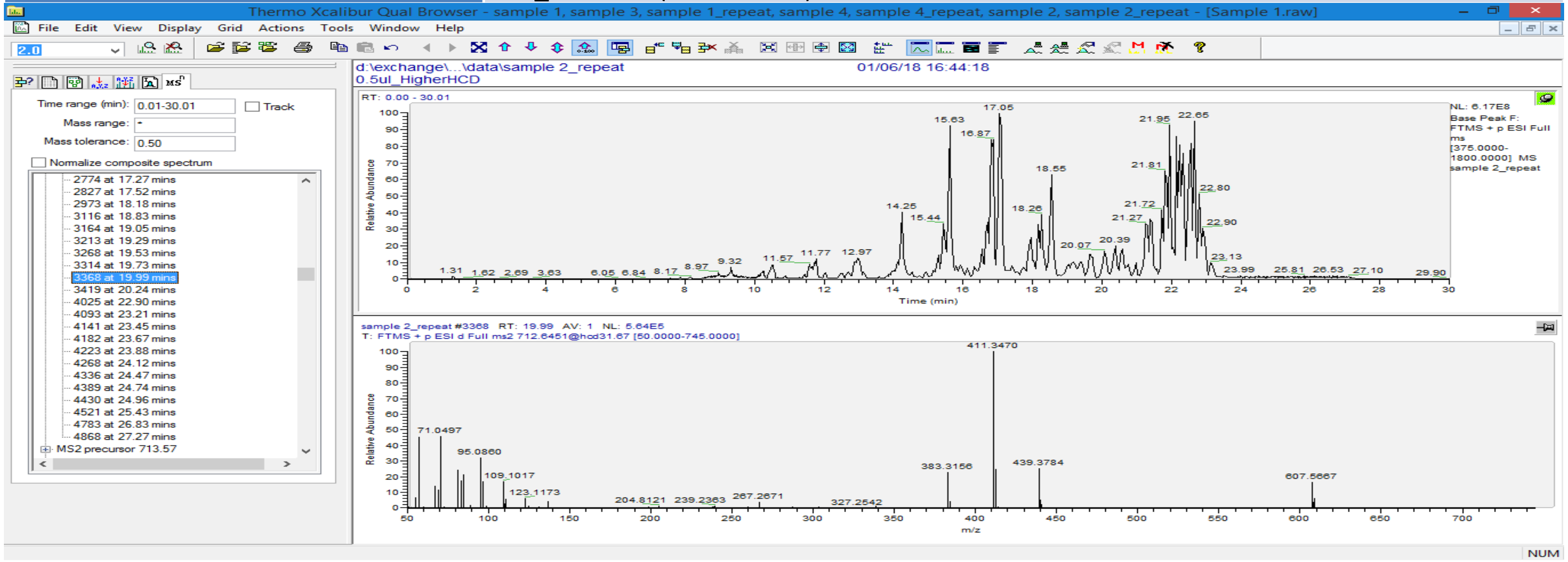

Figure S8 (23): An example MS/MS spectrum of TG 16:0_20:1_26:0 that contains the fatty acids 20:1 and 26:0.

File Name	Sample 1
Scan #	4205
Retention Time	23.4186
m/z	1018.9739
Chemical Composition	[C65H124O6+NH4] 1+
LIPIDMAPS Abbreviation/Common Name	TG(16:0/20:1(11Z)/26:0)
Main Class	TAG
Sub Class	Triacylglycerols
Lipid ID	TG(16:0/20:1(11Z)/26:0)
Theoretical m/z	1018.9736
Delta Mass	0.29445 ppm
Group	TG
LSI ID	TG 16:0_20:1_26:0
Short Name	TG 62:1
Graded ID	TG 16:0_20:1_26:0
Matched Ions	15:0 C=O+_239.2359(2326.6721), M-26:0_605.5496(24584.4082), M-20:1_691.6559(994.5347), M-16:0_745.7075(21694.2832)

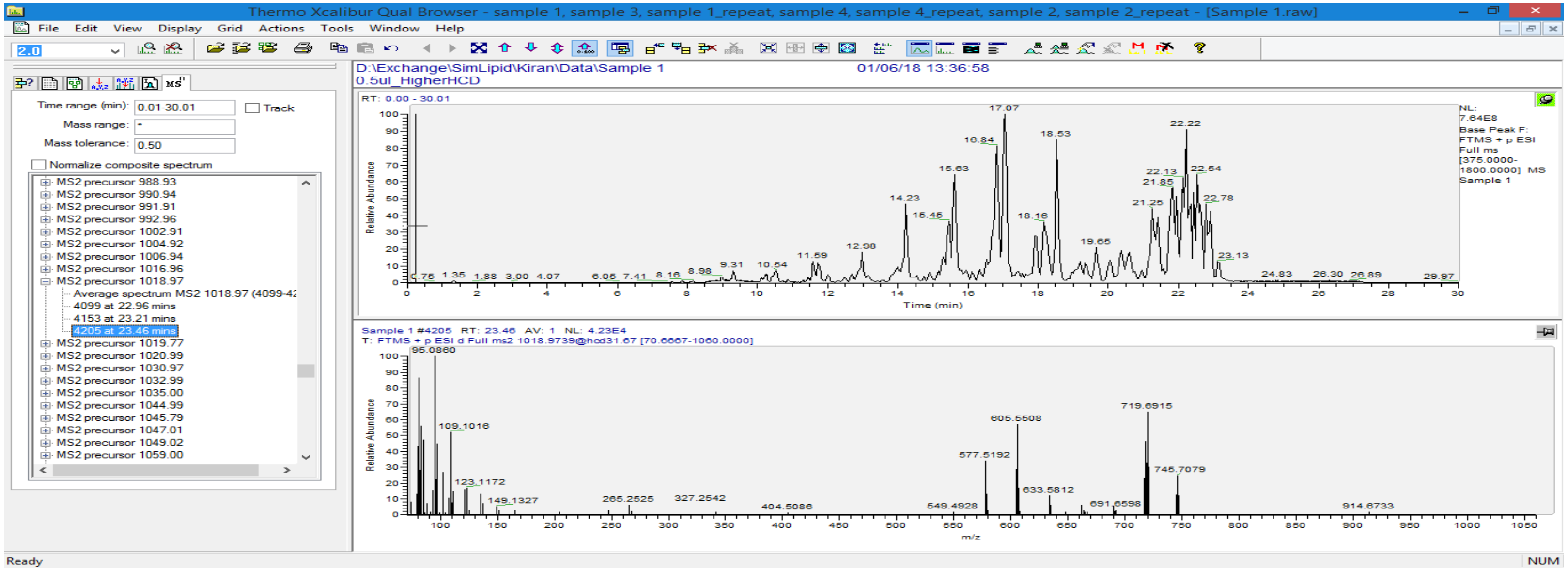

Figure S8 (24): An example MS/MS spectrum of TG 8:0_14:0_20:2 that contains 20:2 as one of the fatty acids.

File Name	Sample 1
Scan #	2977
Retention Time	17.9829
m/z	736.6453
Chemical Composition	[C45H82O6+NH4] 1+
LIPIDMAPS Abbreviation/Common Name	TG(8:0/14:0/20:2(11Z,14Z))
Main Class	TAG
Sub Class	Triacylglycerols
Lipid ID	TG(8:0/14:0/20:2(11Z,14Z))
Theoretical m/z	736.645
Delta Mass	0.4073 ppm
Group	TG
LSI ID	TG 8:0_14:0_20:2
Short Name	TG 42:2
Graded ID	TG 8:0_14:0_20:2
Matched Ions	7:0 C=O+_127.1121(51107.6445), M-20:2_411.3479(7207.793), M-14:0_491.4098(9684.3721), M-8:0_575.5034(38560.1016)

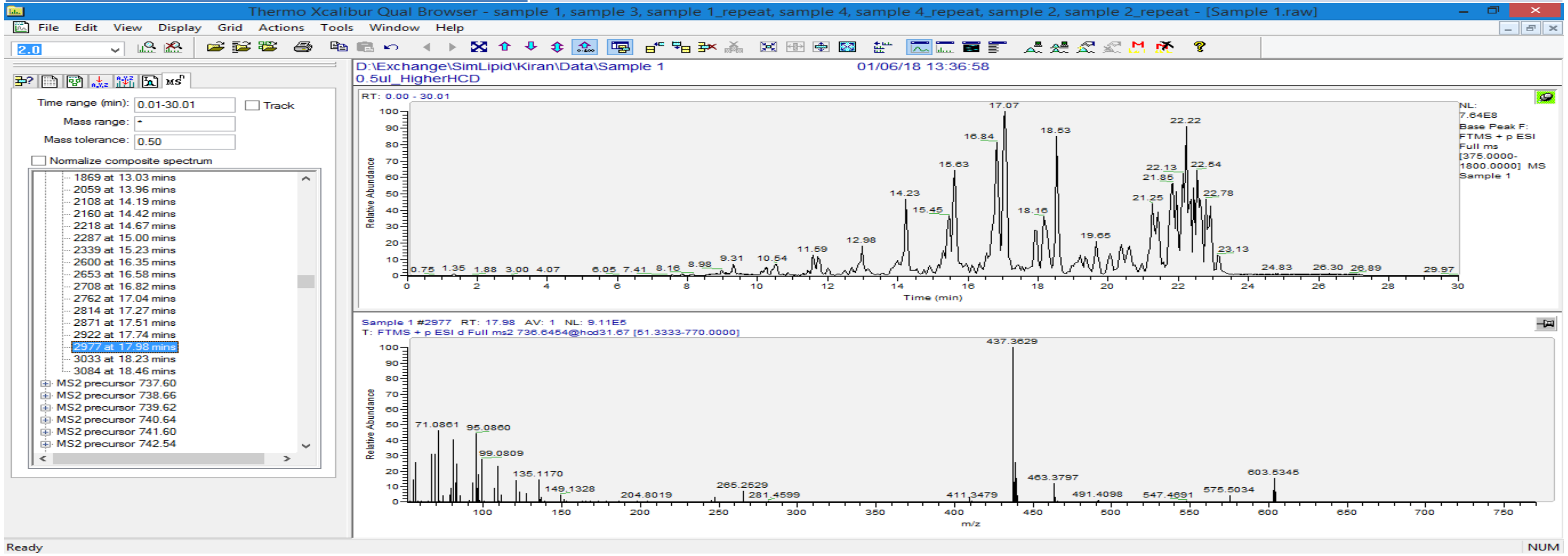

Figure S8 (25): An example MS/MS spectrum of TG 4:0_18:0_20:3 that contains 20:3 as one of the fatty acids.

File Name	Sample 1_repeat
Scan #	2827
Retention Time	17.3034
m/z	734.6293
Chemical Composition	[C45H80O6+NH4] 1+
LIPIDMAPS Abbreviation/Common Name	TG(4:0/18:0/20:3(8Z,11Z,14Z))
Main Class	TAG
Sub Class	Triacylglycerols
Lipid ID	TG(4:0/18:0/20:3(8Z,11Z,14Z))
Theoretical m/z	734.6293
Delta Mass	0.0271 ppm
Group	TG
LSI ID	TG 4:0_18:0_20:3
Short Name	TG 42:3
Graded ID	TG 4:0_18:0_20:3
Matched Ions	3:0 C=O+_71.0498(11990.4131), 17:0 C=O+_267.2691(552.5635), 19:3 C=O+_289.2525(7000.7554), M-20:3_411.348(16662.8379), M-18:0_433.332(13723.9824), M-4:0_629.5464(2125.3621)

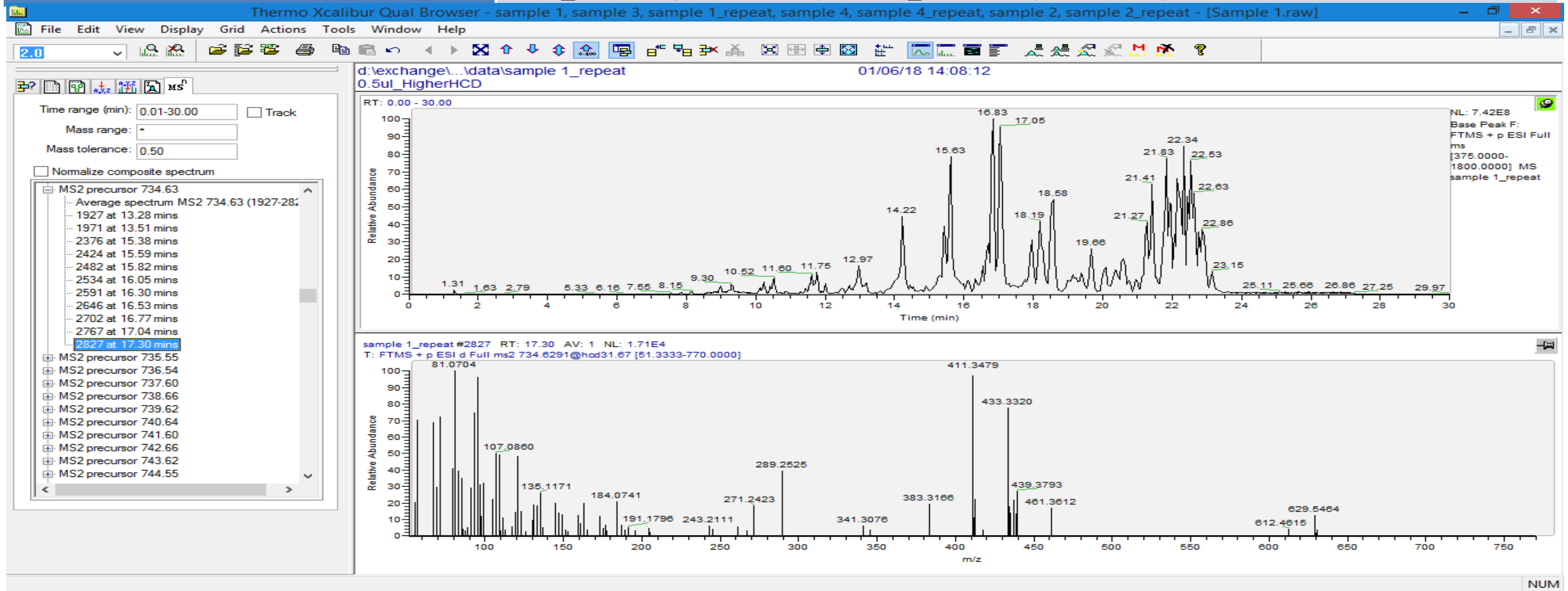

Figure S8 (26): An example MS/MS spectrum of TG 4:0_16:0_20:4 that contains 20:4 as one of the fatty acids.

File Name	Sample 2
Scan #	2299
Retention Time	15.25
m/z	704.5824
Chemical Composition	[C43H74O6+NH4] 1+
LIPIDMAPS Abbreviation/Common Name	TG(4:0/16:0/20:4(7Z,9Z,11Z,13Z))
Main Class	TAG
Sub Class	Triacylglycerols
Lipid ID	TG(4:0/16:0/20:4(7Z,9Z,11Z,13Z))
Theoretical m/z	704.582338
Delta Mass	0.088 ppm
Group	TG
LSI ID	TG 4:0_16:0_20:4
Short Name	TG 40:4
Graded ID	TG 4:0_16:0_20:4
Matched Ions	3:0 C=O+_71.0498(24438.962891), 15:0 C=O+_239.23749(2511.0979), 19:4 C=O+_287.23749(6816.102539), M-16:0_431.313586(5204.214355), M-20:4_383.31785(40236.30469), M-4:0_599.5023(799.368835)

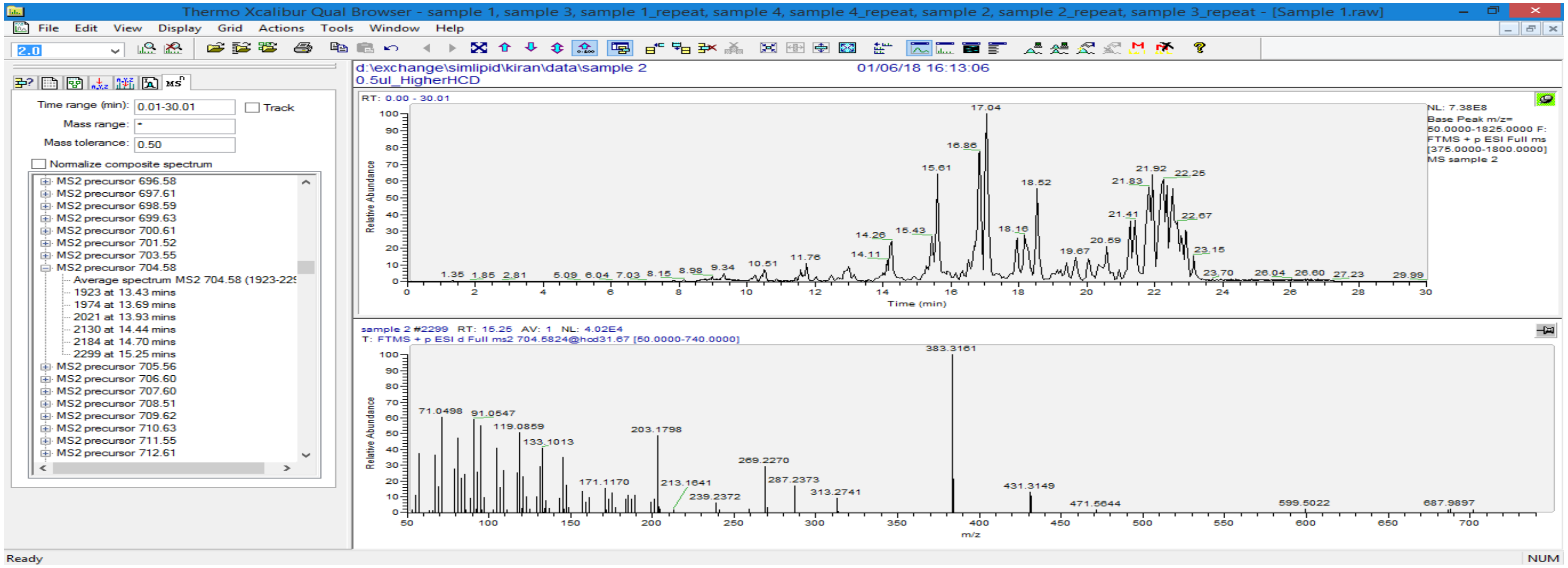

Figure S8 (27): An example MS/MS spectrum of TG 4:0_16:0_21:0 that contains 21:0 as one of the fatty acids.

File Name	Sample 1_repeat
Scan #	3569
Retention Time	20.6611
m/z	726.6613
Chemical Composition	[C44H84O6+NH4] 1+
LIPIDMAPS Abbreviation/Common Name	TG(4:0/16:0/21:0)
Main Class	TAG
Sub Class	Triacylglycerols
Lipid ID	TG(4:0/16:0/21:0)
Theoretical m/z	726.6606
Delta Mass	0.8823 ppm
Group	TG
LSI ID	TG 4:0_16:0_21:0
Short Name	TG 41:0
Graded ID	TG 4:0_16:0_21:0
Matched Ions	3:0 C=O+_71.0498(48228.3633), 15:0 C=O+_239.2372(1851.4611), 20:0 C=O+_309.3164(978.4489), M-21:0_383.3159(57547.8633), M-16:0_453.3941(72222.8438), M-4:0_621.5806(14453.2891)

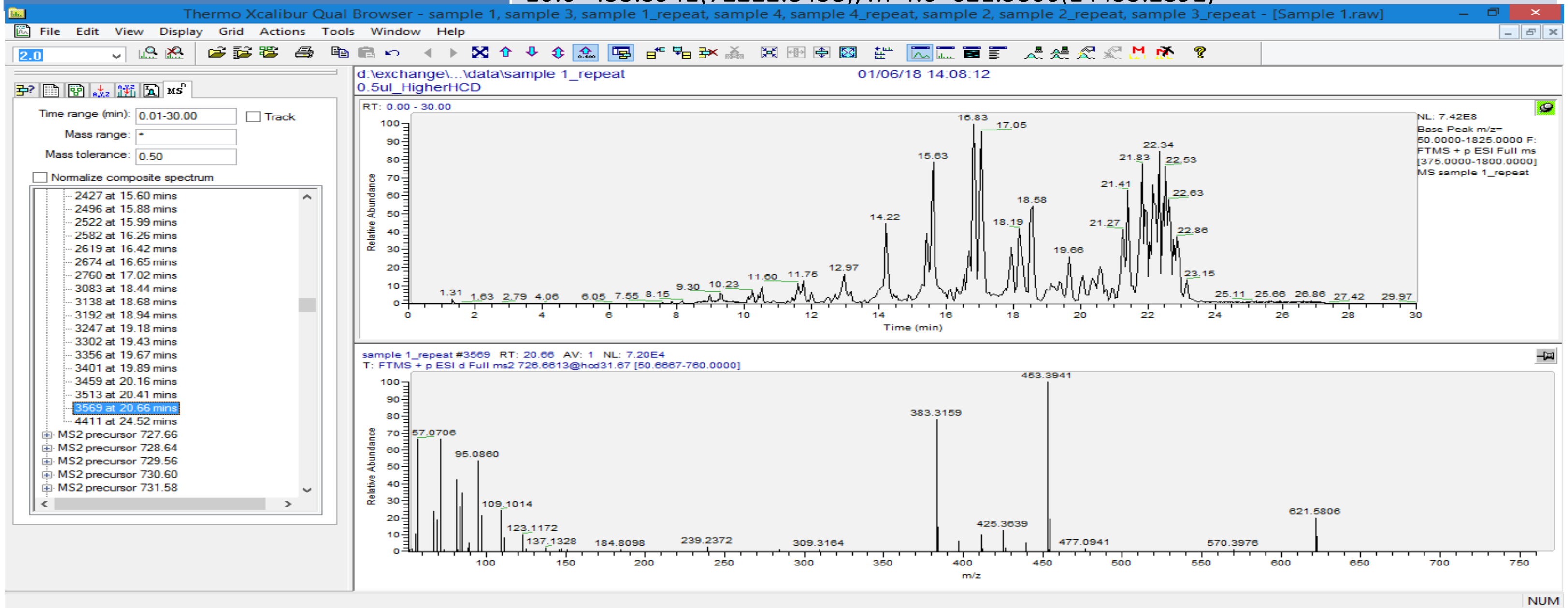

Figure S8 (28): An example MS/MS spectrum of TG 4:0_14:0_22:0 that contains 22:0 as one of the fatty acids.

File Name	Sample 1_repeat
Scan #	3411
Retention Time	19.9319
m/z	712.6456
Chemical Composition	[C43H82O6+NH4] 1+
LIPIDMAPS Abbreviation/Common Name	TG(4:0/14:0/22:0)
Main Class	TAG
Sub Class	Triacylglycerols
Lipid ID	TG(4:0/14:0/22:0)
Theoretical m/z	712.645
Delta Mass	0.9349 ppm
Group	TG
LSI ID	TG 4:0_14:0_22:0
Short Name	TG 40:0
Graded ID	TG 4:0_14:0_22:0
Matched Ions	3:0 C=O+_71.0498(134511.25), M-22:0_355.2833(8715.5381), M-14:0_467.408(8956.5488), M-4:0_607.5665(54056.0273)

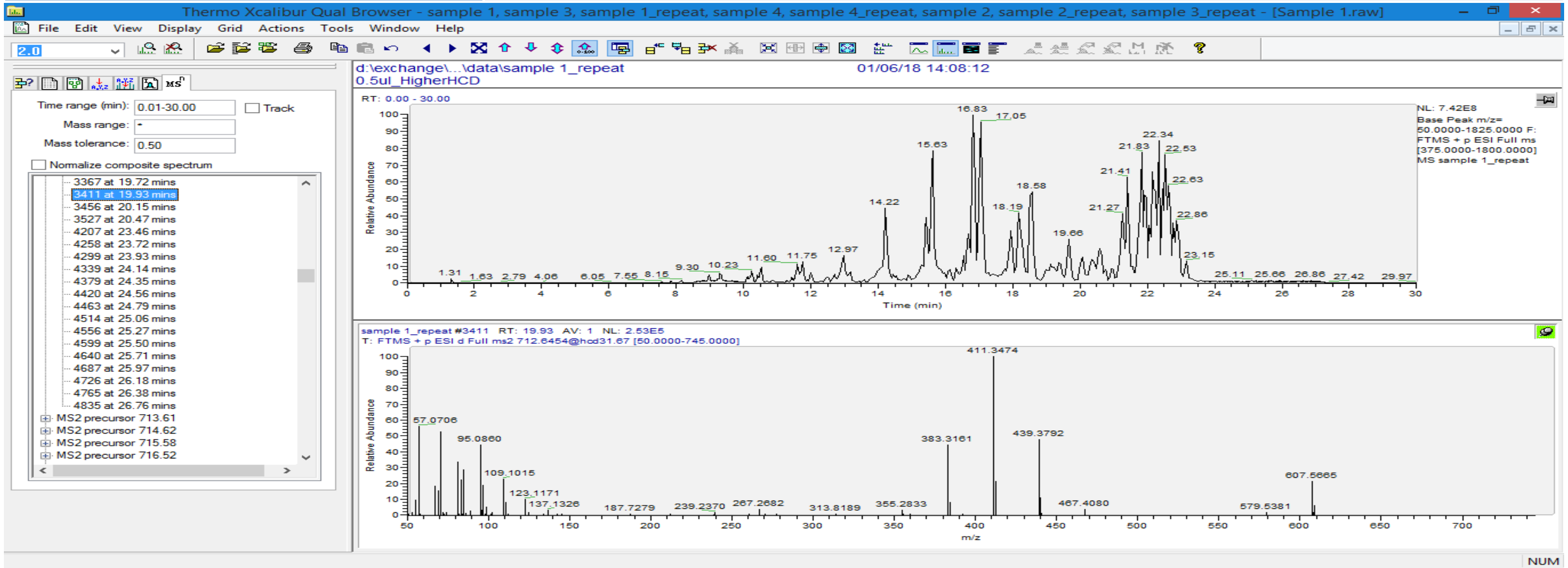

Figure S8 (29): An example MS/MS spectrum of TG 16:0_22:1_24:0 that contains 24:0 as one of the fatty acids.

File Name	Sample 4_repeat
Scan #	4209
Retention Time	23.4404
m/z	1018.9741
Chemical Composition	[C65H124O6+NH4] 1+
LIPIDMAPS Abbreviation/Common Name	TG(16:0/22:1(13Z)/24:0)
Main Class	TAG
Sub Class	Triacylglycerols
Lipid ID	TG(16:0/22:1(13Z)/24:0)
Theoretical m/z	1018.9736
Delta Mass	0.4367 ppm
Group	TG
LSI ID	TG 16:0_22:1_24:0
Short Name	TG 62:1
Graded ID	TG 16:0_22:1_24:0
Matched Ions	15:0 C=O+_239.2361(988.7972), M-24:0_633.5803(4540.0498), M-22:1_663.6274(6191.4751), M-16:0_745.7093(7662.4243)

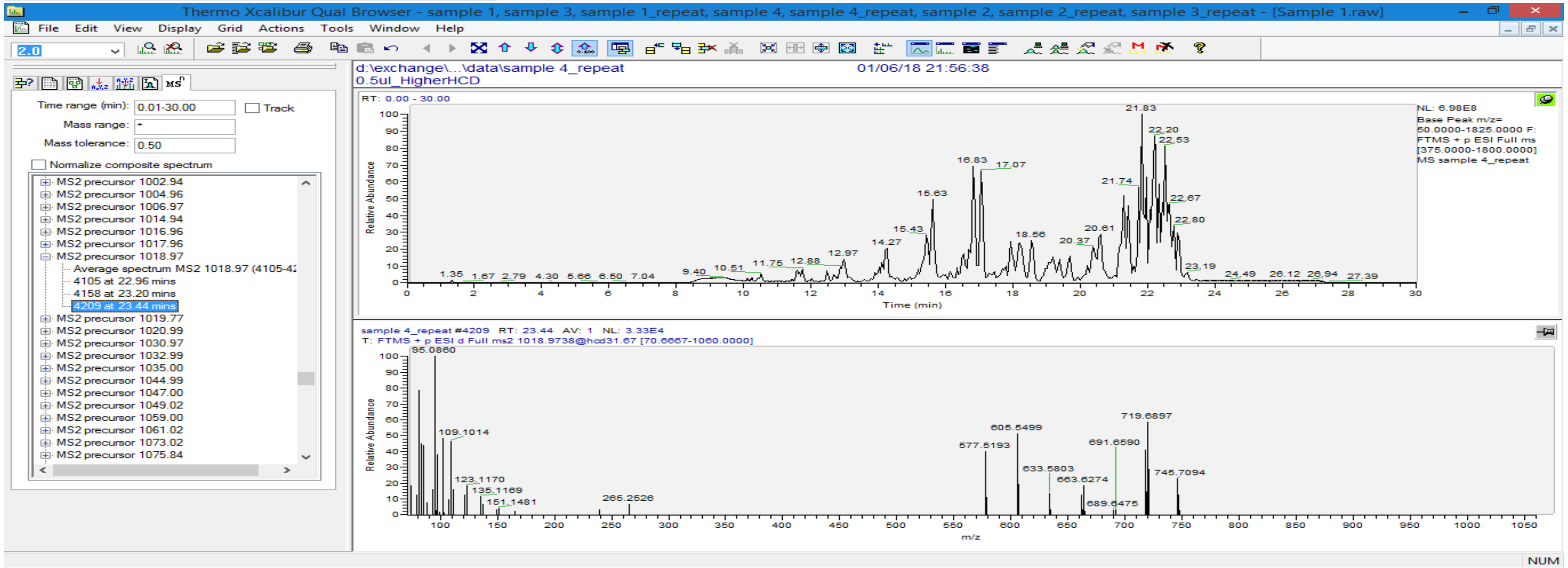

Figure S8 (30): An example MS/MS spectrum of TG 14:0_16:0_22:5 that contains 22:5 as one of the fatty acids.

File Name	Sample 1
Scan #	3663
Retention Time	21.11
m/z	870.7543
Chemical Composition	[C55H96O6+NH4] 1+
LIPIDMAPS Abbreviation/Common Name	TG(14:0/16:0/22:5(6Z,9Z,11Z,13Z,15Z))
Main Class	TAG
Sub Class	Triacylglycerols
Lipid ID	TG(14:0/16:0/22:5(6Z,9Z,11Z,13Z,15Z))
Theoretical m/z	870.754488
Delta Mass	0.2159 ppm
Group	TG
LSI ID	TG 14:0_16:0_22:5
Short Name	TG 52:5
Graded ID	TG 14:0_16:0_22:5
Matched Ions	13:0 C=O+_211.20695(932.551025), M-14:0_625.522354(1104.566895),M-16:0_597.49117(2574.538086),M-22:5_523.475876(5912.317871)

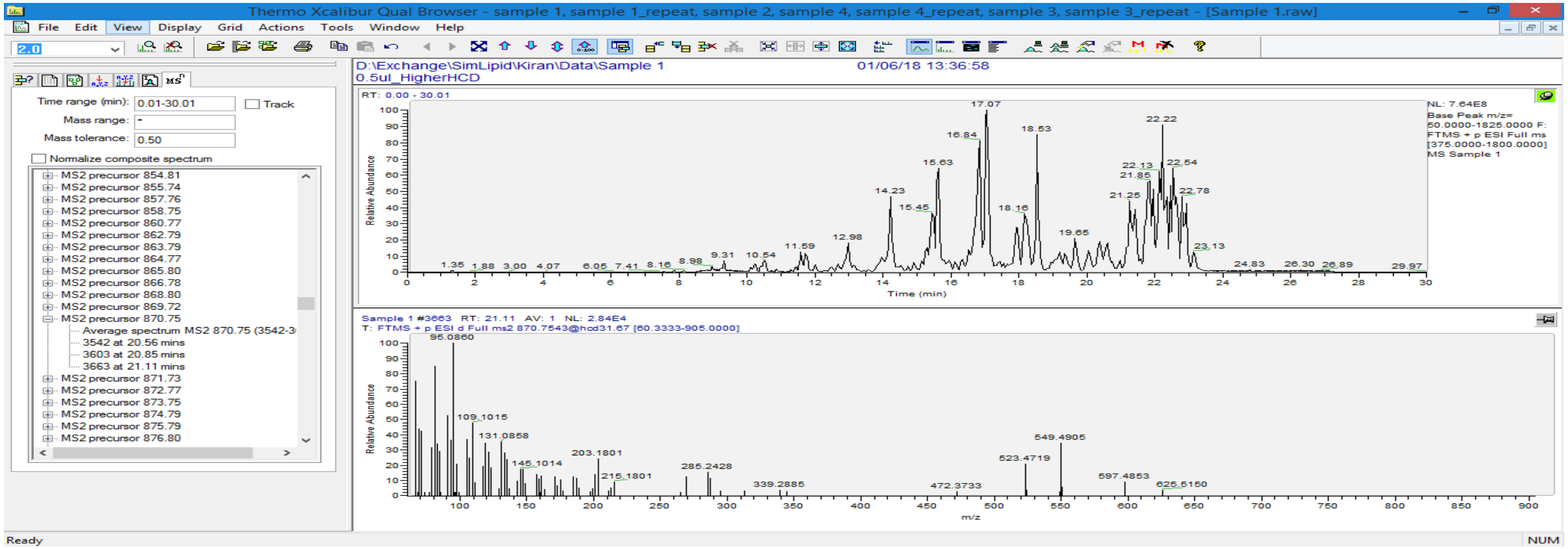

Figure S8 (31): An example MS/MS spectrum of TG 19:0_19:1_23:0 that contains 23:0 as one of the fatty acids.

File Name	Sample 1
Scan #	4188
Retention Time	23.3794
m/z	1004.9576
Chemical Composition	[C64H122O6+NH4] 1+
LIPIDMAPS Abbreviation/Common Name	TG(19:0/19:1(9Z)/23:0)
Main Class	TAG
Sub Class	Triacylglycerols
Lipid ID	TG(19:0/19:1(9Z)/23:0)
Theoretical m/z	1004.958
Delta Mass	0.3826 ppm
Group	TG
LSI ID	TG 19:0_19:1_23:0
Short Name	TG 61:1
Graded ID	TG 19:0_19:1_23:0
Matched Ions	M-23:0_633.577(3553.2925), M-19:1_691.6587(962.2806), M-19:0_706.6772(12579.5098)

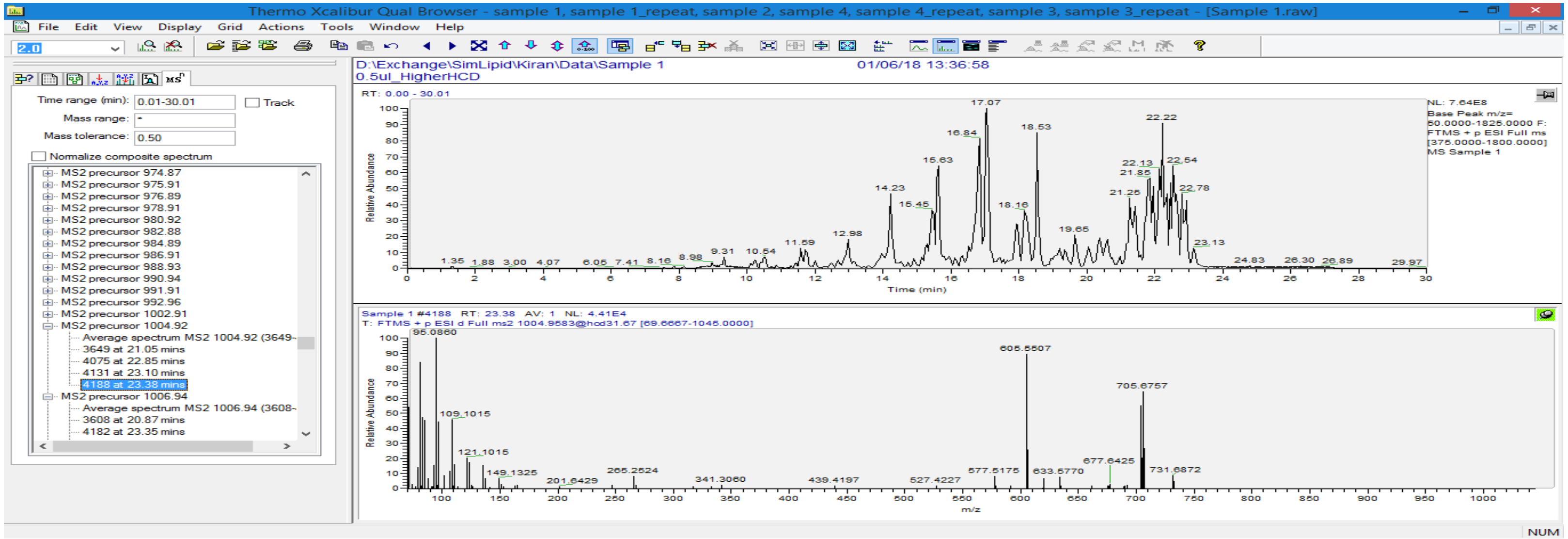

Figure S8 (32): An example MS/MS spectrum of TG 18:0_18:1_23:1 that contains 23:1 as one of the fatty acids.

File Name	Sample 4_repeat
Scan #	4119
Retention Time	23.014
m/z	974.9112
Chemical Composition	[C62H116O6+NH4] 1+
LIPIDMAPS Abbreviation/Common Name	TG(18:0/18:1(9Z)/23:1(15Z))
Main Class	TAG
Sub Class	Triacylglycerols
Lipid ID	TG(18:0/18:1(9Z)/23:1(15Z))
Theoretical m/z	974.911
Delta Mass	0.1836 ppm
Group	TG
LSI ID	TG 18:0_18:1_23:1
Short Name	TG 59:2
Graded ID	TG 18:0_18:1_23:1
Matched Ions	17:1 C=O+_265.2529(6831.1196), M-23:1_605.5506(22797.3203), M-18:0_673.6116(14319.4453), M-18:1_675.6296(77436.2734)

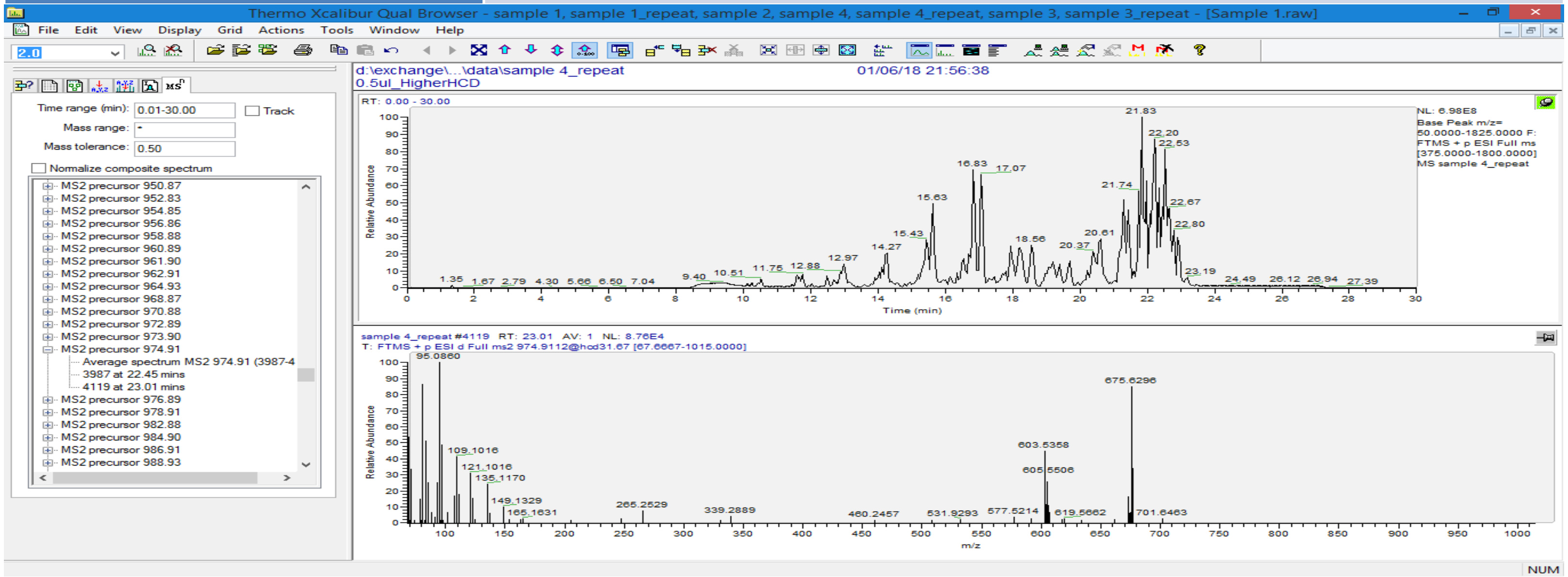

Figure S8 (33): An example MS/MS spectrum of TG 16:0_22:1_24:0 that contains 24:0 as one of the fatty acids.

File Name	Sample 4_repeat
Scan #	4209
Retention Time	23.4404
m/z	1018.9741
Chemical Composition	[C65H124O6+NH4] 1+
LIPIDMAPS Abbreviation/Common Name	TG(16:0/22:1(13Z)/24:0)
Main Class	TAG
Sub Class	Triacylglycerols
Lipid ID	TG(16:0/22:1(13Z)/24:0)
Theoretical m/z	1018.9736
Delta Mass	0.4367 ppm
Group	TG
LSI ID	TG 16:0_22:1_24:0
Short Name	TG 62:1
Graded ID	TG 16:0_22:1_24:0
Matched Ions	15:0 C=O+_239.2361(988.7972), M-24:0_633.5803(4540.0498), M-22:1_663.6274(6191.4751), M-16:0_745.7093(7662.4243)

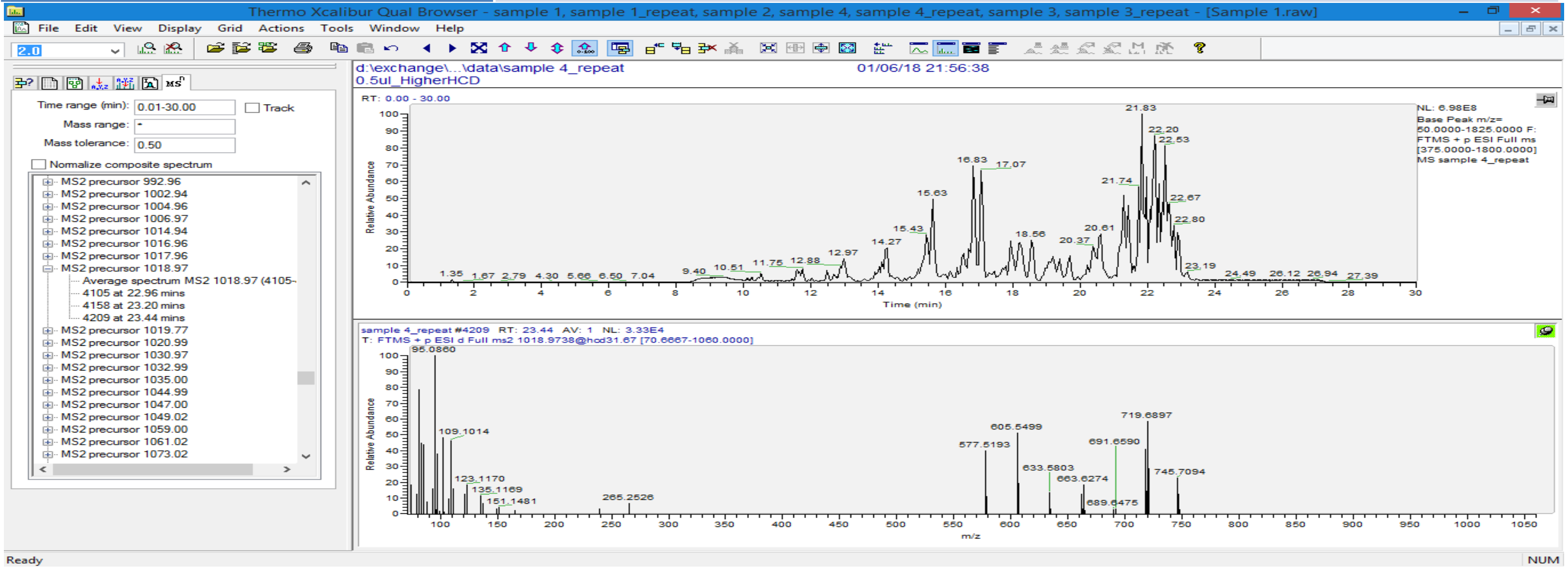

Figure S8 (34): An example MS/MS spectrum of TG TG 18:1_18:1_24:1 that contains 24:1 as one of the fatty acids.

File Name	Sample 4
Scan #	4088
Retention Time	22.8279
m/z	986.9114
Chemical Composition	[C63H116O6+NH4] 1+
LIPIDMAPS Abbreviation/Common Name	TG(18:1(9Z)/18:1(9Z)/24:1(15Z)
Main Class	TAG
Sub Class	Triacylglycerols
Lipid ID	TG(18:1(9Z)/18:1(9Z)/24:1(15Z)
Theoretical m/z	986.911
Delta Mass	0.3669 ppm
Group	TG
LSI ID	TG 18:1_18:1_24:1
Short Name	TG 60:3
Graded ID	TG 18:1_18:1_24:1
Matched Ions	17:1 C=O+_265.2524(6603.5547), 23:1 C=O+_349.3456(2621.0298), M-24:1_603.5355(39123.6328), M-18:1_687.629(58456.4727)

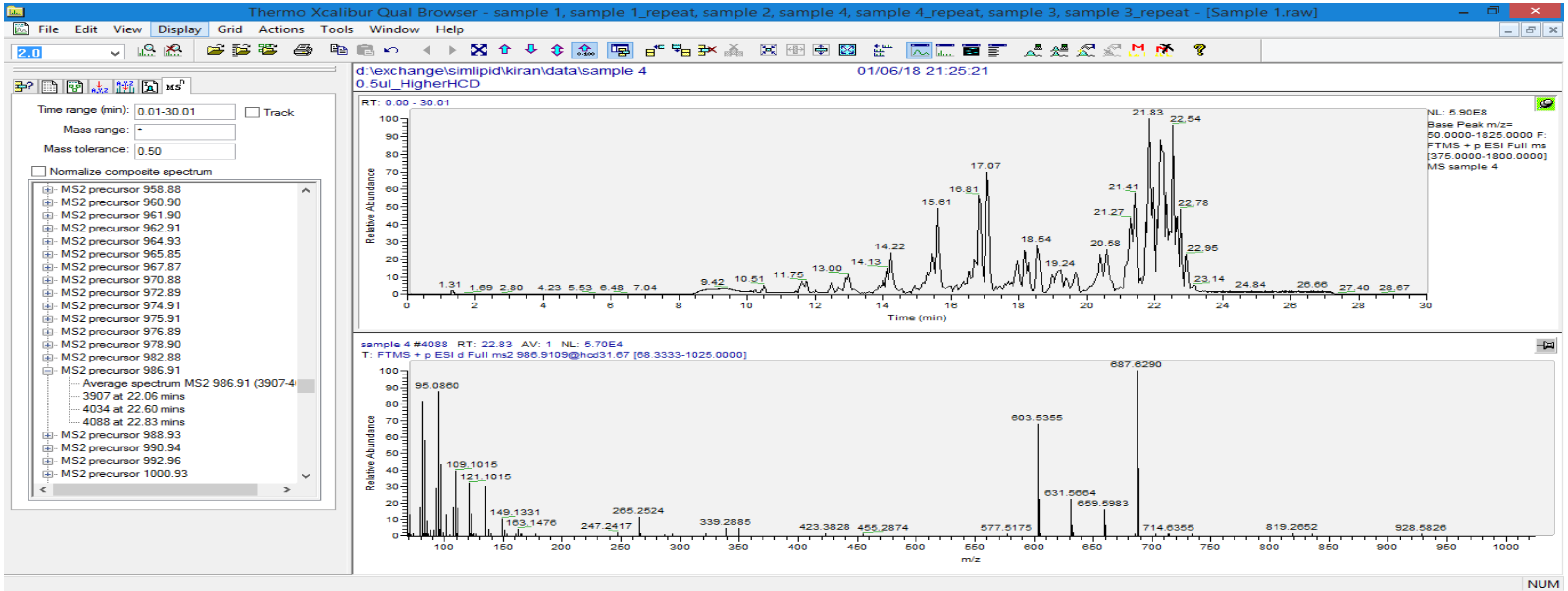

Figure S8 (35): An example MS/MS spectrum of TG 18:1_18:1_25:0 that contains 25:0 as one of the fatty acids.

File Name	Sample 1_repeat
Scan #	4189
Retention Time	23.3722
m/z	1004.9577
Chemical Composition	[C64H122O6+NH4] 1+
LIPIDMAPS Abbreviation/Common Name	TG(18:0/18:1(11Z)/25:0)
Main Class	TAG
Sub Class	Triacylglycerols
Lipid ID	TG(18:0/18:1(11Z)/25:0)
Theoretical m/z	1004.958
Delta Mass	0.2611 ppm
Group	TG
LSI ID	TG 18:0_18:1_25:0
Short Name	TG 61:1
Graded ID	TG 18:0_18:1_25:0
Matched Ions	17:1 C=O+_265.2532(6511.7666), 17:0 C=O+_267.2697(989.1013), M-25:0_605.5505(73100.7109), M-18:0_703.6603(47805.8516), M-18:1_705.6761(52641.707)

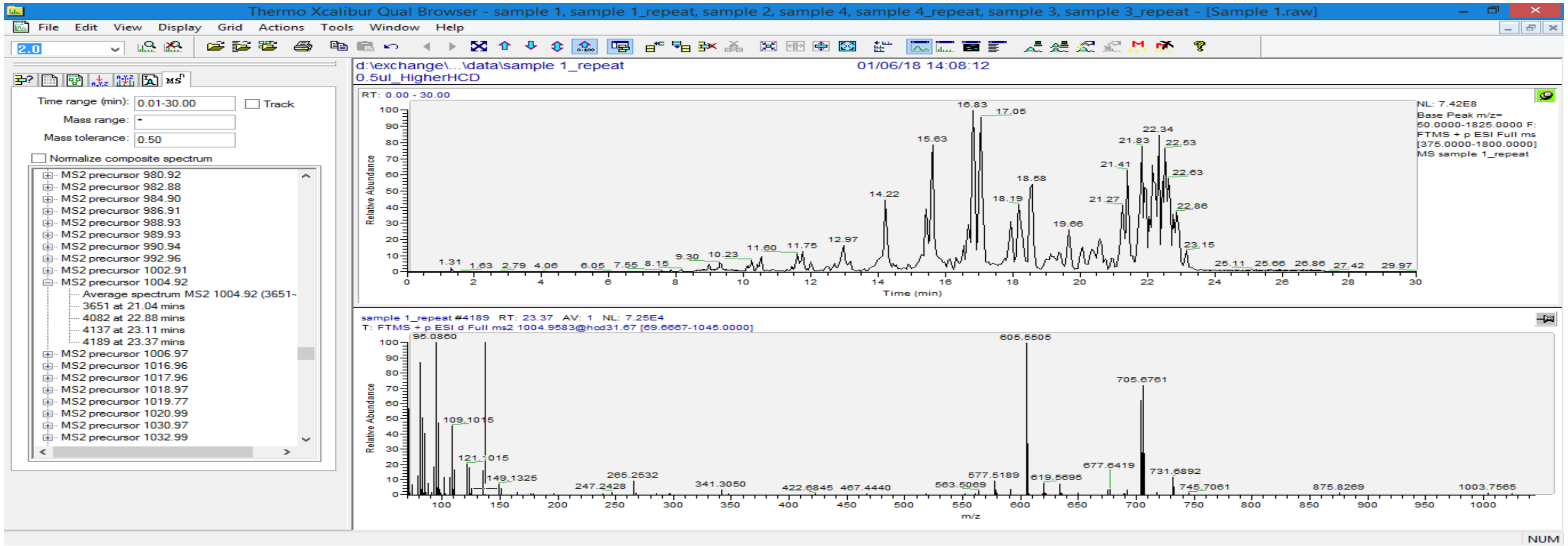

Figure S8 (36): An example MS/MS spectrum of TG 16:0_18:1_28:0 that contains 28:0 as one of the fatty acids.

**File Name**	**Sample 3**
**Scan #**	4061
**Retention Time**	23.4186
**m/z**	1018.9736
**Chemical Composition**	[C65H124O6+NH4] 1+
**LIPIDMAPS Abbreviation/Common Name**	TG(16:0/18:1(11Z)/28:0)
**Main Class**	TAG
**Sub Class**	Triacylglycerols
**Lipid ID**	TG(16:0/18:1(11Z)/28:0)
**Theoretical m/z**	1018.9736
**Delta Mass**	0.0425 ppm
**Group**	TG
**LSI ID**	TG 16:0_18:1_28:0
**Short Name**	TG 62:1
**Graded ID**	TG 16:0_18:1_28:0
**Matched Ions**	15:0 C=O+_239.2359(2326.6721), 17:1 C=O+_265.2522(4266.9341), M-28:0_577.5195(46372.1562), M-18:1_719.6909(42828.0312), M-16:0_745.7075(21694.2832)

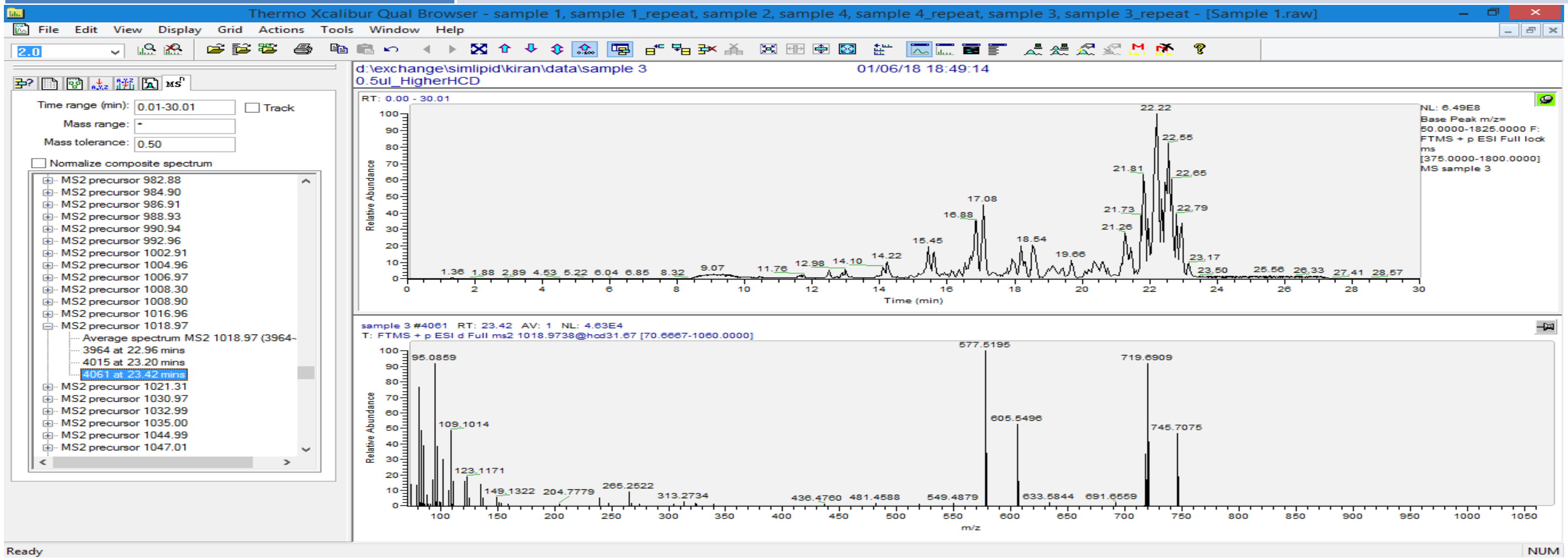

Figure S8 (37): An example MS/MS spectrum of TG 16:0_16:0_30:0 that contains 30:0 as one of the fatty acids.

File Name	Sample 1_repeat
Scan #	4254
Retention Time	23.7006
m/z	1020.9891
Chemical Composition	[C65H126O6+NH4] 1+
LIPIDMAPS Abbreviation/Common Name	TG(16:0/16:0/30:0)
Main Class	TAG
Sub Class	Triacylglycerols
Lipid ID	TG(16:0/16:0/30:0)
Theoretical m/z	1020.9893
Delta Mass	0.1268 ppm
Group	TG
LSI ID	TG 16:0_16:0_30:0
Short Name	TG 62:0
Graded ID	TG 16:0_16:0_30:0
Matched Ions	15:0 C=O+_239.2361(538.6237), M-30:0_551.5041(3648.1958), M-16:0_747.7242(6314.2481)

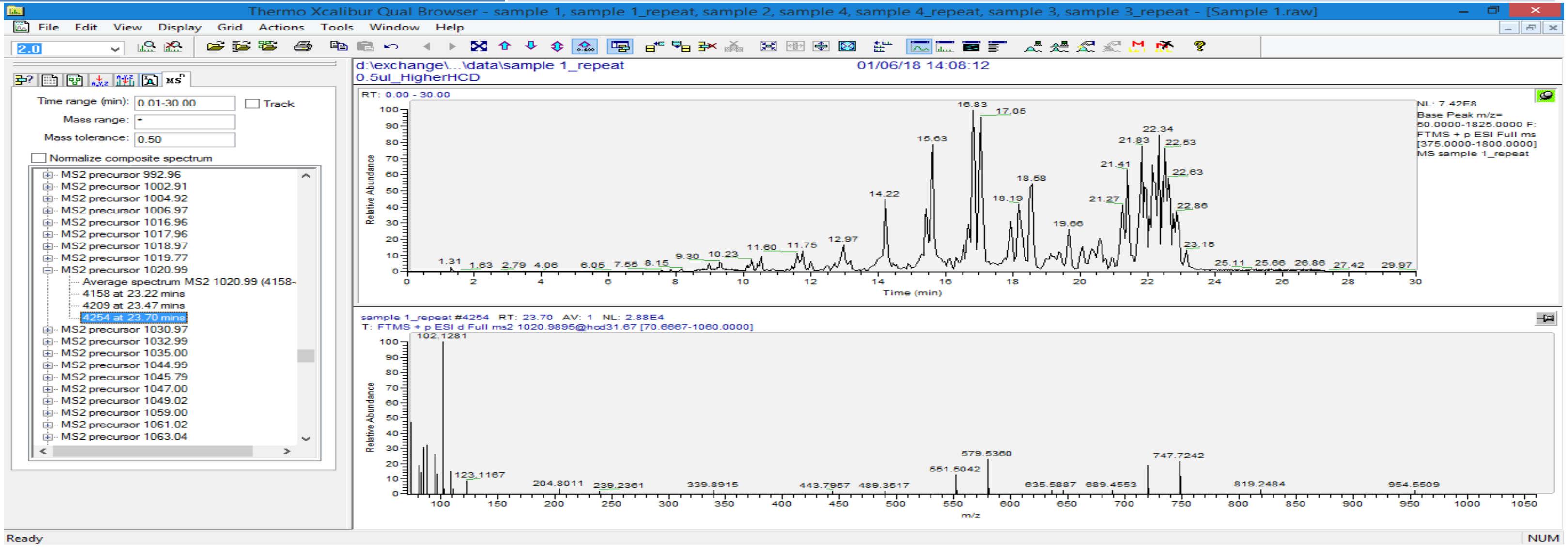

Figure S9: MS2 spectra annotated with neutral loss of fatty acids from the parent ion of the lipid molecular species with Group -ID TG 20:4.

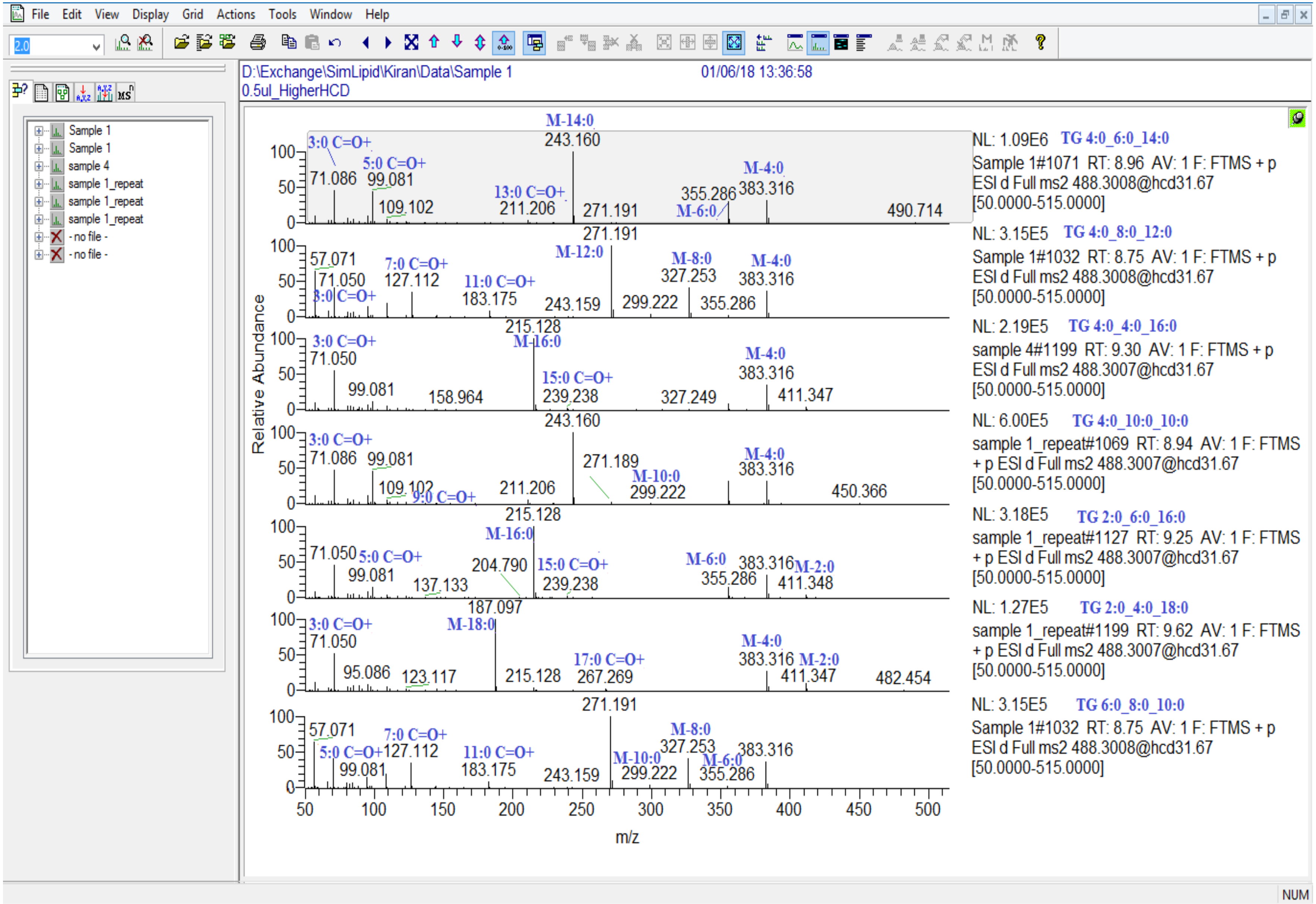

Figure S10: A typical screenshot of Thermo Fisher Scientific's Xcalibur software graphical user interface showing XICs of two molecular ions of the Group-ID TG 40:2 with parent ion masses 656.5829 ([TG 40:2+NH4]$^{+}$), and 661.5376 ([TG 40:2+Na]$^{+}$). The spectra displayed in the lower panel of the graphical user interface were manually stacked for easy data visualization.

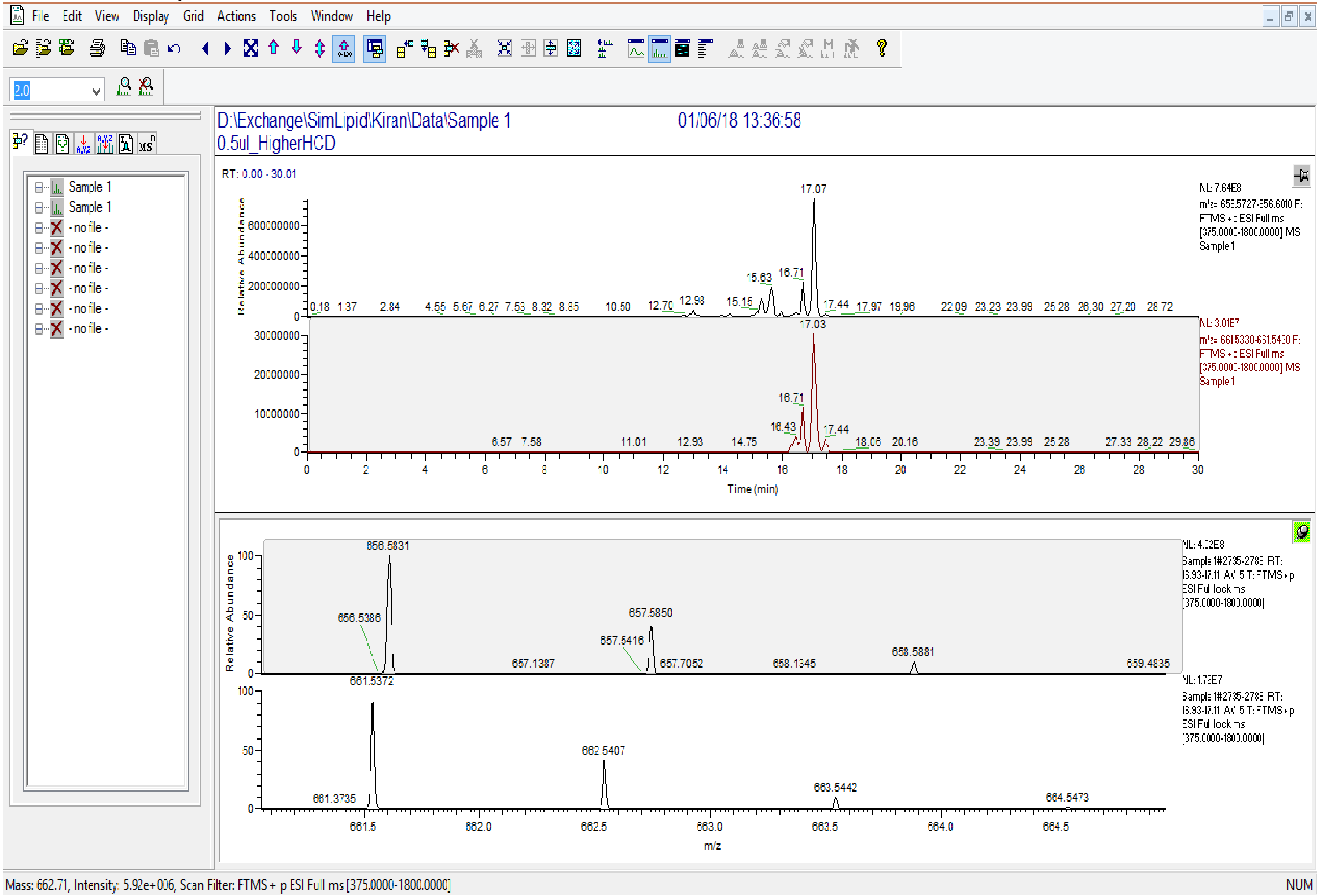

Figure S11(A): Coefficients of variation for 141 TG species

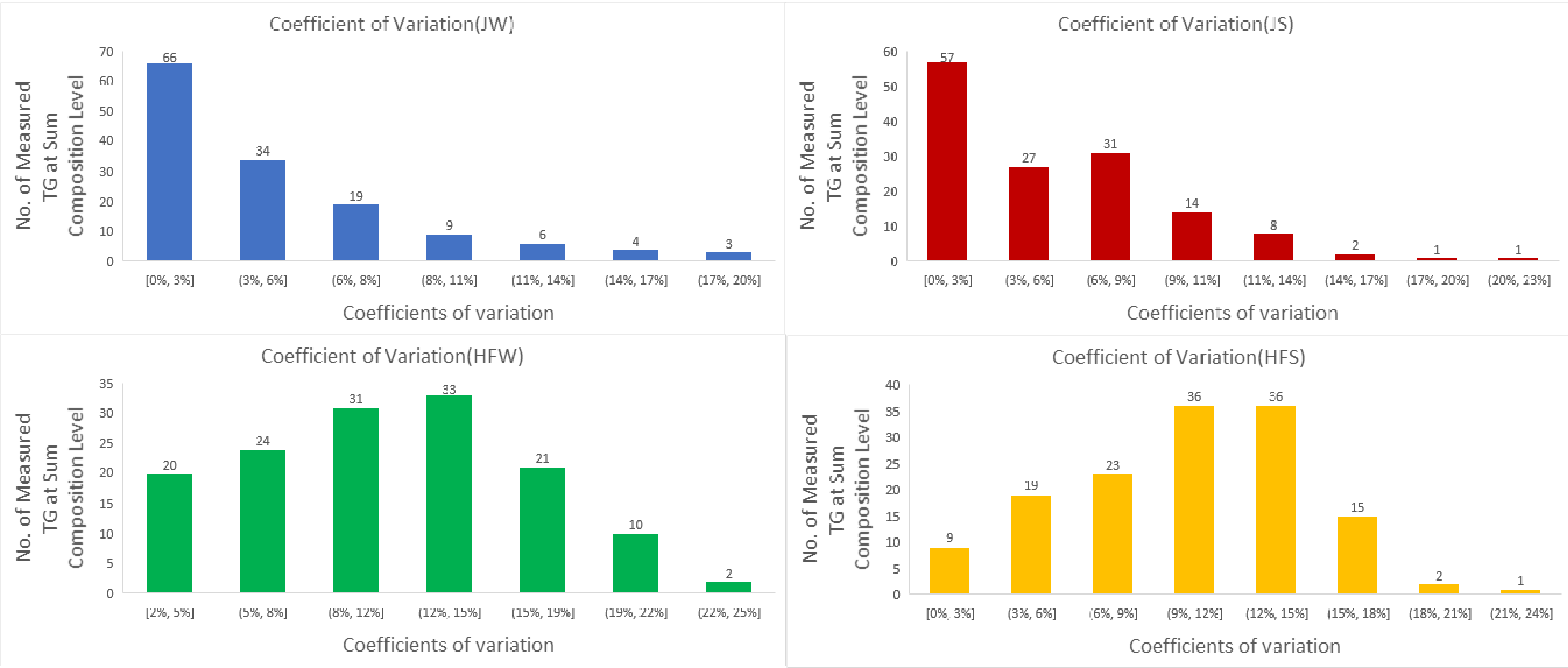

Figure S11(B): 54 other lipid species namely, DG, PC, PE, PI, PS, and Chol Der at sum composition level in JW, JS, HFS, and HFW.

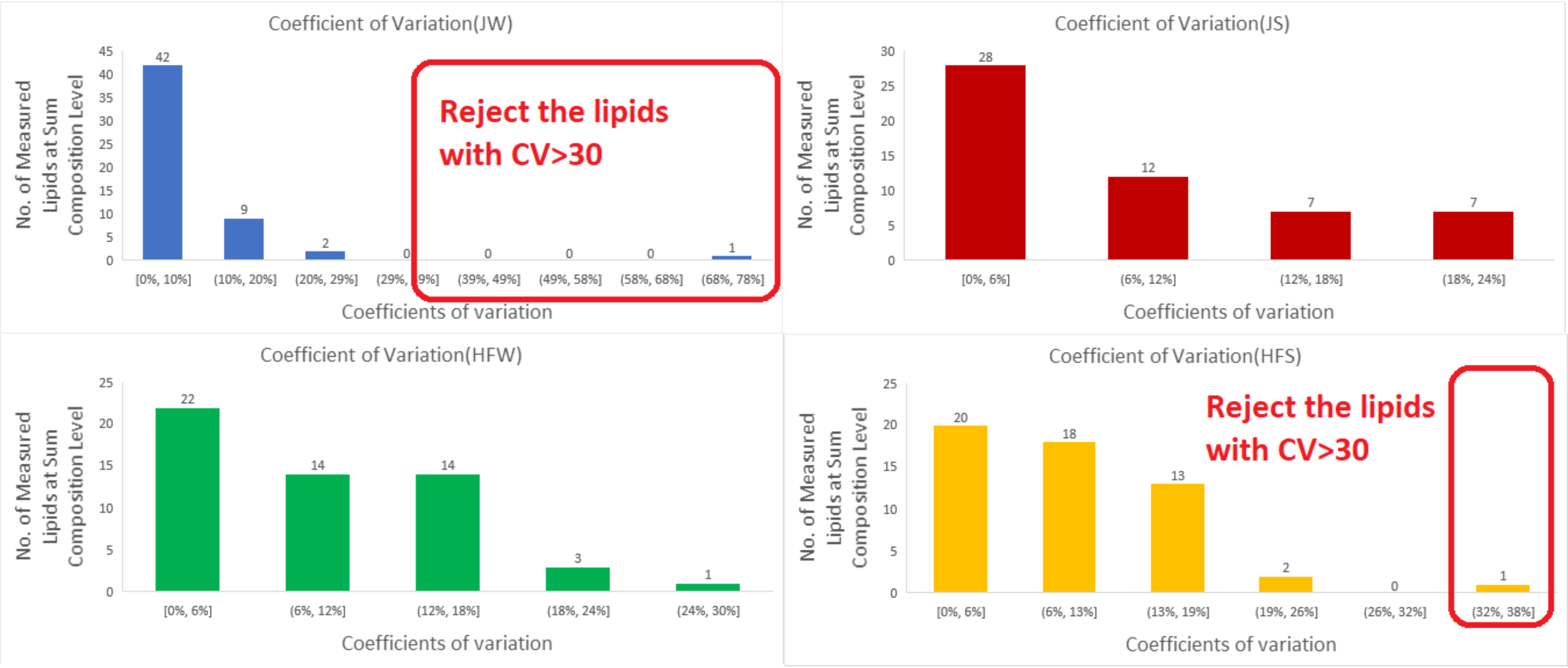

Figure S12(A) Variation of the 11 DG molecular groups between the breeds

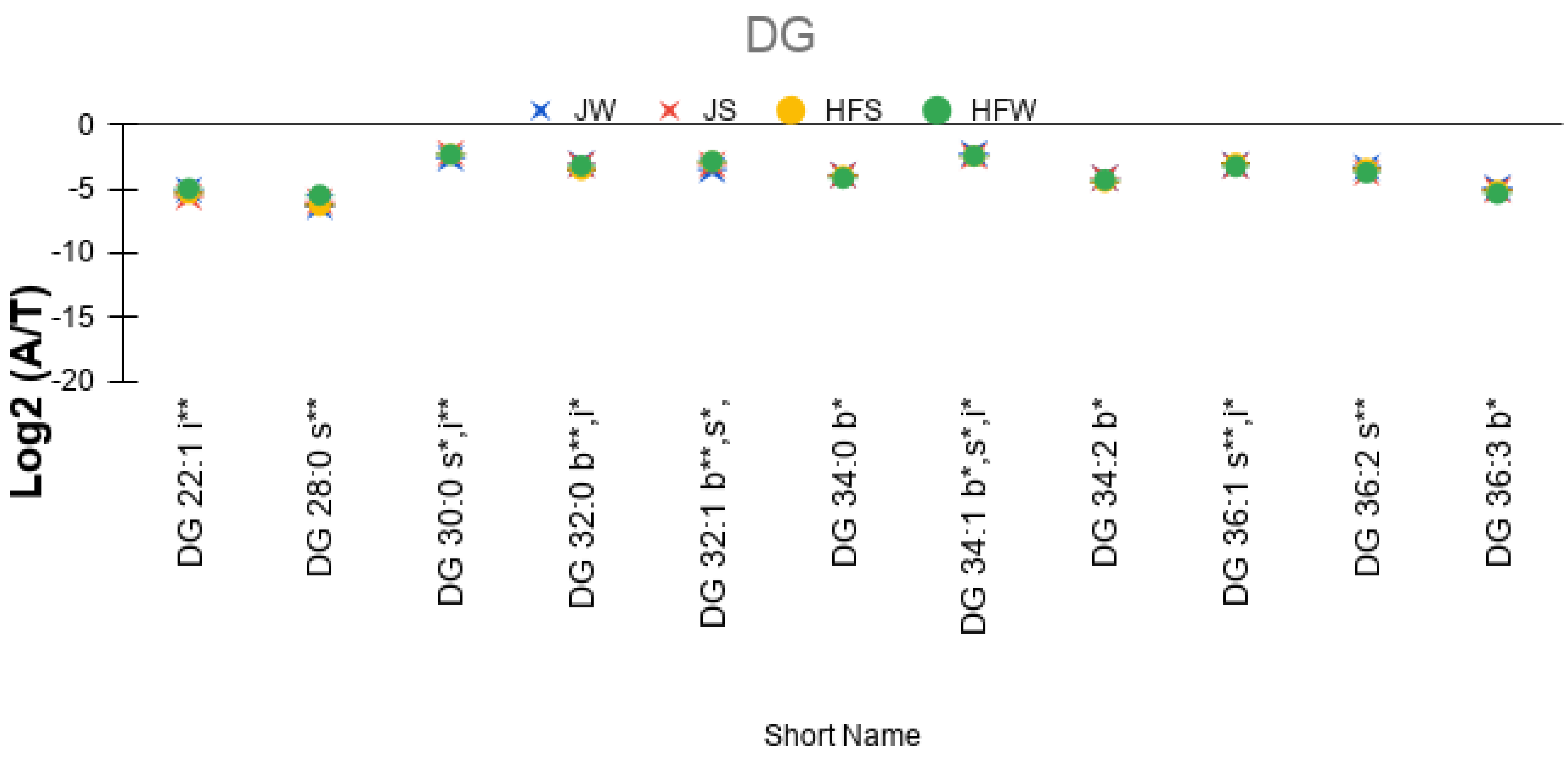

Figure S12(B) Variation of the 15 Glycerophospholipids –PC, PE, PS, and PI - between the breeds.

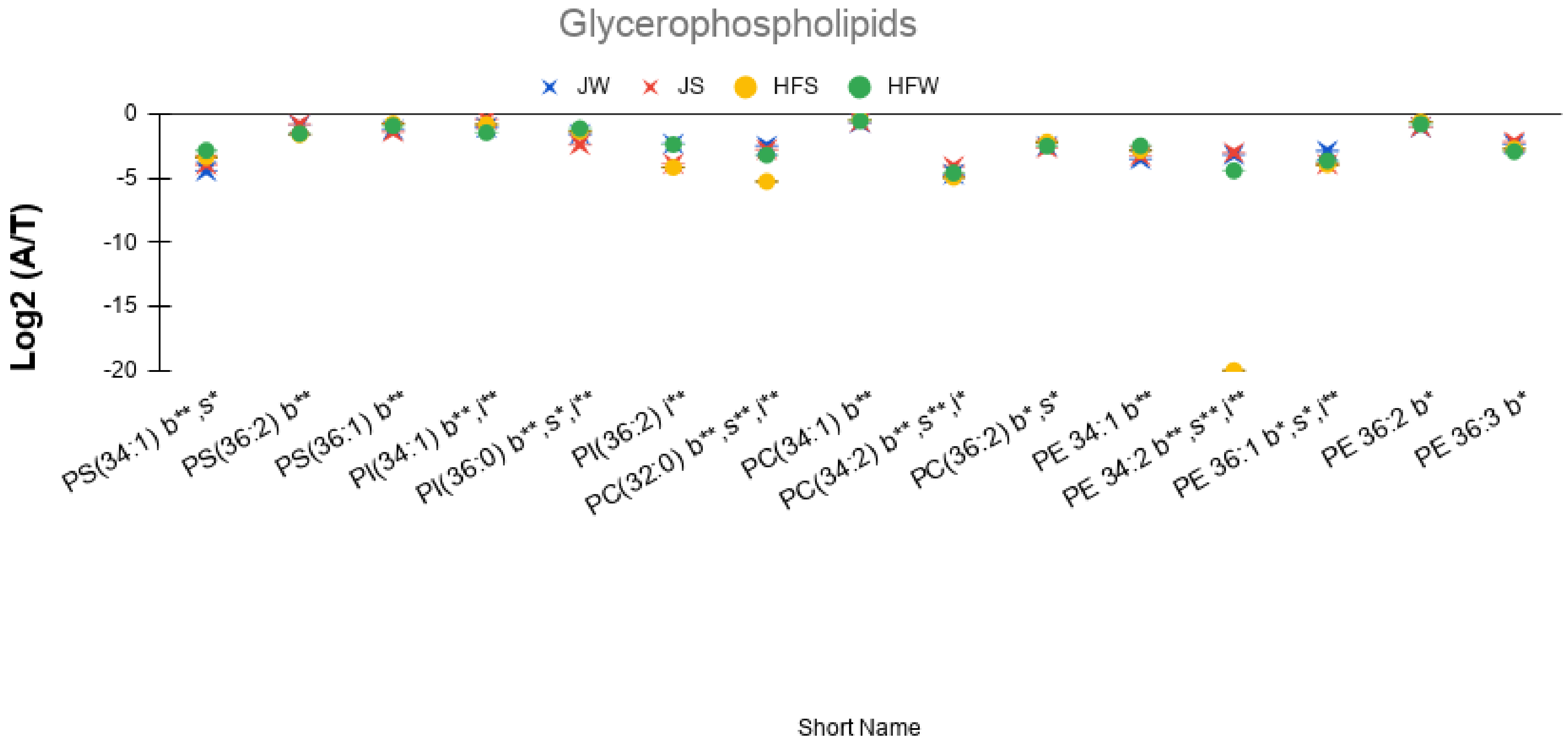

Figure S12**(**C**):** Variation of the 19 sphingomyelin lipid compositions between the breeds

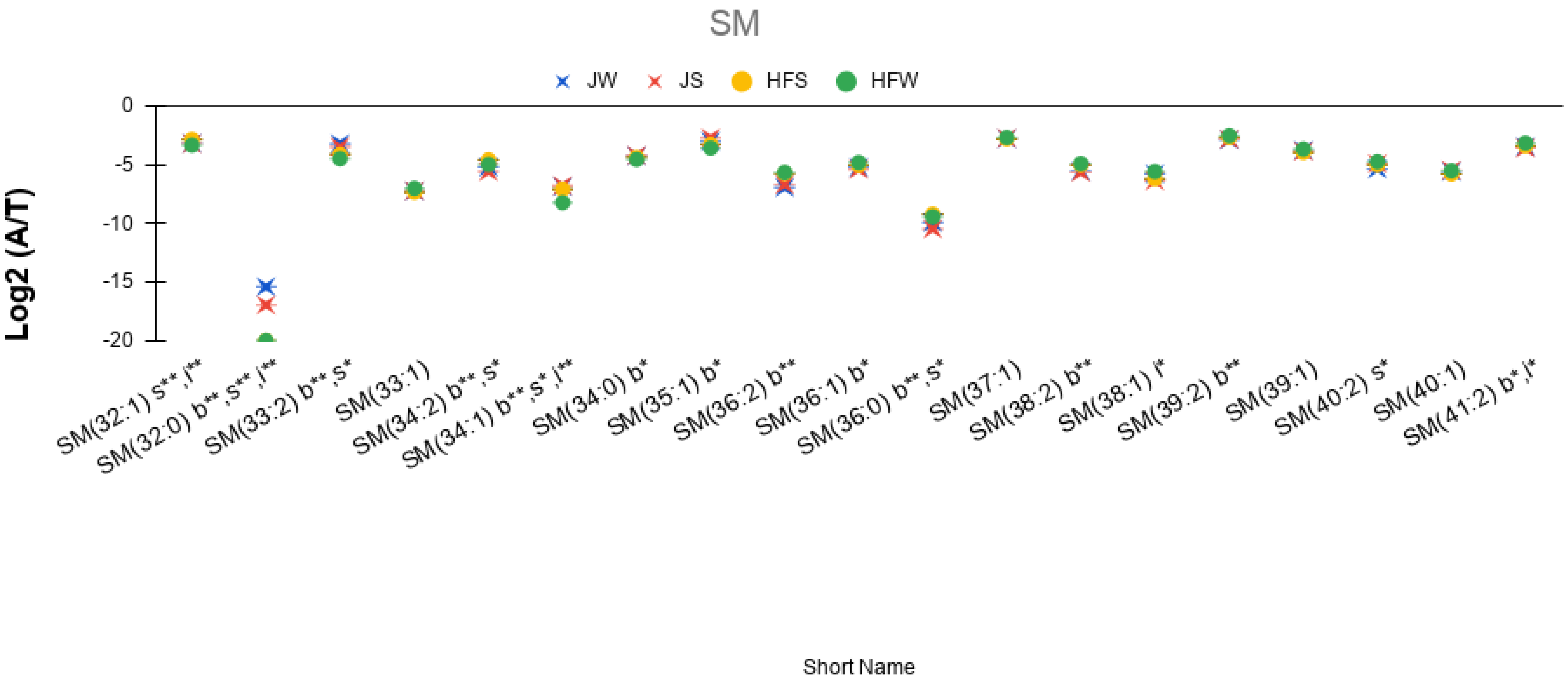

Figure S12(**D**)**:** Variation of the 7 Sterols molecular groups between the breeds.

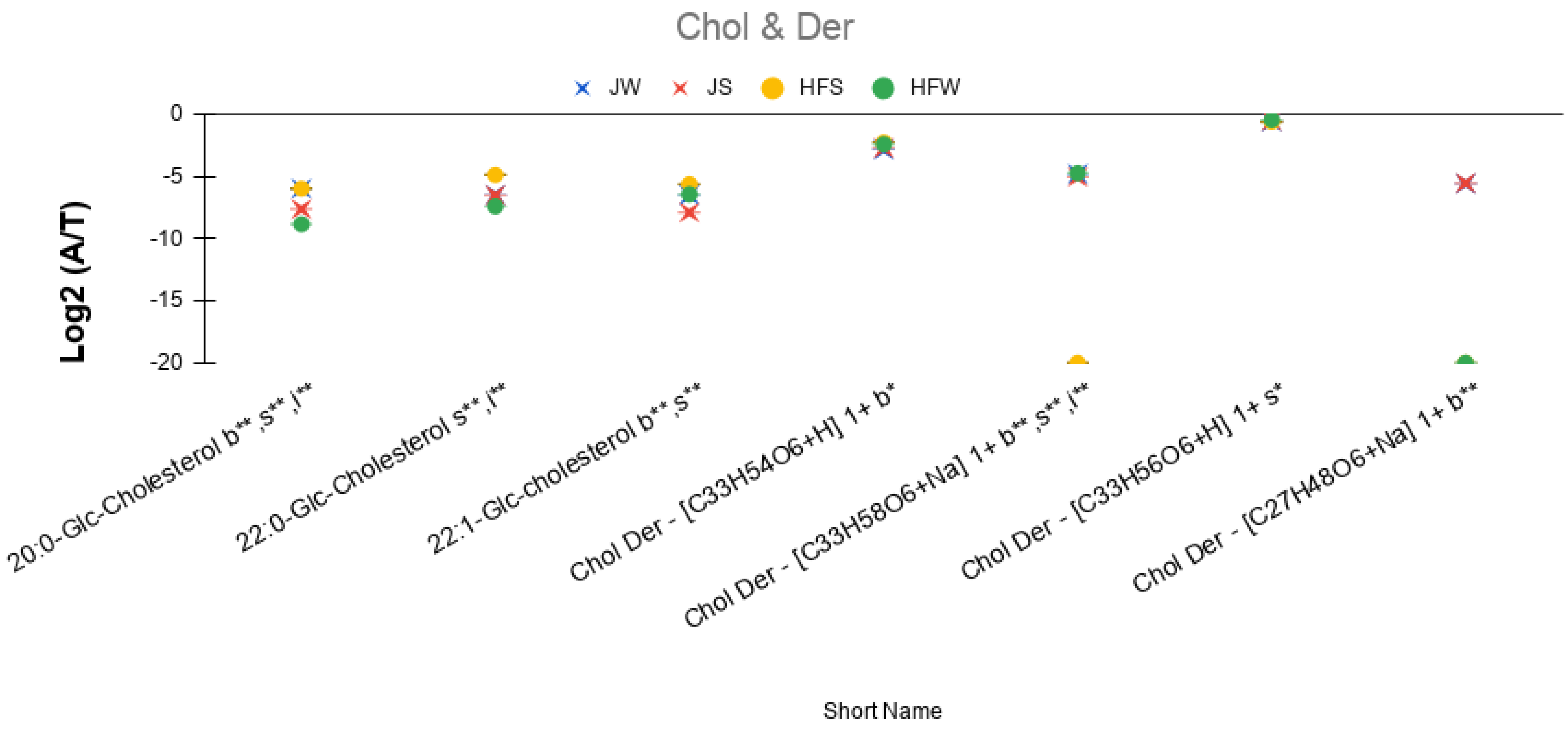

Table S1: Novel lipid species that are not yet reported by LIPIDMAPS database have been manually created based on the peaks observed in the MS/MS spectra.

1. TG 4:0_4:0_15:0
2. TG 4:0_6:0_14:0
3. TG 4:0_8:0_12:0
4. TG 4:0_4:0_16:0
5. TG 4:0_10:0_10:0
6. TG 2:0_6:0_16:0
7. TG 6:0_8:0_10:0
8. TG 2:0_4:0_18:0
9. TG 2:0_4:0_18:1
10. TG 4:0_4:0_16:1
11. TG 4:0_6:0_15:0
12. TG 4:0_5:0_16:0
13. TG 4:0_10:0_11:0
14. TG 6:0_9:0_10:0
15. TG 3:0_9:0_13:0
16. TG 4:0_6:0_16:0
17. TG 6:0_10:0_10:0
18. TG 4:0_10:0_12:0
19. TG 2:0_6:0_18:0
20. TG 4:0_4:0_18:1
21. TG 2:0_12:0_12:1
22. TG 4:0_4:0_18:2
23. TG 4:0_6:0_16:2
24. TG 4:0_8:0_15:0
25. TG 6:0_6:0_15:0
26. TG 4:0_6:0_17:0
27. TG 5:0_7:0_15:0
28. TG 5:0_8:0_14:0
29. TG 5:0_10:0_12:0
30. TG 4:0_10:0_14:0
31. TG 4:0_8:0_16:0
32. TG 6:0_10:0_12:0
33. TG 14:0_8:0_6:0
34. TG 4:0_6:0_18:0
35. TG 2:0_10:0_16:0
36. TG 4:0_6:0_18:1
37. TG 6:0_6:0_16:1
38. TG 4:0_8:0_16:1
39. TG 4:0_10:1_14:0

40. TG 4:0_10:0_14:1
41. TG 2:0_6:0_20:1
42. TG 4:0_6:0_18:2
43. TG 4:0_8:0_16:2
44. TG 4:0_10:0_15:0
45. TG 4:0_8:0_17:0
46. TG 5:0_10:0_14:0
47. TG 6:0_11:0_12:0
48. TG 7:0_9:0_13:0
49. TG 4:0_12:0_14:0
50. TG 10:0_12:0_8:0
51. TG 6:0_10:0_14:1
52. TG 4:0_12:0_14:1
53. TG 6:0_12:0_12:1
54. TG 8:0_10:0_12:1
55. TG 2:0_14:0_14:1
56. TG 4:0_8:0_18:2
57. TG 4:0_12:0_15:0
58. TG 6:0_12:0_13:0
59. TG 2:0_13:0_16:0
60. TG 8:0_10:0_13:0
61. TG 2:0_14:0_15:0
62. TG 4:0_12:0_16:0
63. TG 6:0_10:0_16:0
64. TG 10:0_10:0_12:0
65. TG 8:0_10:0_14:0
66. TG 2:0_14:0_16:0
67. TG 3:0_14:0_15:0
68. TG 4:0_13:0_15:0
69. TG 4:0_12:0_16:1
70. TG 10:0_10:1_12:0
71. TG 2:0_14:1_16:0
72. TG 2:0_14:0_16:1
73. TG 6:0_12:1_14:1
74. TG 6:0_10:0_16:2
75. TG 8:0_10:1_14:1
76. TG 10:0_10:1_12:1
77. TG 4:0_12:0_16:2
78. TG 4:0_14:0_15:0
79. TG 3:0_13:0_17:0

80. TG 8:0_12:0_13:0
81. TG 2:0_14:0_17:0
82. TG 10:0_11:0_12:0
83. TG 2:0_13:0_18:0
84. TG 4:0_14:0_16:0
85. TG 6:0_12:0_16:0
86. TG 8:0_12:0_14:0
87. TG 2:0_14:0_18:0
88. TG 10:0_12:0_12:0
89. TG 4:0_14:1_16:0
90. TG 6:0_14:0_14:1
91. TG 6:0_12:0_16:1
92. TG 10:0_10:1_14:0
93. TG 8:0_12:0_14:1
94. TG 2:0_16:0_16:1
95. TG 10:0_12:0_12:1
96. TG 2:0_14:1_18:0
97. TG 6:0_12:1_16:1
98. TG 4:0_15:0_16:0
99. TG 2:0_16:0_17:0
100. TG 2:0_15:0_18:0
101. TG 2:0_14:0_19:0
102. TG 4:0_13:0_18:1
103. TG 4:0_14:0_17:1
104. TG 5:0_14:0_16:1
105. TG 2:0_15:0_18:1
106. TG 5:0_15:0_15:1
107. TG 4:0_15:1_16:0
108. TG 7:0_14:0_14:1
109. TG 4:0_16:0_16:0
110. TG 6:0_14:0_16:0
111. TG 4:0_14:0_18:0
112. TG 10:0_10:0_16:0
113. TG 2:0_16:0_18:0
114. TG 10:0_12:0_14:0
115. TG 8:0_10:0_18:0
116. TG 4:0_16:0_16:1
117. TAG 6:0_12:0_18:1
118. TG 6:0_14:0_16:1
119. TG 2:0_16:0_18:1

120.	TG 2:0_16:1_18:0
121.	TG 8:0_10:0_18:1
122.	TG 8:0_12:0_16:1
123.	TG 10:0_12:0_14:1
124.	TG 4:0_14:0_18:2
125.	TG 4:0_14:1_18:1
126.	TG 6:0_14:1_16:1
127.	TG 6:0_14:0_16:2
128.	TG 8:0_12:0_16:2
129.	TG 10:0_10:1_16:1
130.	TG 10:0_12:1_14:1
131.	TG 4:0_14:0_18:3
132.	TG 4:0_16:1_16:2
133.	TG 4:0_16:0_17:0
134.	TG 4:0_15:0_18:0
135.	TG 6:0_15:0_16:0
136.	TG 6:0_14:0_17:0
137.	TG 5:0_16:0_16:0
138.	TG 5:0_14:0_18:0
139.	TG 10:0_12:0_15:0
140.	TG 4:0_15:0_18:1
141.	TG 4:0_16:0_17:1
142.	TG 4:0_14:0_19:1
143.	TG 3:0_16:0_18:1
144.	TG 6:0_14:0_17:1
145.	TG 3:0_17:0_17:1
146.	TG 4:0_15:0_18:2
147.	TG 4:0_15:1_18:1
148.	TG 4:0_16:1_17:1
149.	TG 4:0_16:0_18:0
150.	TG 6:0_16:0_16:0
151.	TG 6:0_14:0_18:0
152.	TG 8:0_14:0_16:0
153.	TG 10:0_12:0_16:0
154.	TG 2:0_18:0_18:0
155.	TG 4:0_16:0_18:1
156.	TG 6:0_14:0_18:1
157.	TG 10:0_12:0_16:1
158.	TG 10:0_10:0_18:1
159.	TG 8:0_12:0_18:1

160. TG 4:0_16:0_18:2
161. TG 4:0_16:1_18:1
162. TG 6:0_16:1_16:1
163. TG 6:0_14:0_18:2
164. TG 6:0_14:1_18:1
165. TG 4:0_16:0_18:3
166. TG 4:0_16:1_18:2
167. TG 4:0_17:0_18:0
168. TG 4:0_16:0_19:0
169. TG 6:0_15:0_18:0
170. TG 10:0_14:0_15:0
171. TG 4:0_17:0_18:1
172. TG 4:0_16:0_19:1
173. TG 6:0_15:0_18:1
174. TG 4:0_17:1_18:1
175. TG 6:0_15:0_18:2
176. TG 4:0_16:1_19:1
177. TG 5:0_16:0_18:2
178. TG 6:0_16:0_18:0
179. TG 10:0_14:0_16:0
180. TG 8:0_16:0_16:0
181. TG 4:0_18:0_18:0
182. TG 10:0_12:0_18:0
183. TG 8:0_14:0_18:0
184. TG 4:0_16:0_20:0
185. TG 4:0_14:0_22:0
186. TG 4:0_18:0_18:1
187. TG 6:0_16:0_18:1
188. TG 8:0_14:0_18:1
189. TG 10:0_14:0_16:1
190. TG 10:0_12:0_18:1
191. TG 4:0_18:1_18:1
192. TG 6:0_16:1_18:1
193. TG 6:0_16:0_18:2
194. TG 8:0_14:1_18:1
195. TG 4:0_18:1_18:2
196. TG 6:0_16:1_18:2
197. TG 4:0_16:0_20:4
198. TG 4:0_18:1_18:3
199. TAG 6:0_17:0_18:0

200. TG 10:0_15:0_16:0
201. TG 4:0_16:0_21:0
202. TG 11:0_14:0_16:0
203. TG 11:0_13:0_17:0
204. TG 6:0_14:0_21:0
205. TG 9:0_14:0_18:0
206. TG 6:0_17:0_18:1
207. TG 8:0_15:0_18:1
208. TG 4:0_18:0_19:1
209. TG 5:0_18:0_18:1
210. TG 4:0_18:1_19:1
211. TG 6:0_17:1_18:1
212. TG 6:0_17:0_18:2
213. TG 5:0_18:0_18:2
214. TG 8:0_18:0_16:0
215. TG 10:0_14:0_18:0
216. TG 6:0_18:0_18:0
217. TG 4:0_18:0_20:0
218. TG 6:0_18:0_18:1
219. TG 4:0_16:0_22:1
220. TG 6:0_18:1_18:1
221. TG 8:0_16:0_18:2
222. TG 8:0_14:0_20:2
223. TG 6:0_18:1_18:2
224. TG 4:0_18:1_20:2
225. TG 4:0_18:0_20:3
226. TG 6:0_18:1_18:3
227. TG 6:0_18:2_18:2
228. TG 6:0_16:0_20:4
229. TG 4:0_18:1_20:4
230. TG 4:0_16:0_22:5
231. TG 10:1_15:0_18:1
232. TG 10:1_16:0_17:1
233. TG 10:1_16:0_18:0
234. TG 8:0_18:1_18:2
235. TG 10:0_16:1_18:2
236. TG 10:0_16:0_18:3
237. TAG 10:0_14:0_20:3
238. TAG 10:0_17:1_18:1
239. TAG 10:0_18:0_18:1

240. TG 10:0_18:1_18:1
241. TG 10:0_18:1_18:2
242. TG 10:1_18:0_18:2
243. TG 10:0_18:1_18:3
244. TG 11:0_18:1_18:1
245. TG 16:0_16:0_24:0
246. TG 16:0_18:1_23:1
247. TG 18:0_18:1_23:1
248. TG 18:1_18:1_23:1
249. TG 18:1_18:1_24:1
250. TG 18:0_18:1_25:0
251. TG 19:0_19:1_23:0
252. TG 16:0_19:1_26:0
253. TG 16:0_18:0_28:0
254. TG 16:0_16:0_30:0
255. TG 16:0_18:1_28:0
256. TG 18:0_18:1_26:0
257. TG 16:0_20:1_26:0
258. TG 16:0_22:1_24:0
259. TG 18:0_18:2_26:0
260. TG 16:0_18:2_28:0
261. TG 14:0_16:0_22:5

Table S1: List of Lipid IDs across Breeds and Seasons

A. Lipid IDs common between Jafarabadi Summer (JS) and Holstein Friesian Winter (HW)

1.	TG 10:0_12:0_12:1
2.	TG 14:1_14:1_16:1
3.	TG 12:0_12:0_21:0
4.	TG 15:0_15:0_18:2
5.	TG 14:0_16:0_20:1
6.	TG 14:0_18:1_20:3
7.	TG 14:0_16:0_22:4
8.	TG 16:0_17:0_20:1
9.	TG 15:0_18:0_20:1
10.	TG 16:0_17:1_20:1
11.	TG 16:0_16:0_22:4
12.	TG 16:1_18:0_22:0
13.	TG 16:1_20:0_20:0
14.	TG 16:1_18:1_22:1
15.	TG 16:1_20:1_20:1
16.	TG 14:0_21:0_22:1
17.	TG 17:1_18:0_22:1
18.	TG 17:1_19:1_21:0
19.	TG 18:0_20:1_22:1
20.	TG 16:1_22:0_22:1
21.	TG 16:0_22:1_22:1
22.	TG 18:1_20:1_22:1

B. Lipid IDs common between Jafarabadi Winter (JW) and Holstein Friesian Summer (HS)

1.	DG 4:0_18:1_0:0
2.	TG 2:0_14:0_15:0
3.	TG 4:0_12:0_16:2
4.	TG 4:0_14:0_22:0
5.	TG 6:0_16:1_18:2
6.	TG 11:0_14:0_16:0
7.	TG 11:0_13:0_17:0
8.	TG 12:0_14:1_15:0
9.	TG 12:0_13:0_16:1
10	TG 8:0_14:0_20:2

11	TG 4:0_16:0_22:5
12	TG 12:0_14:1_17:0
13	TG 14:0_14:0_15:1
14	TG 10:0_14:0_20:3
15	TG 12:0_16:1_17:1
16	TG 14:1_15:0_16:1
17	TG 14:1_14:1_17:0
18	TG 12:0_14:0_20:1
19	TG 12:0_16:0_18:3
20	TG 10:0_18:1_18:3
21	TG 13:0_18:1_18:1
22	TG 13:0_18:0_18:2
23	TG 15:1_16:1_18:0
24	TG 15:0_16:0_18:3
25	TG 12:0_18:0_20:0
26	TG 12:0_16:0_22:0
27	TG 15:0_15:0_20:0
28	TG 15:0_17:2_18:2
29	TG 14:0_14:0_22:4
30	TG 14:0_15:0_22:0
31	TG 15:1_18:0_18:0
32	TG 16:1_17:0_18:2
33	TG 14:0_16:0_22:5
34	TG 16:1_18:2_18:2
35	TG 14:0_18:1_21:0
36	TG 15:0_16:0_22:1
37	TG 14:0_19:1_20:0
38	TG 14:0_18:0_22:1
39	TG 15:0_18:0_22:1
40	TG 16:0_18:1_22:2
41	TG 18:0_18:3_20:1
42	TG 18:2_18:2_20:1
43	TG 17:0_20:0_20:0
44	TG 16:0_19:0_22:1
45	TG 18:1_19:1_20:1
46	TG 19:1_19:1_19:1
47	TG 18:1_19:1_22:0
48	TG 16:0_22:0_22:0
49	TG 19:1_19:1_22:1
50	TG 20:0_20:0_22:1
51	TG 20:0_20:1_22:0

C. Lipid IDs in Jafarabadi Summer (JS) and Holstein Friesian Summer (HS)

1.	TG 13:0_15:0_18:1
2.	TG 13:0_16:0_18:0
3.	TG 13:0_17:0_17:0
4.	TG 15:0_17:1_19:1
5.	TG 14:0_18:0_20:4
6.	TG 16:0_18:0_18:4
7.	TG 17:2_18:0_18:1
8.	TG 17:0_17:2_19:1
9.	TG 14:0_20:0_21:0
10	TG 16:0_20:0_20:2
11	TG 18:0_18:0_20:2
12	SM d43:1

D. Lipid IDs in Holstein Friesian Summer (HS) and Jafarabadi Winter (JW)

1.	TG 10:0_12:1_14:1
2.	TG 12:0_12:0_20:1
3.	TG 13:0_13:0_19:0
4.	TG 14:1_16:0_17:0
5.	TG 14:0_15:1_18:1
6.	TG 15:1_15:1_17:0
7.	TG 14:1_18:0_18:1
8.	TG 15:0_16:0_20:3
9.	TG 14:0_20:0_20:0
10	TG 16:0_18:1_20:5
11	TG 14:0_19:0_22:0
12	TG 17:1_18:0_20:0
13	TG 14:0_19:0_22:1
14	TG 16:1_20:1_20:2
15	TG 16:1_18:1_22:2
16	TG 18:0_18:0_20:4
17	TG 16:0_18:0_22:4
18	TG 16:1_20:1_22:1
19	TAG 16:0_20:1_22:2

E. Lipid IDs common in all four samples

1.	DG 14:0_16:0_0:0
2.	DG 12:0_18:0_0:0
3.	DG 16:0_16:0_0:0
4.	DG 14:0_18:0_0:0
5.	DG 14:0_18:1_0:0
6.	DG 16:0_16:1_0:0
7.	DG 18:0_16:0_0:0
8.	DG 18:1_16:0_0:0
9.	DG 18:0_18:1_0:0
10.	DG 18:1_18:1_0:0
11.	TG 4:0_6:0_14:0
12.	TG 4:0_8:0_12:0
13.	TG 4:0_4:0_16:0
14.	TG 4:0_10:0_10:0
15.	TG 2:0_6:0_16:0
16.	TG 6:0_8:0_10:0
17.	TG 4:0_6:0_16:0
18.	TG 6:0_10:0_10:0
19.	TG 4:0_10:0_12:0
20.	TG 2:0_6:0_18:0
21.	TG 4:0_4:0_18:1
22.	TG 4:0_10:0_14:0
23.	TG 4:0_8:0_16:0
24.	TG 6:0_10:0_12:0
25.	TG 14:0_8:0_6:0
26.	TG 4:0_6:0_18:0
27.	TG 2:0_10:0_16:0
28.	TG 4:0_6:0_18:1
29.	TG 6:0_6:0_16:1
30.	TG 4:0_8:0_16:1
31.	TG 4:0_10:1_14:0
32.	TG 4:0_10:0_14:1
33.	TG 4:0_10:0_15:0
34.	TG 5:0_10:0_14:0
35.	TG 6:0_11:0_12:0
36.	TG 4:0_12:0_14:0
37.	TG 10:0_12:0_8:0
38.	TG 6:0_10:0_14:1
39.	TG 4:0_12:0_14:1
40.	TG 6:0_12:0_12:1
41.	TG 8:0_10:0_12:1

42.	TG 2:0_14:0_14:1
43.	TG 4:0_12:0_15:0
44.	TG 6:0_12:0_13:0
45.	TG 8:0_10:0_13:0
46.	TG 4:0_12:0_16:0
47.	TG 6:0_10:0_16:0
48.	TG 10:0_10:0_12:0
49.	TG 8:0_10:0_14:0
50.	TG 2:0_14:0_16:0
51.	TG 3:0_14:0_15:0
52.	TG 4:0_13:0_15:0
53.	TG 4:0_12:0_16:1
54.	TG 10:0_10:1_12:0
55.	TG 2:0_14:1_16:0
56.	TG 2:0_14:0_16:1
57.	TG 4:0_14:0_15:0
58.	TG 3:0_13:0_17:0
59.	TG 8:0_12:0_13:0
60.	TG 2:0_14:0_17:0
61.	TG 10:0_11:0_12:0
62.	TG 2:0_13:0_18:0
63.	TG 4:0_14:0_16:0
64.	TG 6:0_12:0_16:0
65.	TG 8:0_12:0_14:0
66.	TG 2:0_14:0_18:0
67.	TG 10:0_12:0_12:0
68.	TG 4:0_14:1_16:0
69.	TG 6:0_14:0_14:1
70.	TG 6:0_12:0_16:1
71.	TG 10:0_10:1_14:0
72.	TG 8:0_12:0_14:1
73.	TG 2:0_16:0_16:1
74.	TG 2:0_14:1_18:0
75.	TG 6:0_12:1_16:1
76.	TG 4:0_15:0_16:0
77.	TG 4:0_13:0_18:1
78.	TG 4:0_14:0_17:1
79.	TG 5:0_14:0_16:1
80.	TG 2:0_15:0_18:1
81.	TG 5:0_15:0_15:1
82.	TG 6:0_14:0_15:1
83.	TG 4:0_16:0_16:0
84.	TG 6:0_14:0_16:0
85.	TG 4:0_14:0_18:0

86.	TG 10:0_10:0_16:0
87.	TG 2:0_16:0_18:0
88.	TG 10:0_12:0_14:0
89.	TG 8:0_10:0_18:0
90.	TG 4:0_16:0_16:1
91.	TG 6:0_12:0_18:1
92.	TG 6:0_14:0_16:1
93.	TG 2:0_16:0_18:1
94.	TG 2:0_16:1_18:0
95.	TG 8:0_10:0_18:1
96.	TG 8:0_12:0_16:1
97.	TG 10:0_12:0_14:1
98.	TG 4:0_14:0_18:2
99.	TG 4:0_14:1_18:1
100.	TG 6:0_14:1_16:1
101.	TG 6:0_14:0_16:2
102.	TG 4:0_14:0_18:3
103.	TG 4:0_16:0_17:0
104.	TG 4:0_15:0_18:0
105.	TG 6:0_15:0_16:0
106.	TG 6:0_14:0_17:0
107.	TG 5:0_16:0_16:0
108.	TG 5:0_14:0_18:0
109.	TG 10:0_12:0_15:0
110.	TG 12:0_12:0_13:0
111.	TG 4:0_15:0_18:1
112.	TG 4:0_16:0_17:1
113.	TG 4:0_14:0_19:1
114.	TG 6:0_14:0_17:1
115.	TG 4:0_15:0_18:2
116.	TG 4:0_15:1_18:1
117.	TG 4:0_16:1_17:1
118.	TG 4:0_16:0_18:0
119.	TG 6:0_16:0_16:0
120.	TG 6:0_14:0_18:0
121.	TG 8:0_14:0_16:0
122.	TG 10:0_12:0_16:0
123.	TG 12:0_12:0_14:0
124.	TG 4:0_16:0_18:1
125.	TG 6:0_14:0_18:1
126.	TG 10:0_12:0_16:1
127.	TG 10:0_10:0_18:1
128.	TG 12:0_12:0_14:1
129.	TG 8:0_12:0_18:1

130.	TG 4:0_16:0_18:2
131.	TG 4:0_16:1_18:1
132.	TG 6:0_16:1_16:1
133.	TG 6:0_14:0_18:2
134.	TG 6:0_14:1_18:1
135.	TG 4:0_16:0_18:3
136.	TG 4:0_16:1_18:2
137.	TG 4:0_17:0_18:0
138.	TG 4:0_16:0_19:0
139.	TG 6:0_15:0_18:0
140.	TG 10:0_14:0_15:0
141.	TG 12:0_12:0_15:0
142.	TG 12:0_13:0_14:0
143.	TG 13:0_13:0_13:0
144.	TG 4:0_17:0_18:1
145.	TG 4:0_16:0_19:1
146.	TG 6:0_15:0_18:1
147.	TG 4:0_17:1_18:1
148.	TG 6:0_15:0_18:2
149.	TG 4:0_16:1_19:1
150.	TG 5:0_16:0_18:2
151.	TG 6:0_16:0_18:0
152.	TG 10:0_14:0_16:0
153.	TG 8:0_16:0_16:0
154.	TG 4:0_18:0_18:0
155.	TG 12:0_12:0_16:0
156.	TG 10:0_12:0_18:0
157.	TG 8:0_14:0_18:0
158.	TG 12:0_14:0_14:0
159.	TG 13:0_13:0_14:0
160.	TG 4:0_16:0_20:0
161.	TG 4:0_18:0_18:1
162.	TG 6:0_16:0_18:1
163.	TG 8:0_14:0_18:1
164.	TG 10:0_14:0_16:1
165.	TG 10:0_12:0_18:1
166.	TG 12:0_12:0_16:1
167.	TG 4:0_18:1_18:1
168.	TG 6:0_16:1_18:1
169.	TG 6:0_16:0_18:2
170.	TG 4:0_18:1_18:2
171.	TG 4:0_18:1_18:3
172.	TG 6:0_17:0_18:0
173.	TG 10:0_15:0_16:0

174.	TG 12:0_14:0_15:0
175.	TG 12:0_13:0_16:0
176.	TG 13:0_13:0_15:0
177.	TG 12:0_12:0_17:0
178.	TG 13:0_14:0_14:0
179.	TG 6:0_17:0_18:1
180.	TG 8:0_15:0_18:1
181.	TG 4:0_18:0_19:1
182.	TG 5:0_18:0_18:1
183.	TG 4:0_18:1_19:1
184.	TG 6:0_17:1_18:1
185.	TG 6:0_17:0_18:2
186.	TG 12:0_14:0_16:0
187.	TG 8:0_18:0_16:0
188.	TG 10:0_14:0_18:0
189.	TG 6:0_18:0_18:0
190.	TG 12:0_12:0_18:0
191.	TG 14:0_14:0_14:0
192.	TG 12:0_13:0_17:0
193.	TG 12:0_15:0_15:0
194.	TG 13:0_13:0_16:0
195.	TG 6:0_18:0_18:1
196.	TG 12:0_12:0_18:1
197.	TG 12:0_14:0_16:1
198.	TG 4:0_16:0_22:1
199.	TG 6:0_18:1_18:1
200.	TG 8:0_16:0_18:2
201.	TG 6:0_18:1_18:2
202.	TG 4:0_18:1_20:2
203.	TG 12:0_15:0_16:0
204.	TG 13:0_14:0_16:0
205.	TG 14:0_14:0_15:0
206.	TG 12:0_14:0_17:0
207.	TG 12:0_15:0_16:1
208.	TG 12:0_14:0_17:1
209.	TG 12:0_13:0_18:1
210.	TG 12:0_12:0_19:1
211.	TG 13:0_14:0_16:1
212.	TG 14:0_14:0_16:0
213.	TG 12:0_16:0_16:0
214.	TG 12:0_14:0_18:0
215.	TG 14:0_15:0_15:0
216.	TG 13:0_15:0_16:0
217.	TG 12:0_14:0_18:1

218.	TG 12:0_16:0_16:1
219.	TG 14:0_14:1_16:0
220.	TG 12:0_14:1_18:0
221.	TG 10:1_16:0_18:0
222.	TG 14:0_14:0_16:1
223.	TG 12:0_16:1_16:1
224.	TG 8:0_18:1_18:2
225.	TG 14:0_15:0_16:0
226.	TG 12:0_16:0_17:0
227.	TG 13:0_16:0_16:0
228.	TG 14:0_14:0_17:0
229.	TG 12:0_15:0_18:0
230.	TG 13:0_14:0_18:0
231.	TG 13:0_15:0_17:0
232.	TG 15:0_15:0_15:0
233.	TG 12:0_15:0_18:1
234.	TG 14:1_15:0_16:0
235.	TG 13:0_14:0_18:1
236.	TG 14:0_15:0_16:1
237.	TG 12:0_16:0_17:1
238.	TG 12:0_14:0_19:1
239.	TG 14:0_14:1_17:0
240.	TG 12:0_16:1_17:0
241.	TG 13:0_15:0_17:1
242.	TG 14:0_14:0_17:1
243.	TG 14:0_16:0_16:0
244.	TG 12:0_16:0_18:0
245.	TG 15:0_15:0_16:0
246.	TG 14:0_14:0_18:0
247.	TG 14:0_15:0_17:0
248.	TG 12:0_16:0_18:1
249.	TG 14:0_14:0_18:1
250.	TG 14:0_16:0_16:1
251.	TG 14:1_16:0_16:0
252.	TG 10:0_18:0_18:1
253.	TG 12:0_16:1_18:0
254.	TG 14:0_14:1_18:0
255.	TG 14:0_14:1_18:1
256.	TG 12:0_16:0_18:2
257.	TG 10:0_18:1_18:1
258.	TG 14:0_14:0_18:2
259.	TG 12:0_16:1_18:1
260.	TG 14:1_16:0_16:1
261.	TG 14:0_16:1_16:1

262.	TG 10:0_18:1_18:2
263.	TG 14:1_14:1_18:1
264.	TG 15:0_16:0_16:0
265.	TG 14:0_16:0_17:0
266.	TG 14:0_15:0_18:0
267.	TG 15:0_15:0_17:0
268.	TG 14:0_15:0_18:1
269.	TG 13:0_16:0_18:1
270.	TG 12:0_17:0_18:1
271.	TG 14:0_16:0_17:1
272.	TG 15:0_16:0_16:1
273.	TG 14:0_16:1_17:0
274.	TG 15:0_15:0_17:1
275.	TG 13:0_15:0_19:1
276.	TG 14:0_14:0_19:1
277.	TG 12:0_16:0_19:1
278.	TG 13:0_17:0_17:1
279.	TG 14:1_15:0_18:1
280.	TG 14:0_15:0_18:2
281.	TG 14:0_14:1_19:1
282.	TG 15:0_16:1_16:1
283.	TG 14:0_16:1_17:1
284.	TG 14:0_16:0_18:0
285.	TG 16:0_16:0_16:0
286.	TG 15:0_16:0_17:0
287.	TG 15:0_15:0_18:0
288.	TG 14:0_17:0_17:0
289.	TG 14:0_16:0_18:1
290.	TG 16:0_16:0_16:1
291.	TG 14:0_16:1_18:0
292.	TG 14:0_16:0_18:2
293.	TG 14:0_16:1_18:1
294.	TG 16:0_16:1_16:1
295.	TG 12:0_18:1_18:1
296.	TG 12:0_18:1_18:2
297.	TG 14:0_16:1_18:2
298.	TG 14:1_16:1_18:1
299.	TG 14:1_16:0_18:2
300.	TG 16:1_16:1_16:1
301.	TG 15:0_16:0_18:0
302.	TG 16:0_16:0_17:0
303.	TG 14:0_17:0_18:0
304.	TG 15:0_17:0_17:0
305.	TG 15:0_16:0_18:1

306.	TG 14:0_17:0_18:1
307.	TG 16:0_16:1_17:0
308.	TG 16:0_16:0_17:1
309.	TG 15:0_17:0_17:1
310.	TG 15:0_16:1_18:0
311.	TG 14:0_17:1_18:0
312.	TG 15:0_16:0_18:2
313.	TG 15:0_16:1_18:1
314.	TG 14:0_17:1_18:1
315.	TG 15:1_16:0_18:1
316.	TG 16:0_16:1_17:1
317.	TG 15:0_17:1_17:1
318.	TG 15:1_17:0_17:1
319.	TG 16:1_16:1_17:0
320.	TG 16:1_16:1_17:1
321.	TG 16:0_16:0_18:0
322.	TG 14:0_18:0_18:0
323.	TG 16:0_16:0_18:1
324.	TG 14:0_18:0_18:1
325.	TG 16:0_16:1_18:1
326.	TG 14:0_18:1_18:1
327.	TG 14:0_18:0_18:2
328.	TG 16:0_16:0_18:2
329.	TG 16:1_16:1_18:0
330.	TG 14:0_18:1_18:2
331.	TG 14:1_18:1_18:1
332.	TG 16:1_16:1_18:1
333.	TG 16:0_16:1_18:2
334.	TG 16:0_16:0_18:3
335.	TG 14:0_16:0_20:3
336.	TG 14:1_18:1_18:2
337.	TG 14:0_18:2_18:2
338.	TG 14:0_16:0_20:4
339.	TG 16:0_17:0_18:0
340.	TG 15:0_18:0_18:0
341.	TG 15:0_17:0_19:0
342.	TG 16:0_16:0_19:0
343.	TG 17:0_17:0_17:0
344.	TG 16:0_17:0_18:1
345.	TG 15:0_18:0_18:1
346.	TG 15:0_17:0_19:1
347.	TG 16:0_17:1_18:0
348.	TG 16:0_16:0_19:1
349.	TG 17:0_17:0_17:1

350.	TG 16:0_17:1_18:1
351.	TG 15:0_18:1_18:1
352.	TG 16:1_17:0_18:1
353.	TG 15:0_18:0_18:2
354.	TG 16:0_17:1_18:2
355.	TG 15:0_18:1_18:2
356.	TG 15:1_18:1_18:1
357.	TG 16:1_17:1_18:1
358.	TG 17:1_17:1_17:1
359.	TG 16:0_18:0_18:0
360.	TG 14:0_18:0_20:0
361.	TG 17:0_17:0_18:0
362.	TG 16:0_16:0_20:0
363.	TG 16:0_18:0_18:1
364.	TG 16:0_16:0_20:1
365.	TG 17:0_17:0_18:1
366.	TG 16:0_18:1_18:1
367.	TG 16:1_18:0_18:1
368.	TG 16:0_18:0_18:2
369.	TG 16:0_18:1_18:2
370.	TG 16:1_18:1_18:1
371.	TG 17:1_17:1_18:1
372.	TG 16:0_16:0_20:3
373.	TG 16:1_18:1_18:2
374.	TG 16:0_18:2_18:2
375.	TG 16:0_18:1_18:3
376.	TG 16:0_16:0_20:4
377.	TG 16:0_18:0_19:0
378.	TG 17:0_18:0_18:0
379.	TG 16:0_17:0_20:0
380.	TG 16:0_16:0_21:0
381.	TG 17:0_17:0_19:0
382.	TG 17:0_18:0_18:1
383.	TG 16:0_18:1_19:0
384.	TG 16:0_18:0_19:1
385.	TG 17:1_18:0_18:0
386.	TG 17:0_17:1_19:0
387.	TG 17:0_17:0_19:1
388.	TG 17:1_18:0_18:1
389.	TG 17:0_18:1_18:1
390.	TG 16:0_18:1_19:1
391.	TG 17:0_17:1_19:1
392.	TG 17:0_18:0_18:2
393.	TG 16:0_18:2_19:0

394.	TG 17:1_17:1_19:0
395.	TG 17:1_18:1_18:1
396.	TG 17:0_18:1_18:2
397.	TG 16:0_18:2_19:1
398.	TG 17:1_17:1_19:1
399.	TG 17:1_18:1_18:2
400.	TG 17:0_18:1_18:3
401.	TG 17:0_18:2_18:2
402.	TG 16:0_18:0_20:0
403.	TG 18:0_18:0_18:0
404.	TG 16:0_16:0_22:0
405.	TG 18:0_18:0_18:1
406.	TG 16:0_18:0_20:1
407.	TG 16:0_18:1_20:0
408.	TG 18:0_18:1_18:1
409.	TG 18:0_18:0_18:2
410.	TG 18:1_18:1_18:1
411.	TG 18:0_18:1_18:2
412.	TG 18:1_18:1_18:2
413.	TG 18:0_18:2_18:2
414.	TG 18:0_18:1_18:3
415.	TG 16:0_18:1_20:3
416.	TG 16:0_18:1_20:4
417.	TG 18:1_18:2_18:2
418.	TG 18:1_18:1_18:3
419.	TG 16:0_18:0_21:0
420.	TG 16:0_17:0_22:0
421.	TG 16:0_19:0_20:0
422.	TG 18:0_18:0_19:0
423.	TG 17:0_18:0_20:0
424.	TG 15:0_18:0_22:0
425.	TG 17:0_17:0_21:0
426.	TG 16:0_18:1_21:0
427.	TG 18:0_18:1_19:0
428.	TG 16:1_18:0_21:0
429.	TG 17:0_18:1_20:0
430.	TG 16:0_19:0_20:1
431.	TG 16:0_19:1_20:0
432.	TG 18:0_18:0_19:1
433.	TG 17:0_18:0_20:1
434.	TG 17:0_19:0_19:1
435.	TG 16:1_17:0_22:0
436.	TG 18:0_18:1_19:1
437.	TG 18:1_18:1_19:0

438.	TG 18:1_18:1_19:1
439.	TG 16:0_18:0_22:0
440.	TG 18:0_18:0_20:0
441.	TG 16:0_20:0_20:0
442.	TG 16:0_16:0_24:0
443.	TG 18:0_18:1_20:0
444.	TG 16:0_18:1_22:0
445.	TG 16:0_18:0_22:1
446.	TG 16:0_20:0_20:1
447.	TG 18:0_18:0_20:1
448.	TG 16:0_18:1_22:1
449.	TG 18:0_18:1_20:1
450.	TG 18:1_18:1_20:0
451.	TG 16:1_18:1_22:0
452.	TG 16:0_20:1_20:1
453.	TG 16:1_20:0_20:1
454.	TG 18:0_18:2_20:0
455.	TG 16:1_18:0_22:1
456.	TG 18:0_19:1_19:1
457.	TG 16:0_18:2_22:0
458.	TG 18:1_18:1_20:1
459.	TG 18:1_18:2_20:0
460.	TG 18:0_18:1_20:2
461.	TG 18:0_18:2_20:1
462.	TG 16:0_20:1_20:2
463.	TG 18:1_18:1_20:2
464.	TG 18:0_18:1_20:3
465.	TG 18:0_18:1_20:4
466.	TG 18:1_18:1_20:3
467.	TG 16:0_18:0_22:5
468.	TG 18:1_18:1_20:4
469.	TG 16:0_18:1_22:5
470.	TG 18:0_18:0_21:0
471.	TG 17:0_18:0_22:0
472.	TG 18:0_18:1_21:0
473.	TG 16:0_18:1_23:1
474.	TG 17:0_18:1_22:1
475.	TG 18:1_18:1_21:0
476.	TG 15:0_20:1_22:1
477.	TG 17:0_20:1_20:1
478.	TG 18:0_18:0_22:0
479.	TG 18:0_20:0_20:0
480.	TG 16:0_20:0_22:0
481.	TG 18:0_18:1_22:0

482.	TG 18:0_18:0_22:1
483.	TG 18:0_18:1_22:1
484.	TG 18:1_18:1_22:0
485.	TG 18:1_18:1_22:1
486.	TG 18:0_18:2_22:1
487.	TG 18:1_20:0_22:0
488.	TG 18:0_20:0_22:1
489.	TG 16:0_22:0_22:1
490.	TG 18:1_20:1_22:0
491.	TG 18:1_18:1_24:1
492.	TG 16:0_18:1_28:0
493.	TG 18:0_18:1_26:0
494.	Cho Der - [C33H54O6+H] 1+
495.	Cho Der - [C33H56O6+H] 1+
496.	Steryl esters [C43H74O7+H] 1+

F. Lipid IDs found only in Jafarabadi (J)

1.	TG 4:0_4:0_15:0
2.	TG 2:0_4:0_18:0
3.	TG 2:0_4:0_18:1
4.	TG 4:0_4:0_16:1
5.	TG 4:0_4:0_18:2
6.	TG 4:0_6:0_16:2
7.	TG 4:0_6:0_18:2
8.	TG 4:0_8:0_16:2
9.	TG 4:0_16:1_16:2
10.	TG 2:0_18:0_18:0
11.	TG 4:0_16:0_21:0
12.	TG 9:0_14:0_18:0
13.	TG 5:0_18:0_18:2
14.	TG 4:0_18:1_20:4
15.	TG 12:0_16:0_19:0
16.	TG 14:0_14:0_19:0
17.	TG 15:0_15:1_17:0
18.	TG 13:0_16:1_18:1
19.	TG 13:0_16:0_18:2
20.	TG 13:0_17:1_17:1
21.	TG 12:0_18:0_18:2
22.	TG 16:0_16:1_18:3

23.	TG 1:0_16:0_18:4
24.	TG 14:0_18:0_21:0
25.	TG 14:0_17:0_22:0
26.	TG 14:0_19:0_20:0
27.	TG 16:1_18:1_19:1
28.	TG 17:2_18:1_18:1
29.	TG 17:1_18:1_19:1
30.	TG 15:0_19:1_21:0
31.	TG 16:0_18:2_21:0
32.	TG 18:1_18:2_19:0
33.	TG 18:0_18:2_19:1
34.	TG 14:0_20:1_22:0
35.	TG 18:0_18:2_21:0
36.	TG 18:0_18:2_22:0
37.	TG 16:1_20:0_22:2
38.	TG 16:0_16:0_30:0

G. Lipid IDs found only in Holstein Friesian (H)

1.	TG 12:0_14:1_14:1
2.	TG 12:0_12:0_20:0
3.	TG 14:1_15:0_15:0
4.	TG 14:1_14:1_16:0
5.	TG 14:1_14:1_18:0
6.	TG 14:1_16:1_17:0
7.	TG 14:1_16:0_18:0
8.	TG 15:1_15:1_19:0
9.	TG 15:0_16:0_20:0
10.	TG 14:0_17:0_20:0
11.	TG 15:0_17:0_21:0
12.	TG 16:0_19:1_20:1
13.	TG 17:1_18:1_20:1
14.	TG 17:1_19:1_19:1
15.	TG 16:0_18:0_22:3
16.	TG 16:0_18:3_22:0
17.	TG 18:0_19:1_22:0
18.	TG 19:1_20:0_20:0
19.	TG 18:0_20:1_22:0
20.	TG 20:0_20:0_20:1
21.	TG 20:0_20:1_20:1
22.	SM d32:1
23.	SM d38:2

24.	SM d42:2

H. Lipid IDs in Jafarabadi Summer (JS)

1.	TG 12:0_15:0_19:1
2.	TG 12:0_17:0_17:1
3.	TG 14:0_15:0_17:1
4.	TG 13:0_15:0_19:0
5.	TG 12:0_14:0_21:0
6.	TG 13:0_13:0_21:0
7.	TG 14:0_15:0_20:1
8.	TG 15:1_17:1_17:1
9.	TG 15:1_16:1_18:1
10.	TG 14:0_14:0_22:1
11.	TG 15:0_15:0_20:1
12.	TG 14:0_18:1_19:0
13.	TG 14:0_18:0_19:1
14.	TG 14:0_18:1_19:1
15.	TG 15:1_17:1_19:1
16.	TG 17:1_17:1_18:0
17.	TG 17:0_17:2_18:2
18.	TG 16:1_18:1_19:0
19.	TG 16:1_18:0_19:1
20.	TG 15:1_19:0_19:1
21.	TG 14:1_19:0_20:1
22.	TG 15:1_18:0_20:1
23.	TG 16:1_19:0_19:0
24.	TG 16:0_19:1_19:1
25.	TG 17:2_18:2_19:0
26.	TG 17:1_18:1_21:0
27.	TG 16:0_19:1_22:0
28.	TG 17:0_19:1_21:0
29.	TG 17:1_18:1_22:0
30.	TG 18:1_19:1_20:0
31.	TG 16:1_19:1_22:0
32.	TG 17:0_18:2_22:0
33.	TG 17:1_20:0_20:1
34.	TG 18:0_18:0_22:2
35.	PE 34:2
36.	SM d33:2
37.	SM d41:2

38.	SM dd36:0
39.	SM dd36:2

I. Lipid IDs in Jafarabadi Winter (JW)

1.	DG 16:0_20:1_0:0
2.	DG 18:1_18:2_0:0
3.	TG 4:0_5:0_16:0
4.	TG 4:0_10:0_11:0
5.	TG 6:0_9:0_10:0
6.	TG 3:0_9:0_13:0
7.	TG 2:0_6:0_20:1
8.	TG 7:0_9:0_13:0
9.	TG 8:0_12:0_16:2
10.	TG 6:0_14:0_21:0
11.	TG 13:0_14:1_16:0
12.	TG 12:0_14:0_18:3
13.	TG 12:0_12:0_20:3
14.	TG 13:0_14:1_18:1
15.	TG 13:0_14:0_18:2
16.	TG 14:0_14:1_17:1
17.	TG 13:0_16:1_16:1
18.	TG 12:0_14:1_19:1
19.	TG 12:0_12:0_22:0
20.	TG 12:0_16:1_18:2
21.	TG 12:0_14:0_20:3
22.	TG 14:1_15:0_18:0
23.	TG 15:0_15:1_17:1
24.	TG 13:0_15:1_19:1
25.	TG 12:0_18:0_18:0
26.	TG 12:0_14:0_22:0
27.	TG 12:0_16:0_20:4
28.	TG 12:0_18:1_18:3
29.	TG 12:0_18:2_18:2
30.	TG 14:0_16:0_19:0
31.	TG 14:0_15:0_20:0
32.	TG 15:0_15:0_19:0
33.	TG 12:0_18:0_19:0
34.	TG 15:1_16:0_18:2
35.	TG 13:0_18:1_18:2
36.	TG 15:0_16:0_19:0
37.	TG 14:0_17:0_19:0
38.	TG 16:0_18:0_16:0

39.	TG 14:0_16:1_20:3
40.	TG 16:1_17:0_18:0
41.	TG 15:0_18:1_18:3
42.	TG 15:0_18:2_18:2
43.	TG 16:0_17:0_19:0
44.	TG 14:0_19:0_19:0
45.	TG 16:1_17:0_19:0
46.	TG 16:0_17:0_19:1
47.	TG 16:0_17:1_19:0
48.	TG 17:0_17:1_18:0
49.	TG 17:1_17:1_18:2
50.	TG 14:0_18:1_20:4
51.	TG 16:0_18:2_18:3
52.	TG 16:1_18:1_18:3
53.	TG 16:0_18:1_18:4
54.	TG 16:1_16:1_20:3
55.	TG 17:1_17:2_19:1
56.	TG 18:1_18:2_18:3
57.	TG 18:2_18:2_18:2
58.	TG 17:1_18:1_20:0
59.	TG 16:0_19:1_20:2
60.	TG 18:1_18:2_19:1
61.	TG 18:1_18:3_19:0
62.	TG 18:2_18:2_19:0
63.	TG 18:1_19:0_19:0
64.	TG 16:1_18:0_22:3
65.	TG 18:1_18:2_20:3
66.	TG 16:0_18:2_22:4
67.	TG 16:0_20:3_20:3
68.	TG 18:1_20:0_20:2
69.	TG 18:2_20:0_20:1
70.	TG 16:0_20:2_22:1
71.	TG 16:1_20:2_22:0
72.	TG 18:0_20:1_20:2
73.	TG 18:0_20:0_21:0
74.	TG 18:0_19:0_22:0
75.	TG 16:0_21:0_22:0
76.	TG 17:0_20:0_22:0
77.	TG 19:0_20:0_20:0
78.	TG 19:0_19:0_21:0
79.	TG 17:0_21:0_21:0
80.	TG 18:0_20:0_22:0
81.	TG 20:0_20:0_20:0
82.	TG 19:0_19:1_23:0

83.	TG 16:0_19:1_26:0
84.	TG 20:0_20:1_21:0
85.	TG 19:1_21:0_21:0
86.	TG 16:0_18:0_28:0
87.	TG 18:0_18:2_26:0
88.	Cho Der - [C27H44O6+H] 1+
89.	Cho Der - [C27H48O6+Na] 1+
90.	Cho Der - [C33H58O6+Na] 1+
91.	Cho Der - 20:0
92.	Cho Der - 22:0
93.	Cho Der - 22:1

J. Lipid IDs in Holstein Friesian Summer (HS)

1.	TG 12:0_14:1_19:0
2.	TG 15:1_15:1_16:0
3.	TG 12:0_17:0_18:2
4.	TG 15:0_15:0_17:2
5.	TG 14:0_16:0_17:2
6.	TG 13:0_17:0_18:0
7.	TG 12:0_16:1_20:1
8.	TG 16:0_16:0_17:2
9.	TG 16:0_17:1_17:1
10.	TG 16:0_16:1_19:1
11.	TG 16:1_17:0_20:0
12.	TG 18:0_18:0_18:4
13.	TG 15:0_19:0_21:0
14.	TG 16:0_17:1_22:0
15.	TG 17:0_17:1_21:0
16.	TG 17:1_19:0_19:0
17.	TG 18:0_18:2_20:3
18.	TG 18:0_18:1_22:3
19.	TG 18:1_21:0_21:0
20.	TG 16:1_22:0_22:0

K. Lipid IDs in Holstein Friesian Winter (HW)

1.	DG 12:0_16:0_0:0
2.	TG 6:0_12:1_14:1
3.	TG 7:0_14:0_14:1
4.	TG 13:0_14:0_14:1
5.	TG 12:0_12:0_17:1
6.	TG 12:0_14:1_16:1
7.	TG 14:0_14:1_14:1
8.	TG 14:1_14:1_15:0
9.	TG 12:0_12:0_22:1
10.	TG 12:0_14:1_20:0
11.	TG 14:1_15:0_17:0
12.	TG 10:1_18:0_18:2
13.	TG 12:0_15:0_20:1
14.	TG 13:0_14:0_20:1
15.	TG 11:0_18:1_18:1
16.	TG 14:0_15:0_19:0
17.	TG 15:0_16:0_17:1
18.	TG 13:0_17:1_19:1
19.	TG 14:1_17:1_18:1
20.	TG 15:0_17:0_18:1
21.	TG 16:0_17:0_17:1
22.	TG 15:0_16:0_19:1
23.	TG 14:0_17:0_19:1
24.	TG 15:0_17:1_18:0
25.	TG 16:1_17:0_17:0
26.	TG 14:1_18:0_18:2
27.	TG 14:0_18:0_18:3
28.	TG 15:1_18:0_18:1
29.	TG 15:1_17:1_19:0
30.	TG 15:1_18:0_18:2
31.	TG 14:0_16:0_22:1
32.	TG 14:0_18:1_20:0
33.	TG 14:0_18:0_20:1
34.	TG 14:0_18:1_20:1
35.	TG 16:0_16:1_20:1
36.	TG 14:0_16:1_22:1
37.	TG 16:1_17:1_19:0
38.	TG 16:0_16:0_20:2
39.	TG 14:0_16:0_22:2

40.	TG 14:1_16:0_22:1
41.	TG 14:0_18:0_20:2
42.	TG 14:1_18:0_20:1
43.	TG 16:1_16:1_20:2
44.	TG 14:1_18:1_20:2
45.	TG 14:0_17:0_22:1
46.	TG 14:0_19:0_20:1
47.	TG 14:0_19:1_20:1
48.	TG 17:0_18:1_19:0
49.	TG 14:0_18:1_22:0
50.	TG 14:0_20:0_20:1
51.	TG 17:0_17:0_20:1
52.	TG 16:1_18:0_20:1
53.	TG 16:0_16:1_22:1
54.	TG 16:0_16:0_22:2
55.	TG 14:0_18:0_22:2
56.	TG 14:0_18:1_22:1
57.	TG 17:1_18:1_19:0
58.	TG 14:1_18:0_22:1
59.	TG 16:0_18:0_20:2
60.	TG 14:0_20:1_20:1
61.	TG 17:0_18:1_20:1
62.	TG 15:0_18:1_22:1
63.	TG 16:1_17:0_22:1
64.	TG 16:1_19:0_20:1
65.	TG 15:0_20:1_20:1
66.	TG 14:0_19:1_22:1
67.	TG 17:0_19:1_19:1
68.	TG 15:0_18:1_22:2
69.	TG 16:1_19:1_20:1
70.	TG 17:0_18:2_20:1
71.	TG 15:0_18:2_22:1
72.	TG 16:1_17:1_22:1
73.	TG 16:1_17:0_22:2
74.	TG 17:0_17:0_22:1
75.	TG 18:1_19:0_19:1
76.	TG 18:2_19:0_19:0
77.	TG 16:1_18:0_22:2
78.	TG 16:1_20:0_20:2
79.	TG 18:1_18:3_20:0
80.	TG 18:2_18:2_20:0
81.	TG 15:0_20:0_22:0
82.	TG 15:0_21:0_21:0
83.	TG 18:1_19:0_20:0

84.	TG 15:0_20:0_22:1
85.	TG 15:0_20:1_22:0
86.	TG 17:0_20:0_20:1
87.	TG 16:1_19:0_22:0
88.	TG 16:0_20:1_21:0
89.	TG 16:1_20:0_21:0
90.	TG 17:1_19:0_21:0
91.	TG 17:1_20:0_20:0
92.	TG 17:1_18:1_22:1
93.	TG 17:0_18:1_22:2
94.	TG 17:0_18:2_22:1
95.	TG 17:1_18:2_22:0
96.	TG 18:1_19:0_20:2
97.	TG 16:1_20:2_21:0
98.	TG 16:0_19:1_22:2
99.	TG 16:1_19:1_22:1
100	TG 17:1_18:0_22:2
101	TG 18:0_19:1_20:2
102	TG 16:0_21:0_21:0
103	TG 16:0_20:1_22:0
104	TG 14:0_22:0_22:1
105	TG 18:0_20:0_20:1
106	TG 18:1_20:0_20:1
107	TG 16:1_20:0_22:1
108	TG 18:1_18:2_22:1
109	TG 18:1_18:1_22:2
110	TG 18:2_20:1_20:1
111	TG 18:1_19:0_22:0
112	TG 18:1_20:0_21:0
113	TG 18:0_19:0_22:1
114	TG 18:0_20:1_21:0
115	TG 16:0_21:0_22:1
116	TG 17:0_20:0_22:1
117	TG 17:0_20:1_22:0
118	TG 16:1_21:0_22:0
119	TG 19:0_20:0_20:1
120	TG 19:0_19:1_21:0
121	TG 18:0_19:1_22:1
122	TG 18:1_19:1_22:1
123	TG 19:0_19:0_22:1
124	TG 18:1_20:0_22:1
125	TG 19:0_19:1_22:1
126	TG 18:2_20:0_22:0
127	TG 18:0_20:2_22:0

128	TG 19:1_19:1_22:0
129	TG 20:0_20:0_20:2
130	TG 16:0_22:1_22:2
131	TG 18:0_20:2_22:1
132	TG 18:0_20:1_22:2
133	TG 19:1_20:2_21:0
134	TG 18:0_21:0_22:1
135	TG 18:1_21:0_22:0
136	TG 19:0_20:0_22:1
137	TG 18:1_22:0_22:1
138	TG 18:0_22:1_22:1
139	TG 16:0_18:2_28:0
140	TG 20:1_20:1_22:0
141	TG 18:2_22:0_22:0
142	PC 34:1
143	SM d34:2

Abbreviation List

1.	FA	Fatty acid
2.	LA	Linoleic acid
3.	ALA	α-linolenic acid (ALA)
4.	PUFA	Polyunsaturated fatty acids
5.	(GC-MS)	Gas chromatography coupled with a mass spectrometer
6.	(LC-MS/MS)	Liquid chromatography coupled tandem mass spectrometry (LC-MS/MS)
7.	UPLC	Ultra-performance liquid chromatography
8.	J	Jafarabadi
9.	HF	Holstein Friesian
10.	FFA	Free fatty acids
11.	HRAM	High-resolution accurate mass
12.	JW	Jafarabadi winter
13.	JS	Jafarabadi summer
14.	HFS	Holstein Friesian summer
15.	HFW	Holstein Friesian winter
16.	MS	Mass spectrometry
17.	PA-ID	Partial-acyl identification
18.	FA-ID	Full-acyl identification
19.	RI	Relative intensity
20.	PA-ID	Partial-acyl identification
21.	TG	Triacylglycerol
22.	DG	Diacylglycerol
23.	LM	Lipids Maps
24.	SDF	Structure delay format
25.	PS	Phosphatidylserine
26.	PC	Phosphatidylcholine
27.	PE	Phosphoethanolamine
28.	PI	Phosphatidylinositol
29.	XIC	Extracted ion chromatogram
30.	QQ	Quantile-Quantile
31.	HSD	Honestly significant difference
32.	GUI	Graphical user interface
33.	SM	Sphingomylein
34.	Chol-Der	Cholesterol derivatives
35.	PCA	Principal Component Analysis
36.	PC1	Principal Component 1
37.	PC2	Principal Component 2
38.	HCA	Hierarchical Cluster Analysis
39.	ANOVA	Two-Way Analysis of Variance Analysis
40.	DDA	Data-dependent acquisition
41.	SFA	Saturated fatty acids
42.	CV	Coefficient of variations
43.	AFP	Average fat percentage
44.	HESI	Heated electrospray source

45.	HCD	Higher-energy collisional dissociation
46.	NCE	Normalized collision energy
47.	AGC	Automatic gain control
